# Supplementary material for: Association between long-term use of calcium channel blockers (CCB) and the risk of breast cancer: a retrospective longitudinal observational study protocol
Source: BMJ Open. 2024 Mar 8;14(3):e080982. doi: 10.1136/bmjopen-2023-080982 (PMC10928765; doi:10.1136/bmjopen-2023-080982)
Supplement: Supplementary data [file bmjopen-2023-080982supp001.pdf]

BMJ Publishing Group Limited (BMJ) disclaims all liability and responsibility arising from any reliance placed on this supplemental material which has been supplied by the author(s)


**Title: Association between long-term use of calcium channel blockers (CCB) and the risk of breast cancer: A retrospective longitudinal observational study protocol**

**Appendix Table 1. Classification of prescribed oral antihypertensive medicines used based on the Anatomical Therapeutic Chemical codes**

| Class | ATC code | Drug name | Usual dose range mg/day | DDD (mg) | Usual daily frequency* |
|---|---|---|---|---|---|
| ACEIs | C09AA01 | Captopril | 25-100 | 50 | 2 |
| | C09AA02 | Enalapril | 5-40 | 10 | 1-2 |
| | C09AA09 | Fosinopril | 10-40 | 15 | 1 |
| | C09AA03 | Lisinopril | 10-40 | 10 | 1 |
| | C09AA04 | Perindopril | 4-8 | 4 | 1 |
| | C09AA06 | Quinapril | 10-80 | 15 | 1-2 |
| | C09AA05 | Ramipril | 2.5-20 | 2.5 | 1-2 |
| | C09AA10 | Trandolapril | 1-4 | 2 | 1 |
| | C09AA07 | Benazepril | 10-40 | 7.5 | 1 |
| | C09AA13 | Moexipril | 7.5-30 | 15 | 1 |
| ARBs | C09CA06 | Candesartan | 8-32 | 8 | 1 |
| | C09CA02 | Eprosartan | 400-800 | 600 | 1-2 |
| | C09CA04 | Irbesartan | 150-300 | 150 | 1 |
| | C09CA01 | Losartan | 25-100 | 50 | 1-2 |
| | C09CA08 | Olmesartan Medoxomil | 20-40 | 20 | 1 |
| | C09CA07 | Telmisartan | 20-80 | 40 | 1 |
| | C09CA03 | Valsartan | 80-320 | 80 | 1-2 |
| BBs | C07AB03 | Atenolol | 25-100 | 75 | 1-2 |
| | C07AB05 | Betaxolol | 5-20 | 20 | 1 |
| | C07AB07 | Bisoprolol | 2.5-10 | 10 | 1 |
| | C07AG02 | Carvedilol | 12.5-50 | 37.5 | 2 |
| | C07AG01 | Labetalol | 200-800 | 600 | 2 |
| | C07AB02 | Metoprolol<br>Metoprolol ER | 50-100<br>50-100 | 150<br>150 | 1-2<br>1 |
| | C07AA12 | Nadolol | 40-120 | 160 | 1 |
| | C07AB12 | Nebivolol | 5 | 5 | 1 |
| | C07AA02 | Oxprenolol | 40-160 | 160 | 2 |
| | C07AA03 | Pindolol | 10-40 | 15 | 2-3 |
| | C07AA05 | Propranolol<br>Propranolol LA | 40-160<br>160-180 | 160<br>160 | 2<br>1 |
| | C07AA06 | Timolol | 20-40 | 20 | 2 |
| | C07AB04 | Acebutolol | 200-800 | 400 | 2 |
| | C07AA23 | Penbutolol | 10-40 | 40 | 1 |
| CCBs-DHP | C08CA01 | Amlodipine | 2.5-10 | 5 | 1 |
| | C08CA02 | Felodipine | 2.5-20 | 5 | 1 |
| | C08CA13 | Lercanidipine | 10-20 | 10 | 1 |
| | C08CA05 | Nifedipine<br>Nifedipine LA | 10-40<br>20-120 | 30 | 2<br>1 |
| | C08CA03 | Isradipine | 2.5-10 | 5 | 2 |
| | C08CA04 | Nicardipine ER | 60-120 | 90 | 2 |

| Class | ATC code | Drug name | Usual dose range mg/day | DDD (mg) | Usual daily frequency* |
|---|---|---|---|---|---|
| | C08CA07 | Nisoldipine | 10-40 | 20 | 1 |
| | C10BX03 | Atorvastatin And Amlodipine * | - | - | 1 |
| CCB-nonDHP | C08DA01 | Verapamil<br>Verapamil LA<br>Verapamil IR | 120-360<br>120-480<br>80-320 | 240<br>240<br>240 | 1<br>1-2<br>2 |
| | C08DB01 | Diltiazem ER<br>Diltiazem LA<br>Diltiazem | 180-420<br>120-540<br>180-240 | 240 | 1<br>1<br>3-4 |
| Diuretics | C03DB01 | Amiloride | 5-10 | 10 | 1-2 |
| | C03EA01 | Amiloride + HCTZ<br>Triamterene + HCTZ | - | - | 1-2 |
| | C03DB02 | Triamterene | 50-100 | 100 | 1-2 |
| | C03AA04 | Chlorothiazide | 125-500 | 500 | 1-2 |
| | C03AA05 | Polythiazide | 2-4 | 1 | 1 |
| | C03AA01 | Bendroflumethiazide | 2.5-10 | 2.5 | 1 |
| | C03AA03 | HCTZ | 12.5-50 | 25 | 1 |
| | C03BA04 | Chlortalidone | 12.5-25 | 25 | 1 |
| | C03BA11 | Indapamide | 1.25-2.5 | 2.5 | 1 |
| | C03BA08 | Metolazone** | 0.5-1.0<br>2.5-5 | 5 | 1 |
| | C03CA01 | Furosemide ** | 20-80 | 40 | 2 |
| | C03CA02 | Bumetanide ** | 0.5-2 | 1 | 2 |
| | C03CA04 | Torsemide ** | 2.5-10 | 15 | 1 |
| | C03DA01 | Spironolactone ** | 25-50 | 75 | 1 |
| | C03DA04 | Eplerenone ** | 50-100 | 50 | 1-2 |
| | C03CC01 | Etacrynic acid | 50-200 | 50 | 1-2 |
| | C03XA01 | Tolvaptan | 15-60 | 30 | 1 |
| Other ATH | C02AC01 | Clonidine | 0.1-0.8 | 0.5 | 2 |
| | C02AC02 | Guanfacine | 0.5-2 | 3 | 1 |
| | C02DB02 | Hydralazine | 25-100 | 100 | 2 |
| | C02AB01 | Methyldopa | 250-1000 | 1000 | 2-4 |
| | C02AA02 | Reserpine | 0.1-0.25 | 0.5 | 1 |
| | C02DC01 | Minoxidil | 2.5-80 | 20 | 1-2 |
| | C02AC05 | Moxonidine | 0.2-0.6 | 0.3 | 1-2 |
| | C02CA01 | Prazosin | 2-20 | 5 | 2-3 |
| | C02CA04 | Doxazosin | 1-16 | 4 | 1 |
| | G04CA03 | Terazosin | 1-20 | 5 | 1-2 |
| Combination | C09BB02 | Lercanidipine + Enalapril | - | - | 1 |
| | C09BB04 | Perindopril + Amlodipine | - | - | 1 |
| | C09BB05 | Ramipril + Felodipine | - | - | 1 |
| | C09BB10 | Trandolapril + Verapamil | - | - | 1 |
| | C09BA09 | Fosinopril + HCTZ | - | - | 1 |

| Class | ATC code | Drug name | Usual dose range mg/day | DDD (mg) | Usual daily frequency* |
|---|---|---|---|---|---|
| | C09BA04 | Perindopril + Indapamide | - | - | 1 |
| | C09BA02 | Enalapril + HCTZ | - | - | 1 |
| | C09BA06 | Quinapril + HCTZ | - | - | 1 |
| | C09DX04 | Valsartan + Sacubitril | - | - | 2 |
| | C09DB01 | Amlodipine + Valsartan | - | - | 1 |
| | C09DB02 | Olmesartan + Amlodipine | - | - | 1 |
| | C09DB04 | Telmisartan + Amlodipine | - | - | 1 |
| | C09DX01 | Amlodipine + Valsartan + HCTZ | - | - | 1 |
| | C09DX03 | Olmesartan + Amlodipine + HCTZ | - | - | 1 |
| | C09DA06 | Candesartan + HCTZ | - | - | 1 |
| | C09DA02 | Eprosartan + HCTZ | - | - | 1 |
| | C09DA04 | Irbesartan + HCTZ | - | - | 1 |
| | C09DA08 | Olmesartan + HCTZ | - | - | 1 |
| | C09DA07 | Telmisartan + HCTZ | - | - | 1 |
| | C09DA03 | Valsartan +HCTZ | - | - | 1 |

HCTZ: hydrochlorothiazide. ATC: Anatomical Therapeutic Chemical, RAS: Renin Angiotensin System, CCB-DHP: calcium channel blockers-dihydropyridines, CCB-nonDHP: calcium channel blockers-nondihydropyridines, BB: beta blockers, ER: extended release, LA: long acting, IR: immediate release, DDD: Define Daily Dose, *: usual daily frequency is based on recommendations by the seventh report of the joint national committee on prevention, detection, evaluation, and treatment of high blood pressure (2003), **: These drugs are used for other indications (but have an antihypertensive effect).
