# Supplementary material for: Association between long-term use of calcium channel blockers (CCB) and the risk of breast cancer: a retrospective longitudinal observational study protocol
Source: BMJ Open. 2024 Mar 8;14(3):e080982. doi: 10.1136/bmjopen-2023-080982 (PMC10928765; doi:10.1136/bmjopen-2023-080982)
Supplement: Supplementary data [file bmjopen-2023-080982supp002.pdf]

Title: Association between long-term use of calcium channel blockers (CCB) and the risk of breast cancer: A retrospective longitudinal observational study protocol

Appendix Table 2. Codes used to identify participants' diagnosis and procedures in linked data sources

Conditions	MBS item number (for diagnoses or procedures recorded in Australian MBS data)	Procedure codes - ACHI 10 (for procedures recorded in Australian hospital admission data)	ATC codes (for medicines recorded in pharmacy data and study examination - Rotterdam)	ICD code (for diagnoses recorded in study examination-Rotterdam, hospital, cancer, and mortality data)	
				ICD-9-CM/ ICD-9	ICD-10-AM disease code/ ICD-10
**Diabetes**	MBS claims for:<br>- Diabetes annual cycle of care: 2517, 2518, 2521, 2522, 2525, 2526, 2620, 2622, 2624, 2631, 2633, 2635, 259 – 264.<br>- HbA1C test: 66551, 73840<br>- Comprehensive reassessment of eyes in a patient with diabetes mellitus: 10915, 12325, 12326.<br>- Diabetes education: 81100, 81105<br>- Exercise physiology health service for the management of type 2 diabetes: 81110, 81115<br>- Dietetics health service for the management of type 2 diabetes: 81120, 81125	-	+ Anti-diabetic Therapies: A10	+ Secondary diabetes melllitus:249<br>+ Diabetes mellitus: 250	+ Type 1 diabetes mellitus: E10<br>+ Type 2 diabetes mellitus: E11<br>+ Other specified diabetes Mellitus: E13<br>+Unspecified diabetes mellitus: E14
**Heart disease**	MBS claims for:<br>-Percutaneous transluminal coronary angioplasty, item 35304, 35305, 35310, 35335, 35338, 35341, 35344, 38300, 38303, 38306 38309, 38312, 38315, 38318.<br>- Coronary artery bypass graft, item 38497 – 38504.<br>- Selective coronary angiography, item 38215 – 38246	-	+ Digoxin: C01AA05<br>+ Isosorbide mononitrate: C01DA14<br>+ Glyceryl trinitrate: C01DA02	+ Ischemic heart disease: 410-414<br>+ Heart failure: 428	+ Ischemic heart disease: I20-I25<br>+ Heart failure: I50

**Stroke**	-	-	-	+ Cerebrovascular diseases: 430-438	+ Cerebrovascular diseases: I60-I69
**Hysterectomy***	MBS claims for hysterectomy:<br>Laparoscopic abdominal hysterectomy: 35751, 35753, 35754, 35756<br>Abdominal hysterectomy: 35653, 35661, 35668, 35669, 35671, 35729<br>Vaginal hysterectomy replay: 35657, 35673, 35750	Laparoscopic abdominal hysterectomy: 90448-00, 90448–01, 90448–02<br>Abdominal hysterectomy: 35653-00, 35653–01, 35653–04, 35661–00, 35670–00, 35667–00, 35664–00.<br>Vaginal hysterectomy: 35750-00, 35753-02, 35657–00, 35673–02, 35667–01, 35664–01.<br>Pelvic exenteration: 90450–00, 90450–01, 90450–02	-	Laparoscopic abdominal hysterectomy:<br>Abdominal hysterectomy: 68.3, 68.4, 68.6,<br>Vaginal hysterectomy: 68.5, 68.7<br>Pelvic exenteration: 68.8<br>Other and unspecified hysterectomy: 68.9	-
**Oophorectomy***	-	Laparoscopic partial oophorectomy: 35638-01	-	Unilateral: 65.3, 65.4<br>Bilateral: 65.5, 65.6	-

		Unilateral: 35638-02, 35713-07<br>Bilateral: 35638-03, 35717-01			
**Mastectomy***	MBS claims for<br>+ Total mastectomy: 31519 (unilateral), 31520 (bilateral).<br>+ Subcutaneous Mastectomy: 31524, 31522 (skin-sparing, unilateral), 31523 (skin-sparing, bilateral), 31528 (nipple-sparing, unilateral), 31529 (nipple-sparing, bilateral)	+ Simple mastectomy: 31518-00 (unilateral), 31518-01 (bilateral).<br>+ Subcutaneous mastectomy: 31524-00 (unilateral), 31524-01 (bilateral)	-	+ Unilateral: 85.33, 85.34, 85.41, 85.43, 85.45, 85.47<br>+ Bilateral: 85.35, 85.36, 85.42, 85.44, 85.46, 85.48 or any combination of 2 mastectomy codes<br>+ Prophylactic mastectomy - mostly bilateral mastectomy: V50.41	+ Unilateral: Z90.12, Z90.11<br>+ Bilateral: Z90.13
**Specialist/consulting physician attendances**	MBS claims for:<br>Other non-referred attendances (not GP): 52, 53, 54, 57, 58, 59, 60, 65, 92, 93, 95, 96<br>Specialist attendances: 99, 104, 105, 106, 107, 108, 109, 113<br>Consultant physician attendances: 110, 112, 114, 116, 119, 122, 128, 131, 132, 133<br>Prolonged attendances: 160, 161, 162, 163, 164<br>Urgent attendances after hours: 598, 600<br>Emergency physician attendance: 501, 503, 507, 511, 515, 519, 520, 530, 532, 534, 536<br>Consultant psychiatrist attendances: 288, 289, 291, 293, 296, 297, 299, 300, 302,	-	-	-	-

	304, 306, 308, 310, 312, 314, 316, 318, 319, 320, 322, 324, 326, 328, 330, 332, 334, 336, 338, 342, 344, 346, 348, 350, 352, 353, 355, 356, 357, 358, 359, 361, 364, 366, 367, 369, 370<br>Pain and palliative medicine: 2799, 2801, 2806, 2814, 2820, 2824, 2832, 2840, 2946, 2949, 2954, 2958, 2972, 2974, 2978, 2984, 2988, 2992, 2996, 3000, 3003, 3005, 3010, 3014, 3015, 3018, 3023, 3028, 3032, 3040, 3044, 3051, 3055, 3062, 3069, 3074, 3078, 3083, 3088, 3093<br>Geriatric medicine: 141, 143, 145, 147, 149				
**GP Attendances**	MBS claims for:<br>GP Health Assessment: 93470, 93479<br>GP Chronic Disease Management Plan: 229, 230, 231, 232, 233, 93469, 93475<br>GP Multidisciplinary Case Conference: 235, 236, 237, 238, 239, 240, 243, 244, 735, 739, 743, 747, 750, 758<br>Medication Management Review (domiciliary): 245, 900<br>Medication Management Review (residential): 249, 903.<br>GP Mental Health: 272, 276, 277, 279, 281, 282, 894, 896, 898, 941, 942, 2121, 2150, 2196, 90264, 90265, 92112, 92113, 92114, 92115, 92116, 92117, 92118, 92119, 92120, 92121, 92122, 92123, 92124, 92125, 92126, 92127, 92128, 92129, 92130, 92131, 92132, 92133, 92134, 92135, 92146, 92147, 92148, 92149, 92150, 92151, 92152, 92153, 92154, 92155, 92156, 92157, 92158, 92159, 92160, 92161, 92170, 92171, 92176, 92177, 92182, 92184, 92186, 92188, 92194, 92196, 92198, 92200	-	-	-	-

	After-hours GP: 597-600 (urgent); 5000, 5020, 5040, 5060, 5200, 5203, 5207, 5208, 5003, 5010, 5023, 5028, 5043, 5049, 5063, 5067, 5220, 5223, 5227, 5228, 5260, 5263, 5265, 5267, 733, 737, 741, 745, 761, 763, 766, 769, 772, 776, 788, 789 (non-urgent)<br>GP – Practice Incentive Program services: 251, 252, 253, 254, 255, 256, 257 (cervical smear); 259, 260, 261, 262, 263, 264 (diabetes mellitus annual cycle of care); 265, 266, 268, 269, 270, 271 (asthma cycle of care)<br>GP short (level A): 3, 4, 2095, 2461, 90020, 91790, 91795, 91890<br>GP Standard (Level B): 23, 24, 2144, 2463, 90035, 91800, 91809, 91891<br>GP Long (Level C): 36, 37, 2180, 2464, 90043, 91801, 91810, 91894<br>GP Prolonged (Level D): 44, 47, 2193, 2465, 90051, 91802, 91811<br>Other Non-referred Medical Practitioner attendances: 899, 901, 905, 906, 90002, 91792, 91794, 91797, 91799, 91803, 91804, 91805, 91806, 91807, 91808, 91812, 91813, 91814, 91815, 91816, 91817, 91892, 91895, 92716, 92717, 92719, 92720, 92722, 92723, 92725, 92726, 92732, 92733, 92735, 92736, 92738, 92739, 92741, 92742, 92747, 93660, 93661, 93681, 93682, 93684, 93685, 93691, 93692, 93694, 93695, 93701, 93702, 93704, 93705<br>GP Focussed Psychological Strategies and Family Group Therapy: 283, 285, 286, 287, 371, 372<br>GP Acupuncture: 173, 193, 195, 197, 199<br>GP Telehealth (patient-end support): 812, 827, 829, 867, 868, 869, 873, 876, 881, 885, 891, 892				

	GP attendances relating to residential aged care facilities: 232, 249, 731, 772, 776, 788, 789, 829, 869, 881, 892, 903, 2125, 2138, 2179, 2220, 5010, 5028, 5049, 5067, 5260, 5263, 5265, 5267, 92102, 92071, 92058, 92027				
**Invasive breast cancer**	-	-	-	+ Invasive breast cancer: 174.x	+ Invasive breast cancer: C50.xxx
**Non-invasive breast cancer**	-	-	-	+ Non-invasive breast cancer: 233.0	+ Non-invasive breast cancer: D05.0, D05.01, D05.02 (lobular carcinoma in situ), D05.10, D05.11, D05.12 (Intraductal carcinoma in situ), D05.80, D05.81, D05.82 (other specified type of carcinoma in situ, breast), D05.90, D05.91, D05.92 (unspecified type of carcinoma in situ of breast)
**Unspecified breast cancer**	-	-	-	+ Personal history of malignant neoplasm of breast: V10.3<br>+ Secondary malignant neoplasm of the breast (cancer metastatic to the breast): 198.81	+ Personal history of malignant neoplasm of breast: Z85.3<br>+ Secondary malignant neoplasm of the breast (cancer metastatic to the breast): C79.81

**Other cancers (excluding breast cancer and non-melanoma skin cancers)**	-	-	-	140 - 172, 175 - 208	C00–C49, C51-C97, D45, D46, D47.1, D47.3–D47.5
**Statins use**	-	-	+ HMG CoA reductase inhibitors: C10AA<br>+ Combinations of various lipid modifying agents: C10BA<br>+ Lipid modifying agents in combination with other drugs: C10BX	-	-
**Combined hormonal contraceptives**	-	-	+ Progestogens and estrogens, fixed combinations: G03AA<br>+ Progestogens and estrogens, sequential preparations: G03AB<br>+ Vaginal ring with progestogen and estrogen: G02BB01<br>+ Cyproterone and estrogen: G03HB01	-	-
**Combined menopausal hormone therapy**	-	-	+ Progestogens and estrogens in combinations: G03F<br>+ Vaginal ring with progestogen and estrogen: G02BB01<br>+ Cyproterone and estrogen: G03HB01<br>+ Combinations of ATC codes for estrogens only and progestogens only:	-	-

			Estrogens: G03C Progestogens: G03D, Progestogen intrauterine device: G02BA03		
**Comorbidity (Rx- Risk score)**					
Alcohol dependency	-	-	Disulfiram, Naltrexone: N07BB01 - N07BB99	-	-
Allergies	-	-	Antihistamines (except hydroxyzine and diphenhydramine), nasal anti-inflammatories: R01AC01 -R01AD60, R06AD02 - R06AX27, R06AB04	-	-
Anticoagulants	-	-	Anticoagulants: B01AA03 - B01AB06, B01AE07, B01AF01, B01AF02, B01AX05	-	-
Antiplatelets	-	-	Antiplatelets: B01AC04 - B01AC30	-	-
Anxiety	-	-	Anxiolytics (benzodiazepines): N05BA01-N05BA12, N05BE01	-	-
Arrhythmias	-	-	Antiarrhythmics, Digoxin: C01AA05, C01BA01 - C01BD01, C07AA07	-	-
Benign prostatic hypertrophy	-	-	Alpha blockers: G04CA01-G04CA99, G04CB01, G04CB02 for men only		

Bipolar disorder	-	-	Lithium: N05AN01	-	-
Chronic airways disease	-	-	Inhaled bronchodilators: R03AC02-R03DC03, R03DX05	-	-
Congestive heart failure	-	-	Loop diuretics AND, ACE or ARB; Betablockers selective for heart failure. Aldosterone antagonists specific for heart failure: C03DA02–C03DA99, C07AB02—if PBS item code is 8732N, 8733P, 8734Q, 8735R. C07AB07, C07AG02, C07AB12, C03DA04 (C03CA01–C03CC01 and C09AA01–C09AX99, C09CA01–C09CX99 †		
Dementia	-	-	Anticholinesterases or other dementia medicines: N06DA02-N06DA04, N06DX01	-	-
Depression	-	-	Antidepressants: N06AA01-N06AG02, N06AX03- N06AX11, N06AX13-N06AX18, N06AX21-N06AX26	-	-
Diabetes			Insulins, oral Hypoglycaemics: A10AA01–A10BX99		
Epilepsy	-	-	Anticonvulsants: N03AA01-N03AX99	-	-

Glaucoma	-	-	Topical antiglaucoma Agents: S01EA01-S01EB03, S01EC03-S01EX99	-	-
Gastric acid disorder	-	-	Histamine H2-receptor antagonists, Proton-pump inhibitor: A02BA01-A02BX05	-	-
Gout	-	-	Antigout agents: M04AA01-M04AC01	-	-
Hepatitis B	-	-	Anti Hep B antivirals: J05AF08, J05AF10, J05AF11	-	-
Hepatitis C	-	-	Interferon/ribavirin combinations, Protease inhibitors for hepatitis: J05AB54, J05AE11-J05AE12, J05AE14, J05AX14, J05AX15, J05AX65, J05AB04 L03AB10, L03AB11, L03AB60, L03AB61	-	-
HIV	-	-	Anti-HIV antivirals: J05AE01-J05AE10, J05AF01-J05AF07, J05AF09, J05AF12-J05AG05, J05AR01-J05AR99, J05AX07-J05AX09 J05AX12	-	-
Hyperkalaemia	-	-	Sodium polystyrene Sulfonate: V03AE01	-	-
Hyperlipidaemia	-	-	Antilipaemic agents: C10AA01-C10BX09, A10BH03‡		
Hypertension	-	-	Thiazides, potassium sparing agents,		

			combination antihypertensives, other antihypertensives: C03AA01–C03BA11, C03DB01- C03DB99, C03EA01, C09BA02–C09BA09, C09DA02–C09DA08, C02AB01–C02AC05, C02DB02–C02DB99 (C03CA01–C03CCO1 or C09CA01–C09CX99)§		
Hyperthyroidism	-	-	Antithyroid Preparations: H03BA02, H03BB01	-	-
Hypothyroidism	-	-	Thyroid replacements: H03AA01-H03AA02	-	-
Irritable bowel syndrome	-	-	IBS specific drugs, rectal anti-inflammatories: A07EC01–A07EC04, A07EA01–A07EA02, A07EA06, L04AA33	-	-
Ischemic heart disease: Angina	-	-	Nitrates: C01DA02-C01DA14, C01DX16, C08EX02		
Ischemic heart disease: Hypertension			C07AA01–C07AA06, C07AA08–C07AB01, C07AB02—if PBS item code is not 8732N, 8733P, 8734Q, 8735R, C07AG01, C08CA01–C08DB01, C09DB01–C09DB04, C09DX01, C09BB02–C09BB10,		

			C07AB03, C09DX03, C10BX03¶		
Incontinence	-	-	G04BD01–G04BD99		
Inflammation/pain	-	-	NSAIDS: M01AB01-M01AH06	-	-
Liver failure	-	-	Rifaximin: A06AD11, A07AA11	-	-
Malignancies	-	-	Antineoplastics agents (excluding topical): L01AA01-L01XX41	-	-
Malnutrition	-	-	Enteral nutritional Supplements: B05BA01-B05BA10	-	-
Migraine	-	-	Antimigraine Medications: N02CA01-N02CX01	-	-
Osteoporosis/Paget's	-	-	Alendronate, etidronate: M05BA01-M05BB05, M05BX03, M05BX04, G03XC01, H05AA02	-	-
Pain	-	-	Opiate containing Medications: N02AA01–N02AX02, N02AX06, N02AX52, N02BE51		
Pancreatic insufficiency	-	-	Pancreatic exocrine enzyme replacement: A09AA02	-	-
Parkinsons disease	-	-	Antiparkinson agents: N04AA01-N04BX02	-	-
Psoriasis	-	-	Systemic and topical antipsoriatics: D05AA01-D05AA99, D05BB01-D05BB02, D05AX02, D05AC01-D05AC51, D05AX52	-	-

Psychotic illness	-	-	Antipsychotics: N05AA01-N05AB02, N05AB06-N05AL07, N05AX07-N05AX13	-	-
Pulmonary hypertension	-	-	Antihypertensives for pulmonary arterial hypertension: C02KX01–C02KX05, PBS item code 9547L, 9605M	-	-
Renal disease	-	-	Alpha erythropoietin, calciferol, calcitriol, sevelamer: B03XA01-B03XA03, A11CC01-A11CC04, V03AE02, V03AE03, V03AE05	-	-
Smoking cessation	-	-	Nicotine, Bupropion: N07BA01-N07BA03, N06AX12	-	-
Steroid-responsive conditions	-	-	Glucocorticoids: H02AB01-H02AB10	-	-
Transplant	-	-	Immune suppressants: L04AA06, L04AA10, L04AA18, L04AD01, L04AD02	-	-
Tuberculosis	-	-	Anti-tubercular agents: J04AC01 - J04AC51, J04AM01 - J04AM99	-	-

MBS: Medicare Benefits Schedule, PBS: Pharmaceutical Benefits Scheme, AM: Australia Modification, ICD: International Classification of Diseases, FNA: Fine-needle aspiration, ACHI: The Australian Classification of Health Interventions. Notes: MBS codes and procedure codes are exclusively used for Australian data. *: Procedures conducted out of hospital or in private hospitals are recorded in MBS data by MBS item number whereas those conducted in public hospitals are captured in the hospital admission data by procedure codes. †: Must have at least two medicines prescribed with one of those medicines having an ATC code from C03CA01–C03CC01 and the other having an ATC code from either C09AA01–C09AX99 or C09CA01–C09CX99. ‡: Combination product for hyperlipidaemia and diabetes. §: Can have medicine dispensed with an ATC code C03CA01–C03CC01 or C09AA01–C09AX99, but not both, as this would indicate chronic heart failure. ¶: Combination product for hyperlipidaemia and ischaemic heart disease: hypertension
