# Supplementary material for: Association between long-term use of calcium channel blockers (CCB) and the risk of breast cancer: a retrospective longitudinal observational study protocol
Source: BMJ Open. 2024 Mar 8;14(3):e080982. doi: 10.1136/bmjopen-2023-080982 (PMC10928765; doi:10.1136/bmjopen-2023-080982)
Supplement: Supplementary data [file bmjopen-2023-080982supp003.pdf]

Title: Association between long-term use of calcium channel blockers (CCB) and the risk of breast cancer: A retrospective longitudinal observational study protocol

Appendix Table 3. Description and response categories of data items from baseline surveys of the ALSWH Study, the 45 and Up Study and Rotterdam Study

Table A. The Australian Longitudinal Study on Women's Health Study (ALSWH)

Description of data item	Question	Response categories
**Socio-demographic characteristics**		
Age	BOTH COHORTS: What is your date of birth?	Date variable (DD/MM/YYY)
Marital status	BOTH COHORTS: What is your present marital status?	+ 1946-51 COHORT: Married; de facto (in a relationship); widowed; separated; divorced; never married<br>+ 1921-26 COHORT: Married (registered); de facto relationship (opposite sex); de facto relationship (same sex); separated; divorced; widowed; never married
Country of birth	BOTH COHORTS: In which country were you born?	BOTH COHORTS: Australia; UK; Ireland; Italy; Greece; New Zealand; Vietnam; other (please specify)
	BOTH COHORTS: If you were not born here, when did you first arrive in Australia with the intention of living here for one year or more?	+ 1946-51 COHORT: 1955 or earlier; 1956-1965; 1966-1985; 1986-1990; 1991 or later<br>+ 1921-26 COHORT: 1935 or earlier; 1936-1945; 1946-1955; 1956-1965; 1966-1985; 1986-1990; 1991 or later
Education status	BOTH COHORTS: What is the highest qualification you have completed?	BOTH COHORTS: No formal qualifications; school or intermediate certificate (or equivalent); higher school or leaving certificate (or equivalent); trade/apprenticeship (e.g. hairdresser, chef); certificate/diploma (e.g. child care, technician); university degree; higher university degree (e.g. Grad Dip, Masters, PhD)
**Lifestyle characteristics**		
Body mass index (height/weight)	BOTH COHORTS: How tall are you without shoes?<br>BOTH COHORTS: How much do you weigh without clothes or shoes?	Continuous variable (height in cm)<br>Continuous variable (weight in kg)

Description of data item	Question	Response categories
Physical activity	BOTH COHORTS:<br>If you add up all the time you spent doing each activity LAST WEEK, how much time did you spend ALTOGETHER doing each type of activity?<br>1. Walking briskly (for recreation or exercise or to get to or from places)<br>2. Moderate leisure activity (like social tennis, moderate exercise classes, recreational swimming, dancing)<br>3. Vigorous leisure activity (that make you breathe harder or puff and pant like aerobics, competitive sport, vigorous cycling, running, swimming)<br>3. Vigorous household or garden chores (that make you breathe harder or puff and pant)	Continuous variable (time in hours/minutes)
Smoking status	1946-51 COHORT: How often do you currently smoke cigarettes or any tobacco products?	1946-51 COHORT: Daily; at least weekly (but not daily); less often than weekly; not at all
Alcohol drinks per week	1946-51 COHORT: How often do you usually drink alcohol?	1946-51 COHORT: I never drink alcohol; I drink rarely; less than once a week; on 1 or 2 days a week, on 3 or 4 days a week; on 5 or 6 days a week; every day
	1946-51 COHORT: On a day, when you drink alcohol, how many drinks do you usually have	1946-51 COHORT: 1 or 2 drinks per day; 3 or 4 drinks per day; 5 to 8 drinks per day; 9 or more drinks per day
Shift work at night	1946-51 COHORT: Do you normally do paid work at night?	1946-51 COHORT: Yes; no
**Clinical characteristics**		

Description of data item	Question	Response categories
High BP	BOTH COHORTS: Have you ever been told by a doctor that you have hypertension (high blood pressure)?	BOTH COHORTS: Yes; no
	BOTH COHORTS: In the past 3 years, have you been diagnosed or treated for high blood pressure?	BOTH COHORTS: Yes; no
Diabetes	BOTH COHORTS: Have you ever been told by a doctor that you have diabetes?	BOTH COHORTS: Yes; no
	BOTH COHORTS: In the past 3 years, have you been diagnosed or treated for diabetes (high blood sugar)?	BOTH COHORTS: Yes; no
	1946-51 COHORT: Have you ever been told by a doctor that you have non-insulin dependent (type 2) diabetes?	1946-51 COHORT: Yes; no
	1946-51 COHORT: In the past 3 years, have you been diagnosed or treated for non-insulin dependent (type 2) diabetes?	1946-51 COHORT: Yes; no
	1921-26 COHORT: In the last 3 years, have you been told by a doctor that you have diabetes?	1921-26 COHORT: Yes; no
Heart disease	BOTH COHORTs: Have you ever been told by a doctor that you have heart disease?	BOTH COHORTs: Yes; no
	1946-51 COHORT: In the past 3 years, have you been diagnosed or treated for heart disease?	1946-51 COHORT: Yes; no
	1921-26 COHORT: In the past 3 years, have you been diagnosed or treated for angina/heart attack/other heart problems?	1921-26 COHORT: Yes; no
Stroke	BOTH COHORTS: Have you ever been told by a doctor that you have stroke?	BOTH COHORTS: Yes; no

Description of data item	Question	Response categories
	BOTH COHORTS: In the past 3 years, have you been diagnosed or treated for stroke?	BOTH COHORTS: Yes; no
	1921-26 COHORT: In the last 3 years, have you been told by a doctor that you have stroke	1921-26 COHORT: Yes; no
**Reproductive health**		
Age at menarche	1946-51 COHORT: At what age did your period start?	Continuous variable (Age in years)
Age at menopause	1946-51 COHORT: Have you had a period or menstrual bleeding in the last 12 months?	1946-51 COHORT: Yes; no
	1946-51 COHORT: If you have reached menopause, at what age did your periods completely stop?	Continuous variable (Age in years)
Age when had first child	1946-51 COHORT: If you have given birth, please write the year of the birth in the birth (if you had twins, please write the date twice)	Continuous variable (Year)
Number of children	1946-51 COHORT: If you have given birth, please write the year of the birth in the birth (if you had twins, please write the date twice)	Continuous variable (Year)
History of hysterectomy/ both ovaries removed	BOTH COHORTS: Have you ever had hysterectomy?	BOTH COHORTS: Yes; no
	BOTH COHORTS: In the PAST THREE YEARS, have you had hysterectomy?	BOTH COHORTS: Yes; no
	BOTH COHORTS: Have you ever had both ovaries removed?	BOTH COHORTS: Yes; no
	BOTH COHORTS: In the PAST THREE YEARS, have you had both ovaries removed?	BOTH COHORTS: Yes; no

Description of data item	Question	Response categories
**Healthcare utilisation**		
Concession card holders	1946-51 COHORT: Do you have a Health Care Card? (This is a card that entitles you to discounts and assistance with medical expenses. This is not the same as a Medicare card)	1946-51 COHORT: Yes; no
Mammogram	1946-51 COHORT: When did you last have a mammogram?	1946-51 COHORT: I have never had a mammogram; 2 years ago or less; more than 2 but less than 5 years ago; 5 or more years ago; not sure
	1946-51 COHORT: Have you ever had an abnormal mammogram?	1946-51 COHORT: No; Yes in the last 5 years; Yes more than 5 years ago
	1946-51 COHORT: In the last two years, have you had a mammogram?	1946-51 COHORT: Yes; no
**Diagnosis of cancer**		
A diagnosis/history of breast cancer	BOTH COHORT: Have you ever been told by a doctor that you have breast cancer?	BOTH COHORT: Yes; no
	1946-51 COHORT: In the past 3 years, have you been diagnosed or treated for breast cancer?	1946-51 COHORT: Yes; no
	1921-26 COHORT: In the last 3 years, have you been told by a doctor that you have breast cancer?	1921-26 COHORT: Yes; no
**Medicine use**		
Current use of hormonal contraception	1946-51 COHORT: Are you currently taking the oral contraceptive pill for any reason?	1946-51 COHORT: Yes; no
Current use of menopausal hormone therapy	1946-51 COHORT: Are you currently on hormone replacement therapy (HRT)?	1946-51 COHORT: Yes; no

BP: blood pressure

Table B. The 45 and Up Study

Description of data item	Question	Response categories
**Socio-demographic characteristics**		
Age	What is your date of birth?<br>What is today's date?	Date variable (DD/MM/YYYY)
Marital status	What best describes your current situation?	Single; married; de facto/living with a partner; widowed; divorced; separated
Country of birth	In which country were you born?	Australia; UK; Ireland; Italy; China; Greece; New Zealand; Germany; Lebanon; Philippines; Netherlands; Vietnam; Malta; Poland; other (please specify)
	What year did you first come to live in Australia for one year or more?	Continuous variable (Year)
Work status	What is your current work status? (you can cross more than one box)	In full time paid work; self-employed; in part time paid work; doing unpaid work; completely retired/pensioner; studying; partially retired; looking after home/family; disabled/sick; unemployed; other
	If you are partially or completely retired, how old were you when you retired?	Continuous variable (Age in years)
	Why did you retire?	Reached usual retirement age; lifestyle reasons; to care for family member/friend; ill health; made redundant; could not find a job; other
Education status	What is the highest qualification you have completed?	No school certificate or other qualifications; school or intermediate certificate (or equivalent); higher school or leaving certificate or equivalent); trade/apprenticeship (e.g. hairdresser, chef); certificate/diploma (e.g. child care, technician); university degree or higher
**Lifestyle characteristics**		
Body mass index (height/weight)	How tall are you without shoes?	Continuous variable (Centimetres, Feet, Inches)
	About how much do you weigh?	Continuous variable (Kilograms; Stone; Pounds)
Physical activity	If you add up all the time you spent doing each activity LAST WEEK, how much time	Walking continuously; Vigorous physical activity; Moderate physical activity

Description of data item	Question	Response categories
	did you spend ALTOGETHER doing each type of activity?	
Smoking status	Have you ever been a regular smoker?	Yes; no
	Are you a regular smoker now?	Yes; no
Alcohol drinks per week	About how many alcoholic drinks do you have each week?<br>On how many days each week do you usually drink alcohol?	Continuous variable (Integer)
**Clinical characteristics**		
High BP/ Diabetes/ Heart Disease/ Stroke/ Breast cancer/ Other cancer	Has a doctor EVER told you that you have:	Skin cancer (not melanoma); Melanoma; Breast cancer (F); Prostate cancer (M); Other cancer (free text); Heart disease (free text); High blood pressure - when pregnant (F); High blood pressure - when not pregnant (F); High blood pressure (M); Stroke; Diabetes; Blood clot (thrombosis); Enlarged prostate (M); Asthma; Hay fever; Depression; Anxiety; Parkinson's disease; None of these
Family history of heart disease/ stroke/ breast cancer/ ovarian cancer	Have your mother, father, brother(s) or sister(s) ever had:	Heart disease; High blood pressure; Stroke; Diabetes; Dementia/Alzheimer's; Parkinson's disease; Severe depression; Severe arthritis; Breast cancer; Bowel cancer; Lung; Melanoma; Prostate cancer; Ovarian cancer; Osteoporosis; Hip fracture; Do not know
**Reproductive health**		
Age at menopause	Have you been through menopause?	No; not sure (because hysterectomy, taking HRT, etc.); my periods have become irregular; yes
	If yes, how old were you when you went through menopause?	Continuous variable (Age in years)
Age when had first child	How old were you when you gave birth to your FIRST child?	Continuous variable (Age in years)
Number of children	How many children have you given birth to?	Continuous variable (Age in years)
History of hysterectomy/ both ovaries removed	Have you ever had hysterectomy?	Yes; no
	Have you ever had both ovaries removed?	Yes; no

Description of data item	Question	Response categories
Breastfeeding	For how many months, in total, have you breastfed?	Continuous variable (number of months)
**Healthcare utilisation**		
Concession card holders	Which of the following do you have? (excluding Medicare)	Private health insurance-with extra; private health insurance – without extras; Department of Veterans' Affairs white or gold card; Health care concession card; none of these.
Mammogram	Have you ever been for a breast screening mammogram?	Yes; no
	If Yes, what year did you have your last mammogram?	Continuous variable (Year)
	How many times have you been for breast screening altogether?	Continuous variable (Integer)
**Medicine use**		
Current use of hormonal contraception	Have you ever used the pill or other hormonal contraceptives? (e.g. the combined pill, mini pill, contraceptive implant or injections)	Yes; no
	If Yes, how old were you when you LAST used hormonal contraceptives? (please write your current age if you are still using them)	Continuous variable (Age in years)
Current use of menopausal hormone therapy	Have you ever used hormone replacement therapy (HRT)?	Yes; no
	Are you currently taking HRT?	Yes; no

F: female, M: male, BP: blood pressure, HRT: hormone replacement therapy

Table C. Rotterdam Study

Description of data item	Question	Response categories
**Socio-demographic characteristics**		
Age	Date of birth was provided by the municipality, and age was subsequently computed by data management	Continuous variable (Age in years)
Marital status	*Home interview:*<br>What is your marital status?	Never been married; married (once); widowed, then remarried; divorced and remarried; never been married, lives with unmarried spouse; widowed, lives with unmarried spouse; divorced, lives with unmarried spouse; widowed; divorced
Work status	*Home interview:*<br>What is your current employment status?	Employed; unemployed, out of job; house wife/man; unfit for work; rentier; early retirement; retirement
Education status	*Home interview:*<br>What is your highest attained education?	Primary education; lower vocational education; intermediate secondary education; intermediate vocational education; general secondary education; higher education (Higher professional education); higher education (University)
**Lifestyle characteristics**		
Body mass index (height/weight)	*Clinical examination:*<br>Weight was assessed with the participant wearing light indoor clothes, without shoes. Height was measured.	Weight in kilograms, height in centimetres
Smoking status	*Home interview:*<br>Do you smoke cigarettes?	No; yes; don't know; no answer
	Did you quit smoking ever?	No; yes; don't know; not applicable; no answer
	Did you use to smoke cigarettes earlier?	No; yes; don't know; not applicable; no answer
	Did you smoke cigars? Did you ever smoke cigars or did you smoke cigars but quitted now?	No, never; ever little cigars; ever big cigars; ever both; yes little; yes big; yes both; don't know; no answer
Alcohol drinks per week	How many glasses of beer/wine/spirit do you drink a day?	Continuous variable (Integer)

Description of data item	Question	Response categories
**Clinical characteristics**		
High BP	Clinical exam: Hypertension	No; Yes;
Diabetes	Clinical exam: Diabetes	No; Yes;
Systolic BP	Clinical exam: Systolic BP	Continuous variable
Diastolic BP	Clinical exam: Diastolic BP	Continuous variable
Blood cholesterol level	Clinical exam: Blood drawn, clinical lab.	Continuous variable (Value)
Family history of heart disease	*Home interview:* Did one of your parents or siblings experience a heart attack?	No; yes
**Reproductive health**		
Age at menarche	*Home interview:* At what age did your period start?	Continuous variable (Age in years)
Age at menopause	*Home interview:* At what age had your periods stopped definitively?	Continuous variable (Age in years)
Age when had first child	*Home interview:* In what year was your first child born?	Continuous variable (Age in years, calculated by data management team)
Number of children	*Home interview:* How many pregnancies (of at least 6 months) have you had?	Continuous variable (Integer)
History of hysterectomy	*Home interview:* Tick box, yes no	No; Yes
History of both ovaries removed	*Home interview:* Tick box, yes no	No; Yes

BP: blood pressure. Clinical exams were undertaken via automated coupling of the study database with medical records from general practitioners.
