# Supplementary material for: Association between long-term use of calcium channel blockers (CCB) and the risk of breast cancer: a retrospective longitudinal observational study protocol
Source: BMJ Open. 2024 Mar 8;14(3):e080982. doi: 10.1136/bmjopen-2023-080982 (PMC10928765; doi:10.1136/bmjopen-2023-080982)
Supplement: Author's manuscript [file bmjopen-2023-080982.draft_revisions.pdf]

BMJ Open

BMJ Open is committed to open peer review. As part of this commitment we make the peer review history of every article we publish publicly available.

When an article is published we post the peer reviewers' comments and the authors' responses online. We also post the versions of the paper that were used during peer review. These are the versions that the peer review comments apply to.

The versions of the paper that follow are the versions that were submitted during the peer review process. They are not the versions of record or the final published versions. They should not be cited or distributed as the published version of this manuscript.

BMJ Open is an open access journal and the full, final, typeset and author-corrected version of record of the manuscript is available on our site with no access controls, subscription charges or pay-per-view fees (http://bmjopen.bmj.com).

If you have any questions on BMJ Open's open peer review process please email info.bmjopen@bmj.com

BMJ Open

Association between long-term use of calcium channel blockers (CCB) and the risk of breast cancer: A retrospective longitudinal observational study protocol

Journal:	*BMJ Open*
Manuscript ID	bmjopen-2023-080982
Article Type:	Protocol
Date Submitted by the Author:	16-Oct-2023
Complete List of Authors:	Ho, Chau; Curtin University, School of Population Health, Faculty of Health Sciences<br>Ha, Thi Ninh; Curtin University, School of Population Health, Faculty of Health Sciences<br>Youens, David; Curtin University, School of Population Health, Faculty of Health Sciences; The University of Western Australia, Cardiovascular Epidemiology Research Centre, School of Population and Global Health<br>Abhayaratna, Walter P.; Canberra health services; Australian National University, School of Medicine and Psychology<br>Bulsara, Max; The University of Notre Dame Australia, Institute for Health Research<br>Hughes, Jeffery; Curtin University, Curtin Medical School; PainChek Ltd<br>Mishra, Gita; The University of Queensland, School of Public Health<br>Pearson, Sallie-Anne; University of New South Wales, Medicines Policy Research Unit; The NNHMRC Medicines Intelligence Centre of Research Excellence<br>Saunders, Christobel M; The University of Western Australia, Medical School; The University of Melbourne, Melbourne Medical School<br>van Rooij, Frank J.A; Erasmus MC – University Medical Center, Department of Epidemiology<br>Wright, Cameron; Curtin University, School of Population Health, Faculty of Health Sciences; The University of Western Australia, Medical School<br>Preen, David; University of Western Australia, School of Population and Global Health; The NHMRC Medicines Intelligence Centre of Research Excellence<br>Reid, Christopher; Curtin University, School of Population Health, Faculty of Health Sciences<br>Ruiter, Rikje; Erasmus MC – University Medical Center, Department of Epidemiology; Maasstad Hospital, Department of Internal Medicine<br>Stricker, Bruno; Erasmus MC – University Medical Center, Department of Epidemiology<br>Moorin, Rachael; Curtin University, School of Population Health, Faculty of Health Sciences; University of Western Australia, School of Population and Global Health
Keywords:	Breast tumours < ONCOLOGY, Hypertension < CARDIOLOGY, EPIDEMIOLOGY

Title: Association between long-term use of calcium channel blockers (CCB) and the risk of breast cancer: A retrospective longitudinal observational study protocol

Authors: Chau LB Ho[1], Ninh Thi Ha[1], David Youens[1,2], Walter P. Abhayaratna[3,4], Max K. Bulsara[5], Jeff Hughes[6,7], Gita D Mishra[8], Sallie-Anne Pearson[9, 10], David B Preen[10,11], Christopher M Reid[1], Rikje Ruiter[12, 13], Christobel M Saunders[14,15,16], Bruno H Stricker[12], Frank J.A van Rooij[12], Cameron Wright[1, 14,17,18,] Rachael Moorin[1,11]

Affiliations:

[1]School of Population Health, Faculty of Health Sciences, Curtin University, WA, Australia, [2] Cardiovascular Epidemiology Research Centre, School of Population and Global Health, The University of Western Australia, WA, Australia, [3] Canberra Health Services, ACT, Australia, [4] School of Medicine and Psychology, Australian National University, ACT, Australia, [5] Institute for Health Research, The University of Notre Dame Australia, WA, Australia, [6]Curtin Medical School, Curtin University, WA, Australia, [7] PainChek Ltd, Sydney, NSW, Australia, [8] School of Public Health, The University of Queensland, Brisbane, QLD, Australia, [9] School of Population Health, University of New South Wales, Sydney, Australia, [10] The NHMRC Medicines Intelligence Centre of Research Excellence, [11] School of Population and Global Health, The University of Western Australia, WA, Australia, [12] Department of Epidemiology, Erasmus MC – University Medical Center, Rotterdam, the Netherlands, [13] Department of Internal Medicine, Maasstad Hospital, Rotterdam, the Netherlands, [14] Medical School, The University of Western Australia, WA, Australia, [15] Melbourne Medical School, The University of Melbourne, Victoria, Australia, [16] The Royal Melbourne Hospital, Victoria, Australia, [17] Fiona Stanley Hospital, WA, Australia, [18] School of Medicine, University of Tasmania, TAS, Australia

Corresponding author: David Youens. School of Population Health, Faculty of Health Sciences, Curtin University, Kent St, Bentley WA 6102, Australia. david.youens@curtin.edu.au.

For peer review only

For peer review only - http://bmjopen.bmj.com/site/about/guidelines.xhtml

Abstract

Introduction: Calcium channel blockers (CCB), a commonly prescribed antihypertensive (AHT) medicine, may be associated with increased risk of breast cancer. The proposed study aims to examine whether long-term CCB use is associated with the development of breast cancer and to characterise the dose-response nature of any identified association, to inform future hypertension management.

Methods and analysis: The study will use data from two of Australia's largest cohort studies; the Australian Longitudinal Study on Women's Health, and the 45 and Up Study, combined with the Rotterdam Study. Eligible women will be those with diagnosed hypertension, no history of breast cancer and no prior CCB use at start of follow-up (2004 to 2009). Cumulative dose-duration exposure to CCB and other AHT medicines will be captured at the earliest date of: the outcome (a diagnosis of invasive breast cancer); a competing risk event (e.g., bilateral mastectomy without a diagnosis of breast cancer, death prior to any diagnosis of breast cancer) or end of follow-up (censoring event). Fine and Gray competing risks regression will be used to assess the association between CCB use and development of breast cancer using a generalised propensity score to adjust for baseline covariates. Time-varying covariates related to interaction with health services will also be included in the model. Data will be harmonised across cohorts to achieve identical protocols and a two-step random effects individual patient-level meta-analysis will be used.

Ethics and dissemination: Ethical approval was obtained from the following Human research Ethics Committees: Curtin University (HRECs) (ref No. HRE2022-0335), NSW Population and Health Services Research Ethics Committee (2022/ETH01392 / 2022.31), ACT Research Ethics and Governance Office approval under National Mutual Acceptance for multi-jurisdictional data linkage research (2022.STE.00208). Results of the proposed study will be published in high-impact journals and presented at key scientific meetings.

Study Registration: ClinicalTrials.gov Identifier NCT05972785.

For peer review only

For peer review only - http://bmjopen.bmj.com/site/about/guidelines.xhtml

Strengths and limitations of this study

- The novel dose-duration methods used in this study will be a significant innovation to pharmaco-epidemiological analyses.
- This study will characterise any dose-response nature of the association between CCB use and breast cancer. If there is a positive association this would mean that CCBs would be avoided in women, a fundamental change to current practice. No association would provide reassurance regarding current clinical guidelines.
- Due to the nature of the observational study design, confounding by indication will be unavoidable. To minimise the impact of this issue, the study will be limited to participants with diagnosed/self-reported hypertension and comparisons will include participants exposed to other prescribed (non-CCB) AHT medicines in addition to those who have no pharmacotherapy for hypertension.
- Complete ascertainment of prior exposure will not be possible since we will not have lifetime capture of prescribing data, therefore CCB exposure may be under-reported.
- The diagnostic interval of breast cancer may vary between Australian and Dutch women, particularly since case ascertainment through cancer registries can take months from the initial diagnosis to coding notifications.

For peer review only

For peer review only - http://bmjopen.bmj.com/site/about/guidelines.xhtml

Introduction

Breast cancer has overtaken lung cancer as the most common cancer and the leading cause of death in women globally, accounting for two million new cases and 685,000 deaths worldwide in 2020 [1]. By 2040, breast cancer is expected to increase by 40% to more than three million new cases and one million deaths annually [2]. Breast cancer places a significant burden of disease, excess mortality and reduced quality of life on women and the broader health system, and identifying and mitigating potentially modifiable risk factors is a critical strategy in reducing this burden.

Hypertension is a well-established, modifiable risk factor for stroke and other cardiovascular events and a major contributor to chronic diseases such as heart failure, chronic kidney disease, and retinopathy [3]. Worldwide, hypertension causes more deaths and morbidity than any other biomedical risk factor [4]. Cardiovascular disease is responsible for approximately 17 million deaths a year, nearly one-third of total mortality globally [5]. Together with three other AHT medicines (i.e., angiotensin-converting enzyme inhibitors (ACEI), angiotensin receptor blockers (ARB) and thiazide or thiazide-like diuretics), dihydropyridine CCBs have been recommended for initiation as first line therapy in many countries [6, 7].

It has been hypothesised that CCBs may increase the risk of cancer by changing intracellular calcium levels, thereby affecting the process of apoptosis (programmed cell death), disabling the destruction of mutated cells and leading to development of diseases such as cancer in which there is indiscriminate replication of a mutated cell [8]. Calcium is known to play a regulatory role in apoptosis via multiple biochemical / pharmacological pathways [9-11]. It has been postulated that, due to its secretory nature, breast tissue may be more vulnerable to alterations in apoptotic activity than other tissue, leading to complex hormone-related relationships between apoptosis and breast carcinoma [12]. In addition, one particular CCB, nifedipine, has been found to increase proliferation and migration of breast cancer cells in vitro [13] and to reduce resting calcium concentration and apoptotic gene expression in mice [14].

In 2017, we published a systematic review and meta-analysis [15] highlighting the uncertainty regarding the association between long-term use of CCBs and breast cancer, primarily due to the limited availability of long-term follow-up data. This review and meta-analysis found that the literature is equivocal on whether CCBs are a risk factor for breast cancer. Li et al. [16] reported that long-term

For peer review only - http://bmjopen.bmj.com/site/about/guidelines.xhtml

(>10 years) use of CCBs was associated with a 2.4-fold (95% confidence intervals [CI], 1.2-4.9) increased risk of invasive ductal breast cancer and 2.6-fold (95% CI, 1.3-5.3) increased risk of invasive lobular breast cancer. In contrast, Grimaldi-Bensouda et al.[17], using data from the United Kingdom, and Wilson et al. [18], using data from the United States (US), from sisters with and without breast cancer failed to find an association between CCB use and breast cancer (odds ratio 0.95 (95% CI: 0.87-1.04) and hazard ratio 0.88 (95% CI 0.58-1.33), respectively). A non-significant association was found in US women aged 55+ years for CCB use but a protective effect for ACEI use [19]. However, a study by Gómez-Acebo et al. [20] using Spanish data found CCB use for five or more years was associated with a 1.77-times increased risk (95% CI, 0.99-3.17) of breast cancer. A 2021 systematic review of clinical trials showed no strong effect of any type of AHT medicine on breast cancer with a median follow-up of 4.2 years. However, a systematic review of cohort studies with longer follow-up (≥10 years) published in the same year, found the use of beta blockers, CCBs and diuretics were associated with increased breast cancer risk [21]. The authors noted the heterogeneity of study design, study duration and capture of drug exposure status and also that most studies were from Europe or North America, limiting external validity.

Recommendations for CCB use are different across countries. Calcium Channel Blockers are currently the second most commonly prescribed AHT medicine and are considered first-line treatment for hypertension in Australia [22]. In contrast, CCBs are not recommended for first-line therapy in the Netherlands, where thiazide diuretics are more commonly initially prescribed for blood pressure control [23] and this represents an opportunity to investigate the role of prescribing practices in cancer risk. Variations in prescribing patterns may drive the differences in the associations observed in studies of CCB use and risk of breast cancer across populations in addition to differences in underlying cancer risk due to varying environmental or biological/genetic factors (e.g., body mass index [BMI], physical activity, smoking and alcohol history, hormonal medicine use or mammographic density). We expect this to be true for CCB prescribing in Australia compared with The Netherlands. The similarities and differences between the Dutch and Australian cohorts have been leveraged previously in studies of medicine safety [24]. We therefore propose to use data from these two countries to: (i) explore

For peer review only

confounding and (ii) effect modification by these specific factors in the relationship between CCB use and breast cancer risk.

Long-term evaluation of medicine safety is best monitored via longitudinal pharmacovigilance and observational studies, as they have both linked claims data and good confounder adjustment obtained through surveys of participants whereas randomised clinical trials with sufficient follow-up are largely not feasible due to time and cost factors. Thus, we aim to examine whether long-term CCB use is associated with the development of breast cancer amongst women enrolled in three longitudinal cohort studies in Australia and The Netherlands and to characterise the dose-response nature of any such association. A secondary aim is to assess whether any differences in the association between CCB use and the development of breast cancer exist between Australian and Dutch women and, if so, to explore confounding and effect modification for a range of genetic, socio-economic and clinical factors.

Methods and analysis

Study design

This will be a retrospective, observational study conducted across three internationally renowned longitudinal cohorts incorporating both self-report and administrative data linked at the person-level. For the primary aim, the study will use a distributed methodological approach, which has been shown to assure methodological quality and transparency and increase the likelihood that differences between results are due to true variation in effect size, rather than design or process-related problems [25]. Our analysis will: (i) first use identical protocols across cohorts (harmonised analyses) and then (ii) use variable/cohort-specific protocols to allow the influence of cohort-specific variables to be investigated (non-harmonised analyses).

Data sources

We will utilise data from the Australian Longitudinal Study on Women's Health (ALSWH) [26], the New South Wales (NSW) Sax Institute's 45 and Up Study (45 and Up Study) [27] and the Rotterdam Study [28].

- The ALSWH [26] is a national study that began in 1996 with a random sample of more than 40,000 women in three birth cohorts. The surveys were conducted every three years since 1996, however, the 1921–26 cohort was surveyed 6 monthly from Nov 2011 for health and lifestyle

For peer review only - http://bmjopen.bmj.com/site/about/guidelines.xhtml

changes. In our study, we will use two longitudinal cohorts, the 1946–51 cohort (aged 45–50 years at recruitment) and the 1921–26 cohort (aged 70–75 years at recruitment).

- Administrative data for the ALSWH are included for all Australian states and territories, linked using probabilistic linkage with clerical review by the data linkage units in each state. Linked data used for this study include hospital admissions, emergency department presentations, cancer registry and the national death index data. Hospitalisation and cancer registry data are recorded separately in each Australian state; hence the start and end of these datasets vary according to the availability of the data in each state. Medicare Benefits Schedule (MBS) claims and Pharmaceutical Benefits Scheme (PBS) data were provided by Services Australia.
- The 45 and Up Study [27] is a longitudinal cohort of 267,357 participants aged ≥45 years residing in NSW, Australia at recruitment randomly sampled from the Services Australia Medicare enrolment database. Participants from rural and remote areas and those 80+ years of age were oversampled. Participants were recruited from 2005-2009 (2005 being a pilot study) and followed up every five years for changes in health and lifestyle. The response fraction was approximately 19%. Only female participants (53.6% of the cohort) recruited in the main study between 2006 and 2009 will be included in the proposed study.
- Survey data for the 45 and Up Study were linked to the NSW Admitted Patient Data Collection and the Australian Capital Territory (ACT) Admitted Patient Collection, the NSW and ACT Emergency Department Data Collection, the NSW and ACT Cancer Registry, the NSW and ACT Registry of Births Deaths & Marriages death registrations, the Australian Coordinating Registry Cause of Death Unit Record File, NSW and ACT BreastScreen and the NSW Pap Test Registry by the Centre for Health Record Linkage (CHeReL) using a probabilistic linkage procedure with clerical review. The current estimated rate of false-positive linkages is 0.5% (www.cherel.org.au). Linkage of 45 and Up Study cohort data to Medicare claims and PBS data provided by Services Australia was facilitated by the Sax Institute using a unique identifier and deterministic matching.
- The Rotterdam Study [28] is a prospective cohort study of persons aged ≥45 years living in the city of Rotterdam, The Netherlands. The study started with a pilot phase in the second half

For peer review only

of 1989, called “RS-I”. A second cohort was recruited in 2000 (RS-II) and a third cohort in 2006 (RS-III). By the end of 2008, the Rotterdam Study comprised 14,926 participants aged 45 years or over. At baseline, participants were interviewed and had a clinical examination that was repeated every 3 to 4 years. Follow-up of participants between clinical examinations is undertaken via automated coupling of the study database with medical records from general practitioners (GPs), who receive all relevant medical information from all caregivers of their patients. The data include hospital and specialist discharge summaries providing dates of diagnoses and major surgical interventions. Only female participants of the three longitudinal cohorts will be included in our study.

- The three Rotterdam longitudinal cohorts are linked at the individual level to records for dispensed medicines from all pharmacies in the study area (1991-2018), the Netherlands Comprehensive Cancer Organisation (1989-2016) and municipality (death) records (1989-2023).

A summary of the source of the major categories of data available for the study is provided in Table 1.

Study population

The same study population will be used for the primary and secondary aims. Entry to our study will be on survey completion date for women who participated in the ALSWH survey waves between 2004 and 2008 or the 45 and Up Study baseline survey between 2006 and 2009 or the date of the Rotterdam Study interview and clinical examinations for women who completed these between 2004 and 2008. Inclusion criteria will be women who at entry to our study (i) were alive and consented to linkage of administrative data, (ii) have evidence of hypertension from either self-reported hypertension (Australian longitudinal cohorts) or have a recorded diagnosis of hypertension in clinical examination data (Rotterdam cohorts). Women will be excluded at entry if they had (i) history of primary invasive/in situ breast cancer or cancer that is metastatic to the breast; or (ii) history of bilateral mastectomy; or (iii) evidence of CCB use prior to entry. Further exclusion criteria will be applied in Australian longitudinal cohorts only as per standard practice when using PBS data: (i) women with any evidence of being Department of Veterans’ Affairs (DVA) concession card holders, because DVA-subsidised prescriptions are funded separately and not captured in the PBS data held; or (ii) women who did not have evidence of concessional healthcare status

For peer review only

For peer review only - http://bmjopen.bmj.com/site/about/guidelines.xhtml

for more than one year during period from 2003 to 2012. Those with concessional status, e.g., those receiving the aged care pension or certain other Government benefits, have lower co-payments for PBS-listed medicines in comparison to "general beneficiaries". This exclusion is necessary because some AHT medicines fall below the PBS co-payment threshold for general beneficiaries, hence attracted no government reimbursement and were not captured in PBS data prior to an administrative change in 2012 [29]. In contrast the lower co-payment threshold for concessional patients means that AHT medicines can be captured throughout the study period.

Participants will be followed until the earliest date of one of the following: the outcome (a diagnosis of invasive breast cancer); a competing risk event (evidence of bilateral mastectomy without a diagnosis of breast cancer, death prior to any diagnosis of breast cancer) or end of the study follow-up (censoring event), whichever came first. Note that since the duration of the available data differs across the longitudinal cohorts, end of study follow-up will be established for each person based on the data available. Figure 1 presents a schematic diagram of timeline for entry, exposure and study exit (i.e., recording of the outcome, censoring, competing event or end of follow-up) of our study population.

As administrative data are available prior to entry, this allows for a "lookback" period to ascertain some aspects of health service use prior to study entry, for comorbidity ascertainment and to remove those with a history of CCB use or breast cancer diagnosis prior to time-zero for follow-up.

Harmonisation of data

Harmonisation of data will be undertaken using the six-step process described by Rolland et al. [30] and Fortier et al. [31] After obtaining approvals from all data access and ethics committees, the Australian data will be housed in cohort-specific folders in the Sax Institute's Secure Unified Research Environment (SURE) (https://www.saxinstitute.org.au/solutions/sure/), whereas the Rotterdam data will be uploaded and analysed in the Accessible Network Digital Research Environment Alliance (AnDREa) (www.andrea-cloud.eu). Related documents such as data processing scripts, and comments on specific decisions taken throughout the process will be recorded. An associated data dictionary will also be developed. To ensure content equivalence, each variable will be checked on (1) the definition used in the questionnaire, format, categories, unit, and time frame, (2) measurement method (e.g., self-reported, clinical examination, etc.), and (3) harmonisation rules. Variables relating to the main

For peer review only

exposure, outcomes/competing risk, socio-demographic, lifestyle, clinical, reproductive characteristics and healthcare utilisation that are harmonisable, together with those available for the cohort-specific analyses, are presented in Table 2. Where a variable has different categories across cohorts we will collapse categories to generate the closest possible match.

Exposure ascertainment

The exposure in this study, for both the primary and secondary aims, will be CCB dispensing including both combination and monotherapy products. Exploring the impact of confounding by indication for non-CCB AHT medicines is important because a modest increased risk of cancer in individuals with hypertension has been documented [32]. To avoid this issue, previous studies have used a comparator group of patients exposed to other AHTs and/or with a hypertension diagnosis [33]. Our study population is defined by the presence of hypertension since comparing only to a reference AHT medicine group risks biasing toward the null, if the comparator drug is associated with the outcome. Thus, to reduce bias to the null, women who are both AHT and CCB naïve will be included in the study as long as they meet the entry criteria of hypertension at baseline as described previously. To ensure that exposure to other AHT medicines is accounted for the cumulative dose-duration exposure of other prescribed AHT medicines (combined) will be captured in the same way as that described for CCB exposure. Thus in our study the following four exposure states will be possible during follow-up: (i) women with no exposure to either CCBs or other AHTs; (ii) women exposed to other AHTs but not exposed to CCBs; (iii) women exposed to CCBs but not exposed to other AHTs and (iv) women exposed (at any time) to both CCBs and AHTs. To compensate for immortal-time bias, and to be able to capture the dose-response nature of the association of CCB exposure with risk of breast cancer, a time-varying continuous ascertainment of cumulative exposure (dose-duration) to CCB (and separately other AHTs) will be captured as follows [34].

We will use the Anatomical Therapeutic Chemical (ATC) codes [35] and information on dispensed medicines in the PBS data for Australian longitudinal cohorts and medicine record data for the Rotterdam longitudinal cohorts to identify AHT drugs dispensed for the study population from study entry to exit. We will categorise AHT drugs dispensed into five main AHT drug classes: (i) beta-blocking agents (BB) (ATC code C07), (ii) CCB (C08); (iii) diuretics (C03), (iv) agents acting on the renin-angiotensin system

For peer review only - http://bmjopen.bmj.com/site/about/guidelines.xhtml

(ACEIs and ARBs) (C09), or (v) other AHTs not included in these classes regardless of treatment therapies (i.e., monotherapy or combination therapy). Details of level 5 ATC codes of the dispensing medicines are presented in Appendix Table 1.

For all longitudinal cohorts, the total cumulative duration exposed and the total cumulative dose of each AHT drug class will be calculated from the medicine records available from entry to exit of the study. For all cohorts the data includes information on the date of dispensing, quantity dispensed and the strength of the medicine (derived from the PBS item code for Australian data). In the Rotterdam data, the daily prescribed dose and prescribed duration of use are also available [28]. This information is not present in the Australian data necessitating estimation of the duration of the medicine. For the Australian data, the reverse waiting time distribution method with multiple random index dates will be used to determine the following: i) the median duration of medicine possession, defined as the number of days that 50% of prevalent users of the same medicine refill their scripts and ii) the upper duration limit of medicine possession, defined as the number of days that 80% of prevalent users of the same medicine refill their scripts [36, 37]. The median duration will be used as the usual possession duration of the medicine in calculating the total duration (days) of medicine possession in contiguous periods of exposure. The contiguous period will be defined as a series of dispensings of the same AHT class with no break between dispensing that is greater than the upper duration limit of medicine possession of the last medicine in the series. The total cumulative duration (days) of exposure to each AHT class under investigation for each individual over the study period will be the sum of the number of days accrued over all contiguous periods of exposure. The total cumulative dose will be similarly captured across all contiguous periods of exposure by summing the total milligrams (mg) of medicine dispensed of all dispensations (strength/unit multiplied by total units dispensed).

Contiguous periods of exposure will be accounted for any carry-over of over-supply. The carry-over will only be applied if the next dispensing is the same medicine (same ATC code at the fifth level) as suggested previously [38]. The start of contiguous periods will be deemed the date of first-time dispensing observed within a series. End of a contiguous period will be the last dispensing date in the series plus the usual duration of medicine possession and any eligible carry-over or the date of exit of

For peer review only - http://bmjopen.bmj.com/site/about/guidelines.xhtml

the study where the duration of medicine possession continues past this date. Details of cumulative dose and duration are presented in Figure 2.

In addition, time since last CCB exposure will be calculated in months from exit of the cohort back to the date of the last CCB exposure to differentiate between recent and previous exposure. This value will range from 0 for those with current exposure at the time of exit to the total of the follow-up time for those who are never exposed.

In the statistical model dose-duration will be captured by three elements, total cumulative duration, total cumulative dose and an interaction term to evaluate effect modification between duration and dose.

Outcome ascertainment

During follow-up, incident diagnoses of invasive breast cancer will be identified using the International Classification of Diseases (ICD) version 10 Australian Modification [39] for the Australian cohorts and the ICD-10 [40] for the Rotterdam Study. ICD-10 code C50.x (malignant neoplasm of breast) will be used to identify invasive breast cancer diagnosis across cancer registries and hospital admission data. In situ breast cancer (e.g. D05.x) will not be included in the outcome because hyperplasia and atypical lesions are not precancerous (although some are risk factors), and only some ductal carcinoma in situ are obligate precursors [41, 42].

Cancer registries for all cohorts contain, by statutory requirement, records of all cancers diagnosed or treated and are therefore considered internationally the 'gold standard' for cancer identification for research purposes. Since cancer diagnoses for Dutch women and cancer registry data for the Australian women are only currently available until 2016 and 2018 respectively, breast cancers diagnosed later than these dates will be identified from general practitioner data (Dutch women) and hospital data (Australian women). Hospital-derived diagnoses for breast cancer have a positive predictive value and sensitivity of 86% and specificity of 99.9% compared with the cancer registry in the 45 and Up Study population [43]. Sensitivity analyses showed that 53% of women with a cancer registry diagnosis had an inpatient diagnosis within the same calendar month and 93% within one calendar month, thus estimations of date of diagnosis align well with Cancer Registry dates.

The date of breast cancer diagnosis used in the study will be the date of diagnosis recorded in either the Australian Cancer Registry or the Rotterdam Study, or the date of the first hospital admission with a

For peer review only

principal diagnosis of breast cancer / date recorded in the GP data where registry/study data are unavailable.

Covariates

A wide range of socio-demographic, lifestyle and clinical potential confounders that may impact prescribing decisions for AHT medicines, a participant's decision to take prescribed AHT medicines or be associated with an increased risk of breast cancer will be included in the analysis [44]. Variables will include the following captured at baseline: age, marital status, highest qualification, socioeconomic disadvantage, remoteness, BMI, diabetes, heart disease, stroke, diagnosis of other cancer, comorbidity risk score, number of children and history of hysterectomy/both ovaries removed. In addition, the following time varying factors (updated annually throughout follow-up and at exit) will be included: statin use, use of combined hormonal contraception, use of combined hormonal replacement therapy. Confounding by comorbidity will be assessed at baseline and also as a time varying covariate as per the Rx-Risk score (with 5 years look back) that includes 46 clinical conditions based on ATC codes from prescribed medicine claims data, excluding AHT drugs as these medicines are captured in the exposure [45]. For the secondary aim (cohort-specific analyses) additional covariates will be available depending upon the cohort; for example for the 45 and Up Study cohort mammographic screening information will be used to differentiate screen-detected cancers, in the ALSWH and Rotterdam cohorts multiple waves of self-reported/clinical examination data will be used to update lifestyle information during follow-up. In addition, where possible we will explore the use of earlier study entry to provide longer follow up. For both Australian cohorts hospital admissions, all specialist services, and GPs visits will be used as time varying covariates to account for changing propensity to interact with health services. Differences between the cohort specific analysis and the harmonised analysis undertaken for each cohort will be evaluated to aid with determination of the influence of cohort specific variables on the association.

Details of the relevant codes (MBS item numbers (Australian women), ATC codes and ICD codes) used to identify participants' medical conditions, prescribed medicines and health service use from MBS, PBS/pharmacy data, hospital admission, GP data, cancer registries and mortality data collections are

For peer review only

provided in Appendix Table 2. A description of data items, questions and response categories from the baseline surveys of the ALSWH and the 45 and Up Study are presented in Appendix Table 3.

Statistical analysis

Primary aim: For the primary aim, analysis of the association between CCB use and the development of breast cancer, will be undertaken using Fine and Gray competing risks regression with incident breast cancer as the principal event, and death (from causes other than breast cancer) or bilateral mastectomy as the competing risk [46]. In this setting, the appropriate estimate of the probability of breast cancer is described by the cumulative incidence function rather than a hazard ratio. The models will account for confounding factors at entry using propensity scores to balance these factors across exposure status captured over the follow-up period. In addition, we will use standard covariate adjustment for time varying covariates. Any significant shifts in practice will be identified via the literature (and our clinical collaborators) and included as categorical variables in the models where appropriate.

Since the CCB exposure variable will be continuous rather than binary a generalisation of the binary propensity score, the generalised propensity score (GPS) developed by Hirano and Imbens, will be used [47]. Since CCB exposure will be sporadic, we will also evaluate the dose-duration response effect by further extending the multivariable models to incorporate a time-dependent dose-response component [48]. Specifically, the dose-duration response function will be estimated after adjusting for covariate imbalance by using the GPS methods applied in previous publications [48-51]. The non-linear predictive relationships between the dose-duration of CCB exposure and breast cancer will be modelled using nonlinear threshold models developed by Gannon & Harris [52]. Threshold models allow for the differential effect of the dose-duration of CCB exposure on the incidence of breast cancer with respect to an individual's duration and cumulative exposure to CCBs. The model will allow the coefficient (β) to vary according to the days/months/years per unit of the cumulative dose-duration while assuming that other components in the model are constant across all individuals to identify the thresholds for subpopulations [53]. The estimation of the threshold models involves searching all possible values of the dose-duration function with maximum likelihood estimators computed for every tried value. For practical purposes, the number of subpopulations will be limited to three or less [53]. The threshold model that minimises the Bayesian information criterion used for selection of the non-linear

For peer review only

models will be the final model. The counterfactual, individuals with no CCB exposure, will also be analysed as a baseline to show the impact of no versus any treatment using standard binary propensity score-matching of those exposed versus non-exposed [50].

Secondary aim: For the secondary aim we will explore relationships between dose-duration of CCB use and incident breast cancer and factors that confound and modify these relationships. Using multilevel modelling techniques and time-varying covariates, we will quantify the contributions of person-, temporal- and geo-spatial level-related factors to variation in the dose-duration effect. This will allow the development of both an inter- and intra-country-specific profiles of women who are at higher and lower risk of CCB-attributable breast cancer.

Analyses will be adjusted for multiple comparisons where appropriate. Where the sample size is insufficient to confirm the nature of the association, the results will be regarded as exploratory. Analyses will use Stata SE version 15 [54].

Subgroup analysis

In addition to our analysis of overall CCB exposure, the analysis for each aim will be conducted separately according to CCB class (i.e., dihydropyridines and non-dihydropyridines) and duration of action (i.e., short or long action) where sufficient statistical power exists.

Sensitivity analyses

We will conduct several sensitivity analyses. Since hyperplasia and atypical lesions are not precancerous, we will test the effect of adding ductal carcinoma in situ as a competing risk in sensitivity analyses rather than as an exclusion criterion. We will explore the impact of imposing various time lags, based on clinical consultation, between the start of follow-up on the outcome to account for latency and plausible timing between exposure and outcome development. The impact of decreased exposure due to imperfect adherence and prescribing at defined daily dose levels (a statistical measure of drug consumption, defined by the World Health Organization) will be explored in the dose-duration models and using sensitivity analyses [35]. We will expand the study population to women with prior CCB use at entry (captured as a starting dose-duration value using available look-back data) to examine the impact of CCB use (overall) on the outcome. Finally, because concessional status for subsidised medicines in Australia can change with age or employment status, we will explore varying the definition

For peer review only

For peer review only - http://bmjopen.bmj.com/site/about/guidelines.xhtml

of continuous PBS concessional status in Australian women to determine the impact this has on the association of CCB exposure with the outcome. The main analysis for the study will be evidence of 100% concessional status between 2003 and 2012 with a single year of no evidence allowed (i.e., at least one dispensation per year, and patient recorded as being on concessional status on each dispensation, with one year allowed to vary). This will be varied allowing two years of no evidence (the more relaxed scenario) and requiring 100% concessional status across all years (as the most stringent requirement). Sensitivity analyses will be applied to the primary aim in the first instance, and those factors found to be influential will be additionally tested in the secondary aim.

Sample size calculation

Breast cancer is a relatively common event for the studies under investigation, thus our sample size will be based on the women exposed to CCBs (as indicated by the numbers on the right-hand side of Figure 3). Using a conservative ratio of unexposed: exposed of 5:1, we have 95% confidence with 80% power to detect a minimum effect size of +/- 0.2 for breast cancer associated with CCB use in all datasets given the number of exposed individuals.

Individual patient data meta-analysis

For the primary aim a two-step (since the data cannot be brought together) random effects individual patient-level meta-analysis which allows for assessment of effect modification (i.e. interaction terms) by country or personal characteristics will be undertaken [55]. First, each individual study estimate will be calculated, plotted using a forest plot and compared to observe any similarities and differences. Next, individual estimates will be weighted using the Knapp-Hartung method and pooled using random effects methods taking into account both within and between variance as below [56]:

$\hat{\beta}_s = \beta + U_s + \varepsilon_s$, s = 1 to s studies

where β = mean true effect estimate, ε_s = within study variance, U_s = between study variance and $\hat{\beta}_s$ = estimated effect estimate in study s.

Patient and public involvement

There was no patient or public involvement in the design of the proposed study.

Ethics and dissemination

For peer review only

For peer review only - http://bmjopen.bmj.com/site/about/guidelines.xhtml

Ethical approval was obtained from the following Human research Ethics Committees: Curtin University (HRECs) (ref No. HRE2022-0335), NSW Population and Health Services Research Ethics Committee (2022/ETH01392 / 2022.31), ACT Research Ethics and Governance Office approval under National Mutual Acceptance for multi-jurisdictional data linkage research (2022.STE.00208). For the ALSWH survey collections has ongoing ethical approval from the HREC of the Universities of Newcastle and Queensland (approval numbers H076-0795 and 2004000224, respectively). The ALSWH also maintains institutional HREC approvals for external record linkage (approval numbers H-2011-0371 and 2012000132, respectively). In addition, access to national collections include MBS/PBS, National Death Index, Australian Cancer Database is approved by the Australian Institute of Health and Welfare. Access to state and territory data collections include hospital admissions and caner registries is approved by an appropriate HREC for each jurisdiction [63]. These data are approved by individual ethics committees and custodians in different jurisdictions, though the researchers make a single application to the ALSWH Data Access Committee, who then contact individual custodians on behalf of researchers. The Rotterdam Study data has been approved by the Medical Ethics Committee of the Erasmus MC (registration number MEC 02.1015) and by the Dutch Ministry of Health, Welfare and Sport (Population Screening Act WBO, license number 1071272-159521-PG). The Rotterdam Study Personal Registration Data collection is filed with the Erasmus MC Data Protection Officer under registration number EMC1712001.

Each of the studies collected informed consent from all participants at recruitment. Women who participated in earlier waves of the ALSWH provided consent for access to administrative health data including the MBS, PBS, national death index, cancer registries, and hospital records for most Australian States and Territories. In later waves, there has been a change to opt-out consent of these linkages, with participants provided information on the datasets that may be linked and a freecall phone number to opt out provided to participants at recruitment and in an annual newsletter. All study participants in the 45 and Up study consented to their survey data being linked to routinely collected health administrative data and health registry sources. All participants in the Rotterdam Study provided written informed consent to participate in the study and to have their information obtained from treating physicians.

For peer review only - http://bmjopen.bmj.com/site/about/guidelines.xhtml

Results of the proposed study will be published in high-impact journals and presented to key stakeholders via scientific meetings, lay press (print media, television, radio and internet).

Discussion

The equivocal literature regarding the potential association between CCB exposure and breast cancer is likely due to differences in study design (e.g., cohort vs case-control), length of follow-up, exposure measurement (e.g., ever vs never or time duration) or population characteristics (e.g. patients with hypertension and/or cardiovascular disease vs general population cohorts, which have differing risk of confounding by indication). These differences, along with small sample sizes in many studies (often n<1,000), make inference difficult. Randomised, controlled trials are not usually suitable for identifying long-term adverse effects of medicine use and long-term monitoring through observational studies using administrative datasets has become the standard way of overcoming this limitation for post-market surveillance research [57]. However, the sole use of administrative data limits the ability to control for critical lifestyle and other potential confounding factors that are not routinely reported in such datasets, e.g., BMI, physical activity, smoking and alcohol use history. In many studies, longitudinal cohorts have been used to alleviate this issue but often these studies have suffered from reporting and/or recall bias due to the reliance on self-report data, especially regarding the determination of exposure relating to prescription medication use. This has led to the preponderance of crude ever/never exposed to CCBs in the literature rather than more complex dose-duration measures that we propose.

Our study will involve six longitudinal cohorts of women extracted from internationally renowned Australian and Dutch studies linked with administrative health data, with complete coverage, to allow for continuous capture of CCB exposure for an extended period of time (i.e. up to 19 years). Using state-of-the-art analytical techniques such as dose-duration methods previously unused in this area, this study will evaluate the association and characterise the dose-response nature of any such association between long-term CCB use and the risk of breast cancer. A wide range of person-, temporal- and geo-spatial level-related factors will be measured and adjusted for to separate the effect of these factors from the relationship between CCB use and breast cancer. The analytic methods (non-linear threshold

For peer review only - http://bmjopen.bmj.com/site/about/guidelines.xhtml

483 modelling) proposed are novel and will be a significant innovation to pharmaco-epidemiological
484 analyses, which currently lack such sophisticated methods.

485 Due to the nature of the observational study design, confounding by indication will be unavoidable. To
486 minimise the impact of this issue, the study will be limited to participants with a diagnosis/self-reported
487 hypertension and comparisons will include participants exposed to other prescribed (non-CCB) AHT
488 medicines in addition to those who have no pharmacotherapy for hypertension. As explained in the
489 methods, the Australian cohorts will be restricted by concessional status to capture the AHT exposure
490 over the study duration, and our study is therefore likely prone to selection bias; this will be investigated
491 in the sensitivity analyses. Complete ascertainment of prior exposure will not be possible since we will
492 not have lifetime capture of prescribing data, therefore CCB exposure may be under-reported. Due to
493 the nature of medicine dispensing data, the level of certainty regarding whether the dispensed medicine
494 (e.g. combined hormonal contraceptives, combined menopausal hormone therapy and CCB / AHT
495 medicines) was consumed by the patient is unclear. The diagnostic interval of breast cancer may vary
496 between Australian and Dutch women, particularly since case ascertainment through cancer registries
497 can take months from the initial diagnosis to coding notifications. Previous studies have shown a
498 difference around 30 days in the diagnostic interval days depending on the presentation of symptoms
499 and the temporal differences in diagnostic pathways between the two countries [58].

500 While we have used the term "association" here, our use of GPS adjustment provides a mechanism for
501 undertaking analysis under a causal framework. Modelling each woman's propensity to receive the
502 exposure as a function of covariates means the GPS is independent of the outcomes and the study
503 represents a quasi-experiment. We prefer to use the terminology "association" when reporting our
504 results so as not to overstate our ability to adjust for potential confounding. Interpretation of any
505 association will incorporate the likelihood of causation (using the Bradford-Hill criteria) [59].

506 With regards to representativeness and generalisability of our results, leading epidemiologists [60] and
507 reputable peer-reviewed publications[61, 62] support the principle that cohort studies rely on the
508 validity of internal comparisons to avoid systematic error and need not be a random sample of a larger
509 population to provide generalisable knowledge. The validity of generalisation (i.e., external not internal

For peer review only - http://bmjopen.bmj.com/site/about/guidelines.xhtml

validity) depends on considerations about risk stratification and effect modification rather than bias/confounding. Thus, results of well-respected international cohort studies, such as the British Doctors' Study and US Nurses' Health Study, are regarded as providing generalisable knowledge despite their highly selected study samples.

Conclusion

The literature continues to debate whether or not CCBs increase the risk of breast cancer. The outcomes of the proposed study are likely to have considerable implications for both clinical practice and public health. If a positive association is found, this could have a global impact on clinical guidelines for treating hypertension in women. Long-term CCB use has the potential to become the number one major modifiable risk factor for breast cancer. Conversely if no association is found, this would provide reassurance to Australian and Dutch women that the current guidelines promoting CCBs as a first line therapy for hypertension are appropriate and that taking a CCB does not pose additional breast cancer risk.

For peer review only

For peer review only - http://bmjopen.bmj.com/site/about/guidelines.xhtml

525 **References**

526 1. Sung H, Ferlay J, Siegel RL, Laversanne M, Soerjomataram I, Jemal A, et al. Global cancer statistics 2020: GLOBOCAN estimates of incidence and mortality worldwide for 36 cancers in 185 countries. CA: a cancer journal for clinicians. 2021;71(3):209-49.

529 2. Arnold M, Morgan E, Rumgay H, Mafra A, Singh D, Laversanne M, et al. Current and future burden of breast cancer: Global statistics for 2020 and 2040. The Breast. 2022;66:15-23.

531 3. Fuchs FD, Whelton PK. High blood pressure and cardiovascular disease. Hypertension. 2020;75(2):285-92.

533 4. Lopez A, Mathers C, Ezzati M, Jamison D, Murray C. Global and regional burden of disease and risk factors, 2001: systematic analysis of population health data. Lancet. 2006;367:1747-57.

535 5. World Health Organisation. A global brief on hypertension: Silent killer, global public health crisis. Document number : WHO/DCO/WHD/2013.2 Geneva: WHO; 2013.

537 6. Visseren FL, Mach F, Smulders YM, Carballo D, Koskinas KC, Bäck M, et al. 2021 ESC Guidelines on cardiovascular disease prevention in clinical practice: Developed by the Task Force for cardiovascular disease prevention in clinical practice with representatives of the European Society of Cardiology and 12 medical societies With the special contribution of the European Association of Preventive Cardiology (EAPC). European heart journal. 2021;42(34):3227-337.

542 7. National Vascular Disease Prevention Alliance. Guidelines for the management of absolute cardiovascular disease risk. 2012.

544 8. Daling JR. Calcium channel blockers and cancer: is an association biologically plausible? American journal of hypertension. 1996;9(7):713-4.

546 9. Wyllie AH. Glucocorticoid-induced thymocyte apoptosis is associated with endogenous endonuclease activation. Nature. 1980;284(5756):555-6.

548 10. Szalai G, Krishnamurthy R, Hajnóczky G. Apoptosis driven by IP3-linked mitochondrial calcium signals. The EMBO journal. 1999;18(22):6349-61.

550 11. Suzuki J, Umeda M, Sims PJ, Nagata S. Calcium-dependent phospholipid scrambling by TMEM16F. Nature. 2010;468(7325):834-8.

552 12. Parton M, Dowsett M, Smith I. Studies of apoptosis in breast cancer. Bmj. 2001;322(7301):1528-32.

554 13. Guo D-Q, Zhang H, Tan S-J, Gu Y-C. Nifedipine promotes the proliferation and migration of breast cancer cells. PloS one. 2014;9(12):e113649.

556 14. Altamirano F, Valladares D, Henríquez-Olguín C, Casas M, López JR, Allen PD, et al. Nifedipine treatment reduces resting calcium concentration, oxidative and apoptotic gene expression, and improves muscle function in dystrophic mdx mice. PloS one. 2013;8(12):e81222.

For peer review only - http://bmjopen.bmj.com/site/about/guidelines.xhtml

15. Wright CM, Moorin RE, Chowdhury EK, Stricker BH, Reid CM, Saunders CM, et al. Calcium channel blockers and breast cancer incidence: An updated systematic review and meta-analysis of the evidence. Cancer epidemiology. 2017;50:113-24.

16. Li CI, Daling JR, Tang M-TC, Haugen KL, Porter PL, Malone KE. Use of antihypertensive medications and breast cancer risk among women aged 55 to 74 years. JAMA internal medicine. 2013;173(17):1629-37.

17. Grimaldi-Bensouda L, Klungel O, Kurz X, De Groot MC, Afonso ASM, De Bruin ML, et al. Calcium channel blockers and cancer: a risk analysis using the UK Clinical Practice Research Datalink (CPRD). BMJ open. 2016;6(1):e009147.

18. Wilson LE, D'Aloisio AA, Sandler DP, Taylor JA. Long-term use of calcium channel blocking drugs and breast cancer risk in a prospective cohort of US and Puerto Rican women. Breast Cancer Research. 2016;18(1):1-8.

19. Raebel MA, Zeng C, Cheetham TC, Smith DH, Feigelson HS, Carroll NM, et al. Risk of breast cancer with long-term use of calcium channel blockers or angiotensin-converting enzyme inhibitors among older women. American Journal of Epidemiology. 2017;185(4):264-73.

20. Gómez-Acebo I, Dierssen-Sotos T, Palazuelos C, Perez-Gomez B, Lope V, Tusquets I, et al. The use of antihypertensive medication and the risk of breast cancer in a case-control study in a Spanish population: the MCC-Spain study. PLoS One. 2016;11(8):e0159672.

21. Xie Y, Wang M, Xu P, Deng Y, Zheng Y, Yang S, et al. Association between antihypertensive medication use and breast cancer: a systematic review and meta-analysis. Frontiers in pharmacology. 2021;12:609901.

22. National Heart Foundation of Australia. Guideline for the diagnosis and management of hypertension in adults – 2016. Melbourne: National Heart Foundation of Australia. 2016.

23. Smulders Y, Burgers J, Scheltens T, Van Hout B, Wiersma T, Simoons M. Clinical practice guideline for cardiovascular risk management in the Netherlands. 2008.

24. Wagemaakers FN, Hollingworth SA, Kreijkamp-Kaspers S, Tee EH, Leendertse AJ, van Driel ML. Opioid analgesic use in Australia and the Netherlands: a cross-country comparison. International Journal of Clinical Pharmacy. 2017;39:874-80.

25. Platt RW, Platt R, Brown JS, Henry DA, H. KO, Suissa S. How pharmacoepidemiology networks can manage distributed analyses to improve replicability and transparency and minimize bias. Pharmacoepidemiol Drug Safety. 2018;29(S1):3-7.

26. Dobson AJ, Hockey R, Brown WJ, Byles JE, Loxton DJ, McLaughlin D, et al. Cohort profile update: Australian longitudinal study on women's health. Int J Epidemiol. 2015;44(5):1547-f.

27. Bleicher K, Summerhayes R, Baynes S, Swarbrick M, Navin Cristina T, Luc H, et al. Cohort Profile Update: The 45 and Up Study. Int J Epidemiol. 2022.

For peer review only

For peer review only - http://bmjopen.bmj.com/site/about/guidelines.xhtml

28. Ikram MA, Brusselle G, Ghanbari M, Goedegebure A, Ikram MK, Kavousi M, et al. Objectives, design and main findings until 2020 from the Rotterdam Study. European journal of epidemiology. 2020;35:483-517.

29. Paige E, Kemp-Casey A, Korda R, Banks E. Using Australian Pharmaceutical Benefits Scheme data for pharmacoepidemiological research: challenges and approaches. 2015.

30. Rolland B, Reid S, Stelling D, Warnik G, Thornquist M, Feng Z, et al. Toward Rigorous Data Harmonization in Cancer Epidemiology Research: One Approach. American Journal of Epidemiology. 2015;182(12):1033-8.

31. Fortier I, Raina P, Van den Heuvel ER, Griffith LE, Craig C, Saliba M, et al. Maelstrom Research guidelines for rigorous retrospective data harmonization. Int J Epidemiol. 2017;46(1):103-5.

32. Assimes TL, Elstein E, Langleben A, Suissa S. Long-term use of antihypertensive drugs and risk of cancer. Pharmacoepidemiology and drug safety. 2008;17(11):1039-49.

33. Sendor R, Stürmer T. Core concepts in pharmacoepidemiology: confounding by indication and the role of active comparators. Pharmacoepidemiology and drug safety. 2022;31(3):261-9.

34. Zhou Z, Rahme E, Abrahamowicz M, Pilote L. Survival bias associated with time-to-treatment initiation in drug effectiveness evaluation: a comparison of methods. American journal of epidemiology. 2005;162(10):1016-23.

35. WHO Collaborating Centre for Drug Statistics Methodology. ATC classification index with DDDs Oslo, Norway 2023 [Available from: https://www.whocc.no/atc_ddd_index_and_guidelines/atc_ddd_index/.

36. Pottegård A, Hallas J. Assigning exposure duration to single prescriptions by use of the waiting time distribution. Pharmacoepidemiology and drug safety. 2013;22(8):803-9.

37. Bødkergaard K, Selmer RM, Hallas J, Kjerpeseth LJ, Skovlund E, Støvring H. Using multiple random index dates with the reverse waiting time distribution improves precision of estimated prescription durations. Pharmacoepidemiology and Drug Safety. 2021;30(12):1727-34.

38. Arnet I, Greenland M, Knuiman MW, Rankin JM, Hung J, Nedkoff L, et al. Operationalization and validation of a novel method to calculate adherence to polypharmacy with refill data from the Australian pharmaceutical benefits scheme (PBS) database. Clinical epidemiology. 2018:1181-94.

39. Chronicle of The International Statistical Classification of Diseases and Related Health Problems, Tenth Revision, Australian Modification (ICD-10-AM) First Edition to Eleventh Edition [Internet]. 2019 [cited 22/08/2023]. Available from: https://www.ihacpa.gov.au/sites/default/files/2022-08/icd-10-am_chronicle_-_eleventh_edition.pdf.

40. Organization WH. International Statistical Classification of Diseases and related health problems: Alphabetical index: World Health Organization; 2004.

41. To T, Wall C, Baines CJ, Miller AB. Is carcinoma in situ a precursor lesion of invasive breast cancer? International journal of cancer. 2014;135(7):1646-52.

For peer review only

42. Dettogni RS, Stur E, Laus AC, da Costa Vieira RA, Marques MMC, Santana IVV, et al. Potential biomarkers of ductal carcinoma in situ progression. BMC cancer. 2020;20(1):1-9.
43. Kemp A, Preen DB, Saunders C, Holman CDAJ, Bulsara M, Rogers K, et al. Ascertaining invasive breast cancer cases; the validity of administrative and self-reported data sources in Australia. BMC medical research methodology. 2013;13(1):1-8.
44. Cancer Australia. Risk factors for breast cancer: A review of the evidence, Cancer Australia, Surry Hills, NSW. 2018.
45. Pratt NL, Kerr M, Barratt JD, Kemp-Casey A, Ellett LMK, Ramsay E, et al. The validity of the Rx-Risk comorbidity index using medicines mapped to the anatomical therapeutic chemical (ATC) classification system. BMJ open. 2018;8(4):e021122.
46. Fine J, Gray R. A proportional hazards model for the subdistribution of a competing risk. Journal of the American Statistical Association. 1999;94:496-509.
47. Hirano K, Imbens GW. The propensity score with continuous treatments. Applied Bayesian modeling and causal inference from incomplete-data perspectives. 2004;226164:73-84.
48. Hirano K, Imbens G. The propensity score with continuous treatments. In: Gelman A, Meng X, editors. Applied Bayesian Modeling and Causal Inference from Incomplete-Data Perspectives. Sussex: Wiley Interscience 2004. p. 73-84.
49. Guardabascio B, Ventura M. Estimating the dose-response function through a generalized linear model approach. STATA Journal. 2014;14(1):141-58.
50. Kluve J, Schneider H, Uhlendorff A, Zhao ZW. Evaluating continuous training programmes by using the generalized propensity score. J Royal Stat Soc. 2007;172(2):587-617.
51. Flores C, Flores-Lagunes A, Gonzalez A, Neumann T. Estimating the Effects of Length of Exposure to Instruction in a Training Program: The Case of Job Corps. Review of Economics and Statistics. 2011;94(1):153-71.
52. Gannon B, Harris D, Harris M. Threshold effects in nonlinear models with an application to the social capital-retirement-health relationship. Health Economics. 2014;23(9):1072-83.
53. Bessaoud F, Daures J, Molinari N. Free knot splines for logistic models and threshold selection. Computer Methods and Programs in Biomedicine. 2005;77(1):1-9.
54. StataCorp. Stata Statistical Software: Release 15. College Station, TX: StataCorp LLC; 2017.
55. Thomas D, Radji S, Benedetti A. Systematic review of methods for individual patient data meta-analysis with binary outcomes. BMC Medical Research methodology. 2014;14(79):1471-2288.
56. Bender R, Friede T, Koch A, Kuss O, Schlattmann P, Schwarzer G, et al. Methods for evidence synthesis in the case of very few studies. Research Synthesis Methods. 2018;9(3):382-92.
57. Avorn J. In defense of pharmacoepidemiology—embracing the yin and yang of drug research. New England Journal of Medicine. 2007;357(22):2219-21.
58. Vedsted P, Weller D, Falborg AZ, Jensen H, Kalsi J, Brewster D, et al. Diagnostic pathways for breast cancer in 10 International Cancer Benchmarking Partnership (ICBP) jurisdictions: an

For peer review only

international comparative cohort study based on questionnaire and registry data. BMJ open. 2022;12(12):e059669.

59. Hill AB. The environment and disease: association or causation? : Sage Publications; 1965.

60. Rothman KJ, Greenland S, Lash TL. Modern epidemiology: Wolters Kluwer Health/Lippincott Williams & Wilkins Philadelphia; 2008.

61. Willett WC, Blot WJ, Colditz GA, Folsom AR, Henderson BE, Stampfer MJ. Merging and emerging cohorts: not worth the wait. Nature. 2007;445(7125):257-8.

62. Mealing NM, Banks E, Jorm LR, Steel DG, Clements MS, Rogers KD. Investigation of relative risk estimates from studies of the same population with contrasting response rates and designs. BMC medical research methodology. 2010;10:1-12.

63. Australian Longitudinal Study on Women's Health. Current HREC approvals for ALSWH data linkage [4/07/2023]. Available from: https://alswh.org.au/alswh-hrec-approvals/.

For peer review only

Authors' contributions: CH assisted in the study design and drafted the manuscript. NH and DY contributed to drafting sections relating to study design and methodology. RM, SP, DP, BHS, CS, WA, CR, JH, MKB, DY conceptualised, designed the study, obtained the funding, reviewed, and revised the manuscript. All authors revised the manuscript for important intellectual content. The author(s) read and approved the final manuscript.

Funding statement: This work was supported by The National Health and Medical Research Council, project grant APP2014896. The study funders had no role in the study design, conduct, manuscript writing or decision to submit for publication.

Competing interests: No conflict of interest

Acknowledgements:

The research on which this manuscript is based was conducted as part of the Australian Longitudinal Study on Women's Health by the University of Queensland and the University of Newcastle. We are grateful to the Australian Government Department of Health and Aged Care for funding and to the women who provided the survey data.

This research was also completed using data collected through the 45 and Up Study (www.saxinstitute.org.au). The 45 and Up Study is managed by the Sax Institute in collaboration with major partner Cancer Council NSW, and partners the Heart Foundation and the NSW Ministry of Health. We thank the many thousands of people participating in the 45 and Up Study. The 45 and Up Study baseline data were linked by the Centre for Health Record Linkage (CHeReL) www.cherel.org.au.

The authors acknowledge the Rotterdam Study. The Rotterdam Study is funded by Erasmus Medical Center and Erasmus University, Rotterdam, Netherlands Organization for the Health Research and Development (ZonMw), the Research Institute for Diseases in the Elderly (RIDE), the Ministry of Education, Culture and Science, the Ministry for Health, Welfare and Sports, the European Commission (DG XII), and the Municipality of Rotterdam. The authors are grateful to the study participants, the staff from the Rotterdam Study and the participating general practitioners and pharmacists.

The authors are grateful Maz Osborne and John Stubbs for their consumer opinions on developing the manuscript.

For peer review only

710 **Availability of data and materials:** This research will be completed using data collected through the Australian Longitudinal study on Women's Health (https://alswh.org.au/), the 45 and Up Study (www.saxinstitute.org.au), and the Rotterdam Study (https://www.erasmusmc.nl/en/research/core-facilities/ergo-the-rotterdam-study). The data that support the analyses of this study depend on the availability of these data, which will be used under license for the current study, and so are not publicly available. Information on the process for accessing these data can be found in the websites mentioned above.

For peer review only

Table 1. Sources of data for the study

Data source	Socio-demographic characteristics	Lifestyle characteristics	Clinical characteristics	Reproductive Health	Family history of cancer and genetic predisposition	Health care utilisation	Diagnosis of Breast cancer	Medication use	Death
Self-report survey / clinical examinations	x	x	x	x	x		x	x	
PBS / Pharmacy data			x					x	
Cancer Registry data			x				x		
MBS data			x			x			
GP Records			x			x	x		
Hospital admission data			x			x	x		
Mortality register data									x

PBS: Pharmaceutical Benefits Scheme, MBS: Medicare Benefits Schedule, GP: general practitioner.

For peer review only

For peer review only - http://bmjopen.bmj.com/site/about/guidelines.xhtml

720 **Table 2. Variables for harmonised and cohort-specific analyses**

	ALSWH		45 and Up Study	Rotterdam Study			Harmonisable status
	1946-51 Cohort	1921-26 Cohort		RS-I-4	RS-II-2	RS-III-1	
**Socio-demographic characteristics**							
Age	Y	Y	Y	Y	Y	Y	Y*
Marital status	Y	Y	Y	Y	Y	Y	Y*
Country of birth	Y	Y	Y	N	N	N	N
Socioeconomic disadvantage	Y	Y	Y	Y	Y	Y	Y*
Remoteness	Y	Y	Y	NA	NA	NA	Y*
Work status	N	N	Y	Y	Y	Y	N
Education status	Y	Y	Y	Y	Y	Y	Y*
**Lifestyle characteristics**							
Body mass index (height/weight)	Y	Y	Y	Y	Y	Y	Y*
Physical activity	Y	Y	Y	N	N	Y	N
Smoking status	Y	N	Y	Y	Y	Y	N
Alcohol drinks per week	Y	N	Y	Y	Y	Y	N
Shift work at night	Y	N	N	N	N	N	N
**Clinical characteristics**							
High BP	Y	Y	Y	Y	Y	Y	Y*
Systolic BP/Diastolic BP	N	N	N	Y	Y	Y	N
Blood cholesterol level	N	N	N	Y	Y	Y	N
Diabetes	Y	Y	Y	Y	Y	Y	Y*
Heart disease	Y	Y	Y	Y	Y	Y	Y*
Stroke	Y	Y	Y	Y	Y	Y	Y*
Family history of heart disease	N	N	Y	Y	Y	Y	N
Family history of stroke	N	N	Y	Y	Y	Y	N
Mastectomy	Y	Y	Y	Y	Y	Y	Y*
**Reproductive health**							
Age at menarche	Y	N	N	Y	Y	Y	N
Age at menopause	Y	N	Y	Y	Y	Y	N
Age when had first child	Y	N	Y	Y	Y	Y	N
Number of children	Y	Y	Y	Y	Y	Y	Y*
History of hysterectomy/ both ovaries removed	Y	Y	Y	Y	Y	Y	Y*
Breastfeeding	N	N	Y	Y	Y	N	N
**Healthcare utilisation**							
Concession status	Y	Y	Y	NA	NA	NA	Y*
Mammogram	Y	N	Y	N	N	N	N
Hospital admissions	Y	Y	Y	N	N	N	N
Specialist/consulting physician attendances	Y	Y	Y	N	N	N	N
GPs attendances	Y	Y	Y	N	N	N	N
**Diagnosis of cancer**							

For peer review only - http://bmjopen.bmj.com/site/about/guidelines.xhtml

	ALSWH		45 and Up	Rotterdam Study			
A diagnosis/history of invasive breast cancer	Y	Y	Y	Y	Y	Y	Y*
A diagnosis/history of non-invasive breast cancer (LCIS, DCIS)	Y	Y	Y	Y	Y	Y	Y*
Other cancer	Y	Y	Y	Y	Y	Y	Y*
**Medicine use**							
CCB	Y	Y	Y	Y	Y	Y	Y*
Non-CCB (ACEI, ARB, BB, diuretics, others)	Y	Y	Y	Y	Y	Y	Y*
Statins	Y	Y	Y	Y	Y	Y	Y*
Current use of combined hormonal contraception	Y	Y	Y	Y	Y	Y	Y*
Current use of combined menopausal hormone therapy	Y	Y	Y	Y	Y	Y	Y*
Comorbidity (Rx-Risk) score	Y	Y	Y	Y	Y	Y	Y*
**Mortality**							
Death	Y	Y	Y	Y	Y	Y	Y*

ALSWH: Australian Longitudinal Study on Women's Health, Y: Yes, data available, N: No data available, NA: not applicable, BP: blood pressure, CVD: Cardiovascular disease, GPs: General practitioners, FNA: Fine-needle aspiration, LCIS: Lobular carcinoma in situ, DCIS: Ductal carcinoma in situ, CCB: Calcium channel blockers, ACEI: Angiotensin-converting-enzyme inhibitors, ARB: Angiotensin receptor blockers, BB: Beta blockers.

*: These variables will be used in the harmonised analysis. ** Comorbidity (Rx-Risk) score includes 46 clinical conditions based on prescribed medicine claims data.

Note: This table includes all potential confounders available from ALSWH (https://alswh.org.au/for-data-users/data-documentation/data-dictionary/), 45 and Up Study (https://www.saxinstitute.org.au/solutions/45-and-up-study/use-the-45-and-up-study/data-and-technical-information/), and Rotterdam Study (https://www.erasmusmc.nl/en/research/core-facilities/ergo-the-rotterdam-study).

For peer review only

List of figures

Figure 1. Schematic diagram showing entry, exposure, and exit of the study populations across longitudinal cohorts

Figure 2. Capture cumulative dose and duration exposed for each antihypertensive medicine

Figure 3. Sample size calculation

For peer review only

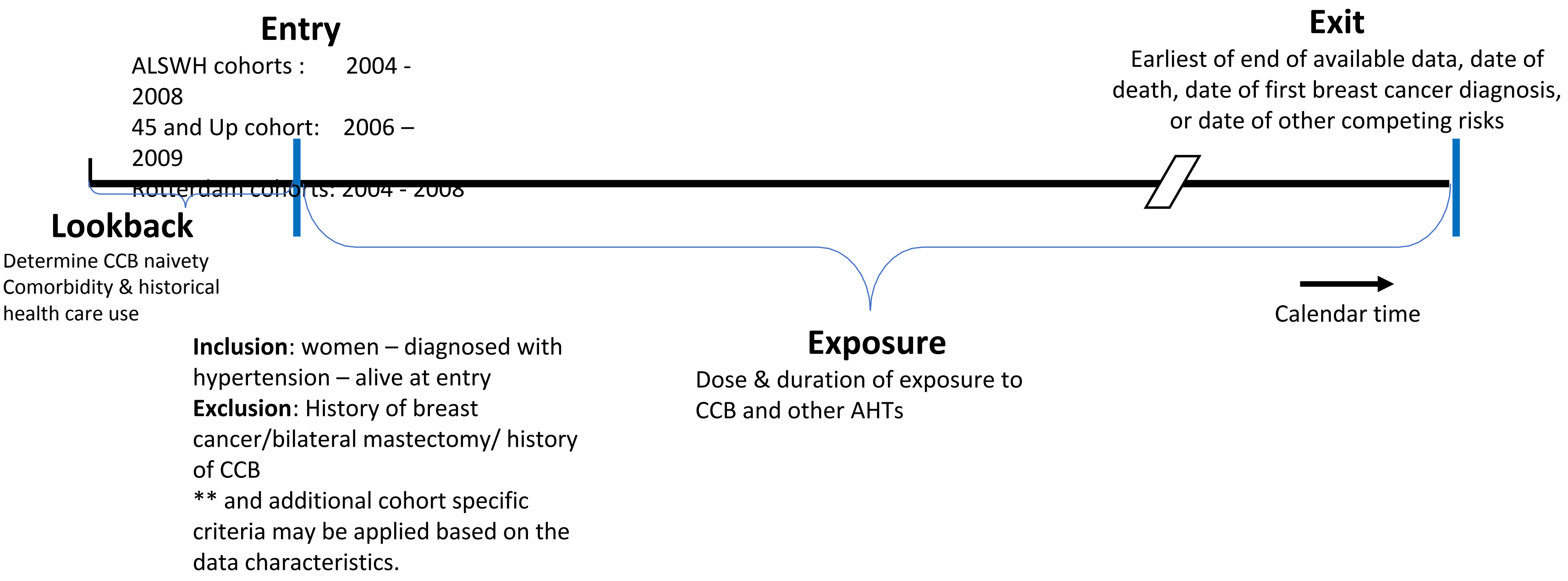

Figure 1. Schematic diagram showing entry, exposure, and exit of the study populations across longitudinal cohorts

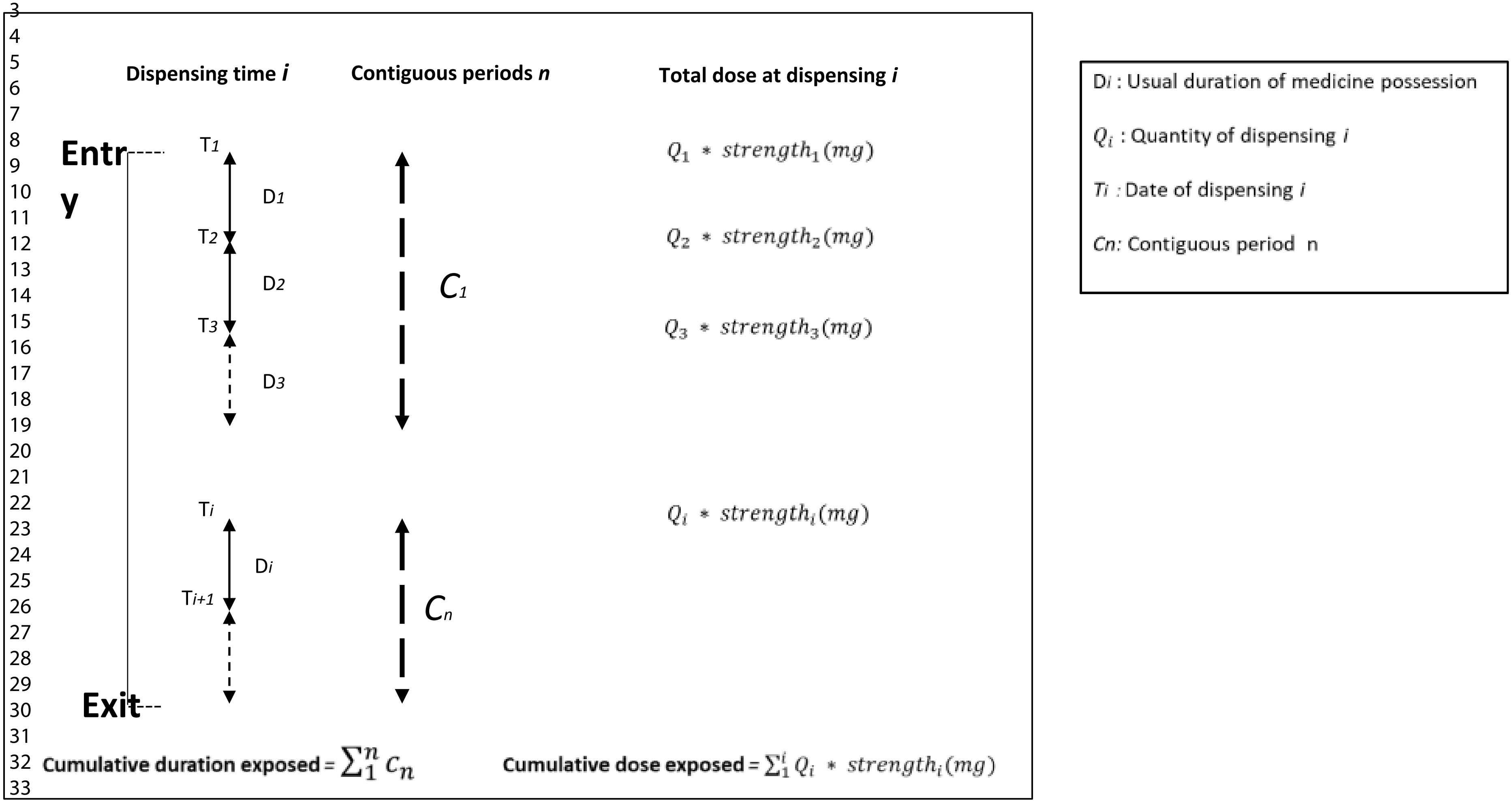

Figure 2. Capture cumulative dose and duration exposed for each antihypertensive medicine

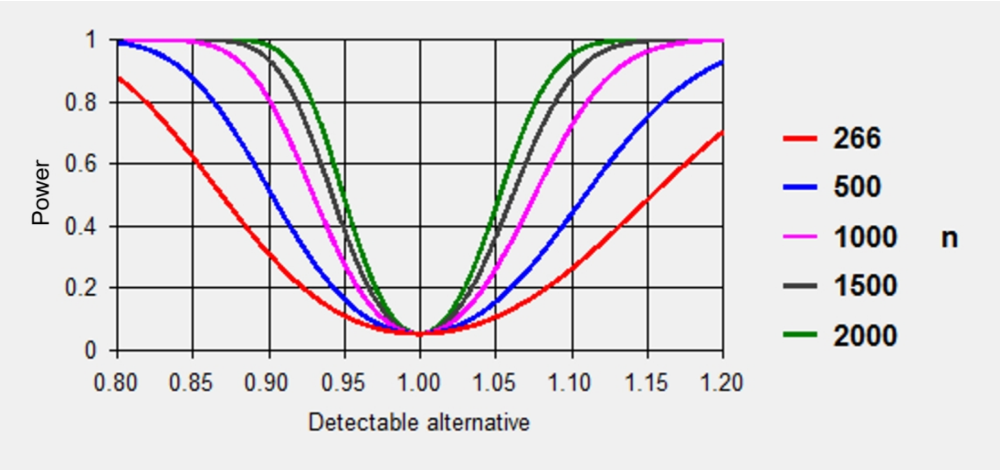

Sample size calculation

692x323mm (59 x 59 DPI)

Title: Association between long-term use of calcium channel blockers (CCB) and the risk of breast cancer: A retrospective longitudinal observational study protocol

Appendix Table 1. Classification of prescribed oral antihypertensive medicines used based on the Anatomical Therapeutic Chemical codes

Class	ATC code	Drug name	Usual dose range mg/day	DDD (mg)	Usual daily frequency*
ACEIs	C09AA01	Captopril	25-100	50	2
	C09AA02	Enalapril	5-40	10	1-2
	C09AA09	Fosinopril	10-40	15	1
	C09AA03	Lisinopril	10-40	10	1
	C09AA04	Perindopril	4-8	4	1
	C09AA06	Quinapril	10-80	15	1-2
	C09AA05	Ramipril	2.5-20	2.5	1-2
	C09AA10	Trandolapril	1-4	2	1
	C09AA07	Benazepril	10-40	7.5	1
	C09AA13	Moexipril	7.5-30	15	1
ARBs	C09CA06	Candesartan	8-32	8	1
	C09CA02	Eprosartan	400-800	600	1-2
	C09CA04	Irbesartan	150-300	150	1
	C09CA01	Losartan	25-100	50	1-2
	C09CA08	Olmesartan Medoxomil	20-40	20	1
	C09CA07	Telmisartan	20-80	40	1
	C09CA03	Valsartan	80-320	80	1-2
BBs	C07AB03	Atenolol	25-100	75	1-2
	C07AB05	Betaxolol	5-20	20	1
	C07AB07	Bisoprolol	2.5-10	10	1
	C07AG02	Carvedilol	12.5-50	37.5	2
	C07AG01	Labetalol	200-800	600	2
	C07AB02	Metoprolol<br>Metoprolol ER	50-100<br>50-100	150<br>150	1-2<br>1
	C07AA12	Nadolol	40-120	160	1
	C07AB12	Nebivolol	5	5	1
	C07AA02	Oxprenolol	40-160	160	2
	C07AA03	Pindolol	10-40	15	2-3
	C07AA05	Propranolol<br>Propranolol LA	40-160<br>160-180	160<br>160	2<br>1
	C07AA06	Timolol	20-40	20	2
	C07AB04	Acebutolol	200-800	400	2
	C07AA23	Penbutolol	10-40	40	1
CCBs-DHP	C08CA01	Amlodipine	2.5-10	5	1
	C08CA02	Felodipine	2.5-20	5	1
	C08CA13	Lercanidipine	10-20	10	1
	C08CA05	Nifedipine<br>Nifedipine LA	10-40<br>20-120	 30	2<br>1
	C08CA03	Isradipine	2.5-10	5	2
	C08CA04	Nicardipine ER	60-120	90	2

For peer review only - http://bmjopen.bmj.com/site/about/guidelines.xhtml

Class	ATC code	Drug name	Usual dose range mg/day	DDD (mg)	Usual daily frequency*
	C08CA07	Nisoldipine	10-40	20	1
	C10BX03	Atorvastatin And Amlodipine *	-	-	1
CCB-nonDHP	C08DA01	Verapamil<br>Verapamil LA<br>Verapamil IR	120-360<br>120-480<br>80-320	240<br>240<br>240	1<br>1-2<br>2
	C08DB01	Diltiazem ER<br>Diltiazem LA<br>Diltiazem	180-420<br>120-540<br>180-240	240	1<br>1<br>3-4
Diuretics	C03DB01	Amiloride	5-10	10	1-2
	C03EA01	Amiloride + HCTZ<br>Triamterene + HCTZ	-	-	1-2
	C03DB02	Triamterene	50-100	100	1-2
	C03AA04	Chlorothiazide	125-500	500	1-2
	C03AA05	Polythiazide	2-4	1	1
	C03AA01	Bendroflumethiazide	2.5-10	2.5	1
	C03AA03	HCTZ	12.5-50	25	1
	C03BA04	Chlortalidone	12.5-25	25	1
	C03BA11	Indapamide	1.25-2.5	2.5	1
	C03BA08	Metolazone**	0.5-1.0<br>2.5-5	5	1
	C03CA01	Furosemide **	20-80	40	2
	C03CA02	Bumetanide **	0.5-2	1	2
	C03CA04	Torsemide **	2.5-10	15	1
	C03DA01	Spironolactone **	25-50	75	1
	C03DA04	Eplerenone **	50-100	50	1-2
	C03CC01	Etacrynic acid	50-200	50	1-2
	C03XA01	Tolvaptan	15-60	30	1
Other ATH	C02AC01	Clonidine	0.1-0.8	0.5	2
	C02AC02	Guanfacine	0.5-2	3	1
	C02DB02	Hydralazine	25-100	100	2
	C02AB01	Methyldopa	250-1000	1000	2-4
	C02AA02	Reserpine	0.1-0.25	0.5	1
	C02DC01	Minoxidil	2.5-80	20	1-2
	C02AC05	Moxonidine	0.2-0.6	0.3	1-2
	C02CA01	Prazosin	2-20	5	2-3
	C02CA04	Doxazosin	1-16	4	1
	G04CA03	Terazosin	1-20	5	1-2
Combination	C09BB02	Lercanidipine + Enalapril	-	-	1
	C09BB04	Perindopril + Amlodipine	-	-	1
	C09BB05	Ramipril + Felodipine	-	-	1
	C09BB10	Trandolapril + Verapamil	-	-	1
	C09BA09	Fosinopril + HCTZ	-	-	1

For peer review only

Class	ATC code	Drug name	Usual dose range mg/day	DDD (mg)	Usual daily frequency*
	C09BA04	Perindopril + Indapamide	-	-	1
	C09BA02	Enalapril + HCTZ	-	-	1
	C09BA06	Quinapril + HCTZ	-	-	1
	C09DX04	Valsartan + Sacubitril	-	-	2
	C09DB01	Amlodipine + Valsartan	-	-	1
	C09DB02	Olmesartan + Amlodipine	-	-	1
	C09DB04	Telmisartan + Amlodipine	-	-	1
	C09DX01	Amlodipine + Valsartan + HCTZ	-	-	1
	C09DX03	Olmesartan + Amlodipine + HCTZ	-	-	1
	C09DA06	Candesartan + HCTZ	-	-	1
	C09DA02	Eprosartan + HCTZ	-	-	1
	C09DA04	Irbesartan + HCTZ	-	-	1
	C09DA08	Olmesartan + HCTZ	-	-	1
	C09DA07	Telmisartan + HCTZ	-	-	1
	C09DA03	Valsartan +HCTZ	-	-	1

HCTZ: hydrochlorothiazide. ATC: Anatomical Therapeutic Chemical, RAS: Renin Angiotensin System, CCB-DHP: calcium channel blockers-dihydropyridines, CCB-nonDHP: calcium channel blockers-nondihydropyridines, BB: beta blockers, ER: extended release, LA: long acting, IR: immediate release, DDD: Define Daily Dose, *: usual daily frequency is based on recommendations by the seventh report of the joint national committee on prevention, detection, evaluation, and treatment of high blood pressure (2003), **: These drugs are used for other indications (but have an antihypertensive effect).

For peer review only

Title: Association between long-term use of calcium channel blockers (CCB) and the risk of breast cancer: A retrospective longitudinal observational study protocol

Appendix Table 2. Codes used to identify participants' diagnosis and procedures in linked data sources

Conditions	MBS item number (for diagnoses or procedures recorded in Australian MBS data)	Procedure codes - ACHI 10 (for procedures recorded in Australian hospital admission data)	ATC codes (for medicines recorded in pharmacy data and study examination - Rotterdam)	ICD code (for diagnoses recorded in study examination-Rotterdam, hospital, cancer, and mortality data)	
				ICD-9-CM/ ICD-9	ICD-10-AM disease code/ ICD-10
**Diabetes**	MBS claims for:<br>- Diabetes annual cycle of care: 2517, 2518, 2521, 2522, 2525, 2526, 2620, 2622, 2624, 2631, 2633, 2635, 259 – 264.<br>- HbA1C test: 66551, 73840<br>- Comprehensive reassessment of eyes in a patient with diabetes mellitus: 10915, 12325, 12326.<br>- Diabetes education: 81100, 81105<br>- Exercise physiology health service for the management of type 2 diabetes: 81110, 81115<br>- Dietetics health service for the management of type 2 diabetes: 81120, 81125	-	+ Anti-diabetic Therapies: A10	+ Secondary diabetes melllitus:249<br>+ Diabetes mellitus: 250	+ Type 1 diabetes mellitus: E10<br>+ Type 2 diabetes mellitus: E11<br>+ Other specified diabetes Mellitus: E13<br>+Unspecified diabetes mellitus: E14
**Heart disease**	MBS claims for:<br>-Percutaneous transluminal coronary angioplasty, item 35304, 35305, 35310, 35335, 35338, 35341, 35344, 38300, 38303, 38306 38309, 38312, 38315, 38318.<br>- Coronary artery bypass graft, item 38497 – 38504.<br>- Selective coronary angiography, item 38215 – 38246	-	+ Digoxin: C01AA05<br>+ Isosorbide mononitrate: C01DA14<br>+ Glyceryl trinitrate: C01DA02	+ Ischemic heart disease: 410-414<br>+ Heart failure: 428	+ Ischemic heart disease: I20-I25<br>+ Heart failure: I50

**Stroke**	-	-	-	+ Cerebrovascular diseases: 430-438	+ Cerebrovascular diseases: I60-I69
**Hysterectomy***	MBS claims for hysterectomy:<br>Laparoscopic abdominal hysterectomy: 35751, 35753, 35754, 35756<br>Abdominal hysterectomy: 35653, 35661, 35668, 35669, 35671, 35729<br>Vaginal hysterectomy replay: 35657, 35673, 35750	Laparoscopic abdominal hysterectomy: 90448-00, 90448–01, 90448–02<br>Abdominal hysterectomy: 35653-00, 35653–01, 35653–04, 35661–00, 35670–00, 35667–00, 35664–00.<br>Vaginal hysterectomy: 35750-00, 35753-02, 35657–00, 35673–02, 35667–01, 35664–01.<br>Pelvic exenteration: 90450–00, 90450–01, 90450–02	-	Laparoscopic abdominal hysterectomy:<br>Abdominal hysterectomy: 68.3, 68.4, 68.6,<br>Vaginal hysterectomy: 68.5, 68.7<br>Pelvic exenteration: 68.8<br>Other and unspecified hysterectomy: 68.9	-
**Oophorectomy***	-	Laparoscopic partial oophorectomy: 35638-01<br>Unilateral: 35638-02,	-	Unilateral: 65.3, 65.4<br>Bilateral: 65.5, 65.6	-

For peer review only

		35713-07<br>Bilateral:<br>35638-03,<br>35717-01			
**Mastectomy***	MBS claims for<br>+ Total mastectomy: 31519 (unilateral), 31520 (bilateral).<br>+ Subcutaneous Mastectomy: 31524, 31522 (skin-sparing, unilateral), 31523 (skin-sparing, bilateral), 31528 (nipple-sparing, unilateral), 31529 (nipple-sparing, bilateral)	+ Simple mastectomy: 31518-00 (unilateral), 31518-01 (bilateral).<br>+ Subcutaneous mastectomy: 31524-00 (unilateral), 31524-01 (bilateral)	-	+ Unilateral: 85.33, 85.34, 85.41, 85.43, 85.45, 85.47<br>+ Bilateral: 85.35, 85.36, 85.42, 85.44, 85.46, 85.48 or any combination of 2 mastectomy codes<br>+ Prophylactic mastectomy - mostly bilateral mastectomy: V50.41	+ Unilateral: Z90.12, Z90.11<br>+ Bilateral: Z90.13
**Specialist/consulting physician attendances**	MBS claims for:<br>Other non-referred attendances (not GP): 52, 53, 54, 57, 58, 59, 60, 65, 92, 93, 95, 96<br>Specialist attendances: 99, 104, 105, 106, 107, 108, 109, 113<br>Consultant physician attendances: 110, 112, 114, 116, 119, 122, 128, 131, 132, 133<br>Prolonged attendances: 160, 161, 162, 163, 164<br>Urgent attendances after hours: 598, 600<br>Emergency physician attendance: 501, 503, 507, 511, 515, 519, 520, 530, 532, 534, 536<br>Consultant psychiatrist attendances: 288, 289, 291, 293, 296, 297, 299, 300, 302, 304, 306, 308, 310, 312, 314, 316, 318, 319, 320, 322, 324, 326, 328, 330, 332,	-	-	-	-

For peer review only

	334, 336, 338, 342, 344, 346, 348, 350, 352, 353, 355, 356, 357, 358, 359, 361, 364, 366, 367, 369, 370<br>Pain and palliative medicine: 2799, 2801, 2806, 2814, 2820, 2824, 2832, 2840, 2946, 2949, 2954, 2958, 2972, 2974, 2978, 2984, 2988, 2992, 2996, 3000, 3003, 3005, 3010, 3014, 3015, 3018, 3023, 3028, 3032, 3040, 3044, 3051, 3055, 3062, 3069, 3074, 3078, 3083, 3088, 3093<br>Geriatric medicine: 141, 143, 145, 147, 149				
**GP Attendances**	MBS claims for:<br>GP Health Assessment: 93470, 93479<br>GP Chronic Disease Management Plan: 229, 230, 231, 232, 233, 93469, 93475<br>GP Multidisciplinary Case Conference: 235, 236, 237, 238, 239, 240, 243, 244, 735, 739, 743, 747, 750, 758<br>Medication Management Review (domiciliary): 245, 900<br>Medication Management Review (residential): 249, 903.<br>GP Mental Health: 272, 276, 277, 279, 281, 282, 894, 896, 898, 941, 942, 2121, 2150, 2196, 90264, 90265, 92112, 92113, 92114, 92115, 92116, 92117, 92118, 92119, 92120, 92121, 92122, 92123, 92124, 92125, 92126, 92127, 92128, 92129, 92130, 92131, 92132, 92133, 92134, 92135, 92146, 92147, 92148, 92149, 92150, 92151, 92152, 92153, 92154, 92155, 92156, 92157, 92158, 92159, 92160, 92161, 92170, 92171, 92176, 92177, 92182, 92184, 92186, 92188, 92194, 92196, 92198, 92200<br>After-hours GP: 597-600 (urgent); 5000, 5020, 5040, 5060, 5200, 5203, 5207, 5208, 5003, 5010, 5023, 5028, 5043, 5049, 5063,	-	-	-	-

For peer review only

	5067, 5220, 5223, 5227, 5228, 5260, 5263, 5265, 5267, 733, 737, 741, 745, 761, 763, 766, 769, 772, 776, 788, 789 (non-urgent)<br>GP – Practice Incentive Program services: 251, 252, 253, 254, 255, 256, 257 (cervical smear); 259, 260, 261, 262, 263, 264 (diabetes mellitus annual cycle of care); 265, 266, 268, 269, 270, 271 (asthma cycle of care)<br>GP short (level A): 3, 4, 2095, 2461, 90020, 91790, 91795, 91890<br>GP Standard (Level B): 23, 24, 2144, 2463, 90035, 91800, 91809, 91891<br>GP Long (Level C): 36, 37, 2180, 2464, 90043, 91801, 91810, 91894<br>GP Prolonged (Level D): 44, 47, 2193, 2465, 90051, 91802, 91811<br>Other Non-referred Medical Practitioner attendances: 899, 901, 905, 906, 90002, 91792, 91794, 91797, 91799, 91803, 91804, 91805, 91806, 91807, 91808, 91812, 91813, 91814, 91815, 91816, 91817, 91892, 91895, 92716, 92717, 92719, 92720, 92722, 92723, 92725, 92726, 92732, 92733, 92735, 92736, 92738, 92739, 92741, 92742, 92747, 93660, 93661, 93681, 93682, 93684, 93685, 93691, 93692, 93694, 93695, 93701, 93702, 93704, 93705<br>GP Focussed Psychological Strategies and Family Group Therapy: 283, 285, 286, 287, 371, 372<br>GP Acupuncture: 173, 193, 195, 197, 199<br>GP Telehealth (patient-end support): 812, 827, 829, 867, 868, 869, 873, 876, 881, 885, 891, 892<br>GP attendances relating to residential aged care facilities: 232, 249, 731, 772, 776, 788, 789, 829, 869, 881, 892, 903, 2125,				

	2138, 2179, 2220, 5010, 5028, 5049, 5067, 5260, 5263, 5265, 5267, 92102, 92071, 92058, 92027				
**Invasive breast cancer**	-	-	-	+ Invasive breast cancer: 174.x	+ Invasive breast cancer: C50.xxx
**Non-invasive breast cancer**	-	-	-	+ Non-invasive breast cancer: 233.0	+ Non-invasive breast cancer: D05.0, D05.01, D05.02 (lobular carcinoma in situ), D05.10, D05.11, D05.12 (Intraductal carcinoma in situ), D05.80, D05.81, D05.82 (other specified type of carcinoma in situ, breast), D05.90, D05.91, D05.92 (unspecified type of carcinoma in situ of breast)
**Unspecified breast cancer**	-	-	-	+ Personal history of malignant neoplasm of breast: V10.3<br>+ Secondary malignant neoplasm of the breast (cancer metastatic to the breast): 198.81	+ Personal history of malignant neoplasm of breast: Z85.3<br>+ Secondary malignant neoplasm of the breast (cancer metastatic to the breast): C79.81
**Other cancers (excluding breast cancer and non-melanoma skin**	-	-	-	140 - 172, 175 - 208	C00–C49, C51-C97, D45, D46, D47.1, D47.3–D47.5

For peer review only

**cancers)**					
**Statins use**	-	-	+ HMG CoA reductase inhibitors: C10AA<br>+ Combinations of various lipid modifying agents: C10BA<br>+ Lipid modifying agents in combination with other drugs: C10BX	-	-
**Combined hormonal contraceptives**	-	-	+ Progestogens and estrogens, fixed combinations: G03AA<br>+ Progestogens and estrogens, sequential preparations: G03AB<br>+ Vaginal ring with progestogen and estrogen: G02BB01<br>+ Cyproterone and estrogen: G03HB01	-	-
**Combined menopausal hormone therapy**	-	-	+ Progestogens and estrogens in combinations: G03F<br>+ Vaginal ring with progestogen and estrogen: G02BB01<br>+ Cyproterone and estrogen: G03HB01<br>+ Combinations of ATC codes for estrogens only and progestogens only:<br>Estrogens: G03C<br>Progestogens: G03D,<br>Progestogen intrauterine device:	-	-

For peer review only

			G02BA03		
**Comorbidity (Rx- Risk score)**					
Alcohol dependency	-	-	Disulfiram, Naltrexone: N07BB01 - N07BB99	-	-
Allergies	-	-	Antihistamines (except hydroxyzine and diphenhydramine), nasal anti-inflammatories: R01AC01 -R01AD60, R06AD02 - R06AX27, R06AB04	-	-
Anticoagulants	-	-	Anticoagulants: B01AA03 - B01AB06, B01AE07, B01AF01, B01AF02, B01AX05	-	-
Antiplatelets	-	-	Antiplatelets: B01AC04 - B01AC30	-	-
Anxiety	-	-	Anxiolytics (benzodiazepines): N05BA01-N05BA12, N05BE01	-	-
Arrhythmias	-	-	Antiarrhythmics, Digoxin: C01AA05, C01BA01 - C01BD01, C07AA07	-	-
Benign prostatic hypertrophy	-	-	Alpha blockers: G04CA01-G04CA99, G04CB01, G04CB02 for men only		
Bipolar disorder	-	-	Lithium: N05AN01	-	-
Chronic airways disease	-	-	Inhaled bronchodilators: R03AC02-R03DC03,	-	-

For peer review only

			R03DX05		
Congestive heart failure	-	-	Loop diuretics AND, ACE or ARB; Betablockers selective for heart failure. Aldosterone antagonists specific for heart failure: C03DA02–C03DA99, C07AB02—if PBS item code is 8732N, 8733P, 8734Q, 8735R. C07AB07, C07AG02, C07AB12, C03DA04 (C03CA01–C03CC01 and C09AA01–C09AX99, C09CA01–C09CX99 †		
Dementia	-	-	Anticholinesterases or other dementia medicines: N06DA02-N06DA04, N06DX01	-	-
Depression	-	-	Antidepressants: N06AA01-N06AG02, N06AX03- N06AX11, N06AX13-N06AX18, N06AX21-N06AX26	-	-
Diabetes			Insulins, oral Hypoglycaemics: A10AA01–A10BX99		
Epilepsy	-	-	Anticonvulsants: N03AA01-N03AX99	-	-
Glaucoma	-	-	Topical antiglaucoma Agents: S01EA01-S01EB03,	-	-

For peer review only

			S01EC03-S01EX99		
Gastric acid disorder	-	-	Histamine H2-receptor antagonists, Proton-pump inhibitor: A02BA01-A02BX05	-	-
Gout	-	-	Antigout agents: M04AA01-M04AC01	-	-
Hepatitis B	-	-	Anti Hep B antivirals: J05AF08, J05AF10, J05AF11	-	-
Hepatitis C	-	-	Interferon/ribavirin combinations, Protease inhibitors for hepatitis: J05AB54, J05AE11-J05AE12, J05AE14, J05AX14, J05AX15, J05AX65, J05AB04 L03AB10, L03AB11, L03AB60, L03AB61	-	-
HIV	-	-	Anti-HIV antivirals: J05AE01-J05AE10, J05AF01-J05AF07, J05AF09, J05AF12-J05AG05, J05AR01-J05AR99, J05AX07-J05AX09 J05AX12	-	-
Hyperkalaemia	-	-	Sodium polystyrene Sulfonate: V03AE01	-	-
Hyperlipidaemia	-	-	Antilipaemic agents: C10AA01-C10BX09, A10BH03‡		
Hypertension	-	-	Thiazides, potassium sparing agents,		

For peer review only

			combination antihypertensives, other antihypertensives: C03AA01–C03BA11, C03DB01- C03DB99, C03EA01, C09BA02–C09BA09, C09DA02–C09DA08, C02AB01–C02AC05, C02DB02–C02DB99 (C03CA01–C03CCO1 or C09CA01–C09CX99)§		
Hyperthyroidism	-	-	Antithyroid Preparations: H03BA02, H03BB01	-	-
Hypothyroidism	-	-	Thyroid replacements: H03AA01-H03AA02	-	-
Irritable bowel syndrome	-	-	IBS specific drugs, rectal anti-inflammatories: A07EC01–A07EC04, A07EA01–A07EA02, A07EA06, L04AA33	-	-
Ischemic heart disease: Angina	-	-	Nitrates: C01DA02-C01DA14, C01DX16, C08EX02		
Ischemic heart disease: Hypertension			C07AA01–C07AA06, C07AA08–C07AB01, C07AB02—if PBS item code is not 8732N, 8733P, 8734Q, 8735R, C07AG01, C08CA01–C08DB01, C09DB01–C09DB04, C09DX01,		

For peer review only

			C09BB02–C09BB10, C07AB03, C09DX03, C10BX03¶		
Incontinence	-	-	G04BD01–G04BD99		
Inflammation/pain	-	-	NSAIDS: M01AB01-M01AH06	-	-
Liver failure	-	-	Rifaximin: A06AD11, A07AA11	-	-
Malignancies	-	-	Antineoplastics agents (excluding topical): L01AA01-L01XX41	-	-
Malnutrition	-	-	Enteral nutritional Supplements: B05BA01-B05BA10	-	-
Migraine	-	-	Antimigraine Medications: N02CA01-N02CX01	-	-
Osteoporosis/Paget's	-	-	Alendronate, etidronate: M05BA01-M05BB05, M05BX03, M05BX04, G03XC01, H05AA02	-	-
Pain	-	-	Opiate containing Medications: N02AA01–N02AX02, N02AX06, N02AX52, N02BE51		
Pancreatic insufficiency	-	-	Pancreatic exocrine enzyme replacement: A09AA02	-	-
Parkinsons disease	-	-	Antiparkinson agents: N04AA01-N04BX02	-	-
Psoriasis	-	-	Systemic and topical antipsoriatics: D05AA01-D05AA99, D05BB01-D05BB02, D05AX02,	-	-

For peer review only

			D05AC01-D05AC51, D05AX52		
Psychotic illness	-	-	Antipsychotics: N05AA01-N05AB02, N05AB06-N05AL07, N05AX07-N05AX13	-	-
Pulmonary hypertension	-	-	Antihypertensives for pulmonary arterial hypertension: C02KX01–C02KX05, PBS item code 9547L, 9605M	-	-
Renal disease	-	-	Alpha erythropoietin, calciferol, calcitriol, sevelamer: B03XA01-B03XA03, A11CC01-A11CC04, V03AE02, V03AE03, V03AE05	-	-
Smoking cessation	-	-	Nicotine, Bupropion: N07BA01-N07BA03, N06AX12	-	-
Steroid-responsive conditions	-	-	Glucocorticoids: H02AB01-H02AB10	-	-
Transplant	-	-	Immune suppressants: L04AA06, L04AA10, L04AA18, L04AD01, L04AD02	-	-
Tuberculosis	-	-	Anti-tubercular agents: J04AC01 - J04AC51, J04AM01 -J04AM99	-	-

MBS: Medicare Benefits Schedule, PBS: Pharmaceutical Benefits Scheme, AM: Australia Modification, ICD: International Classification of Diseases, FNA: Fine-needle aspiration, ACHI: The Australian Classification of Health Interventions. Notes: MBS codes and procedure codes are exclusively used for Australian data. *: Procedures conducted out of hospital or in private hospitals are recorded in MBS data by MBS item number whereas those conducted in public hospitals are captured in the hospital admission data by procedure codes. †: Must have at least two medicines prescribed with one of those medicines having an ATC code from C03CA01–C03CC01 and the other having an ATC code from either C09AA01–C09AX99 or C09CA01–C09CX99. ‡: Combination product for hyperlipidaemia and diabetes. §: Can have medicine dispensed

For peer review only

with an ATC code C03CA01–C03CC01 or C09AA01–C09AX99, but not both, as this would indicate chronic heart failure. ¶: Combination product for hyperlipidaemia and ischaemic heart disease: hypertension

For peer review only

Title: Association between long-term use of calcium channel blockers (CCB) and the risk of breast cancer: A retrospective longitudinal observational study protocol

Appendix Table 3. Description and response categories of data items from baseline surveys of the ALSWH Study, the 45 and Up Study and Rotterdam Study

Table A. The Australian Longitudinal Study on Women's Health Study (ALSWH)

Description of data item	Question	Response categories
**Socio-demographic characteristics**		
Age	BOTH COHORTS: What is your date of birth?	Date variable (DD/MM/YYY)
Marital status	BOTH COHORTS: What is your present marital status?	+ 1946-51 COHORT: Married; de facto (in a relationship); widowed; separated; divorced; never married<br>+ 1921-26 COHORT: Married (registered); de facto relationship (opposite sex); de facto relationship (same sex); separated; divorced; widowed; never married
Country of birth	BOTH COHORTS: In which country were you born?	BOTH COHORTS: Australia; UK; Ireland; Italy; Greece; New Zealand; Vietnam; other (please specify)
	BOTH COHORTS: If you were not born here, when did you first arrive in Australia with the intention of living here for one year or more?	+ 1946-51 COHORT: 1955 or earlier; 1956-1965; 1966-1985; 1986-1990; 1991 or later<br>+ 1921-26 COHORT: 1935 or earlier; 1936-1945; 1946-1955; 1956-1965; 1966-1985; 1986-1990; 1991 or later
Education status	BOTH COHORTS: What is the highest qualification you have completed?	BOTH COHORTS: No formal qualifications; school or intermediate certificate (or equivalent); higher school or leaving certificate (or equivalent); trade/apprenticeship (e.g. hairdresser, chef); certificate/diploma (e.g. child care, technician); university degree; higher university degree (e.g. Grad Dip, Masters, PhD)
**Lifestyle characteristics**		
Body mass index (height/weight)	BOTH COHORTS: How tall are you without shoes?	Continuous variable (height in cm)
	BOTH COHORTS: How much do you weigh without clothes or shoes?	Continuous variable (weight in kg)

For peer review only

Description of data item	Question	Response categories
Physical activity	BOTH COHORTS:<br>If you add up all the time you spent doing each activity LAST WEEK, how much time did you spend ALTOGETHER doing each type of activity?<br>1. Walking briskly (for recreation or exercise or to get to or from places)<br>2. Moderate leisure activity (like social tennis, moderate exercise classes, recreational swimming, dancing)<br>3. Vigorous leisure activity (that make you breathe harder or puff and pant like aerobics, competitive sport, vigorous cycling, running, swimming)<br>3. Vigorous household or garden chores (that make you breathe harder or puff and pant)	Continuous variable (time in hours/minutes)
Smoking status	1946-51 COHORT: How often do you currently smoke cigarettes or any tobacco products?	1946-51 COHORT: Daily; at least weekly (but not daily); less often than weekly; not at all
Alcohol drinks per week	1946-51 COHORT: How often do you usually drink alcohol?	1946-51 COHORT: I never drink alcohol; I drink rarely; less than once a week; on 1 or 2 days a week, on 3 or 4 days a week; on 5 or 6 days a week; every day
	1946-51 COHORT: On a day, when you drink alcohol, how many drinks do you usually have	1946-51 COHORT: 1 or 2 drinks per day; 3 or 4 drinks per day; 5 to 8 drinks per day; 9 or more drinks per day
Shift work at night	1946-51 COHORT: Do you normally do paid work at night?	1946-51 COHORT: Yes; no
**Clinical characteristics**		

For peer review only

Description of data item	Question	Response categories
High BP	BOTH COHORTS: Have you ever been told by a doctor that you have hypertension (high blood pressure)?	BOTH COHORTS: Yes; no
	BOTH COHORTS: In the past 3 years, have you been diagnosed or treated for high blood pressure?	BOTH COHORTS: Yes; no
Diabetes	BOTH COHORTS: Have you ever been told by a doctor that you have diabetes?	BOTH COHORTS: Yes; no
	BOTH COHORTS: In the past 3 years, have you been diagnosed or treated for diabetes (high blood sugar)?	BOTH COHORTS: Yes; no
	1946-51 COHORT: Have you ever been told by a doctor that you have non-insulin dependent (type 2) diabetes?	1946-51 COHORT: Yes; no
	1946-51 COHORT: In the past 3 years, have you been diagnosed or treated for non-insulin dependent (type 2) diabetes?	1946-51 COHORT: Yes; no
	1921-26 COHORT: In the last 3 years, have you been told by a doctor that you have diabetes?	1921-26 COHORT: Yes; no
Heart disease	BOTH COHORTs: Have you ever been told by a doctor that you have heart disease?	BOTH COHORTs: Yes; no
	1946-51 COHORT: In the past 3 years, have you been diagnosed or treated for heart disease?	1946-51 COHORT: Yes; no
	1921-26 COHORT: In the past 3 years, have you been diagnosed or treated for angina/heart attack/other heart problems?	1921-26 COHORT: Yes; no
Stroke	BOTH COHORTS: Have you ever been told by a doctor that you have stroke?	BOTH COHORTS: Yes; no

For peer review only

Description of data item	Question	Response categories
	BOTH COHORTS: In the past 3 years, have you been diagnosed or treated for stroke?	BOTH COHORTS: Yes; no
	1921-26 COHORT: In the last 3 years, have you been told by a doctor that you have stroke	1921-26 COHORT: Yes; no
**Reproductive health**		
Age at menarche	1946-51 COHORT: At what age did your period start?	Continuous variable (Age in years)
Age at menopause	1946-51 COHORT: Have you had a period or menstrual bleeding in the last 12 months?	1946-51 COHORT: Yes; no
	1946-51 COHORT: If you have reached menopause, at what age did your periods completely stop?	Continuous variable (Age in years)
Age when had first child	1946-51 COHORT: If you have given birth, please write the year of the birth in the birth (if you had twins, please write the date twice)	Continuous variable (Year)
Number of children	1946-51 COHORT: If you have given birth, please write the year of the birth in the birth (if you had twins, please write the date twice)	Continuous variable (Year)
History of hysterectomy/ both ovaries removed	BOTH COHORTS: Have you ever had hysterectomy?	BOTH COHORTS: Yes; no
	BOTH COHORTS: In the PAST THREE YEARS, have you had hysterectomy?	BOTH COHORTS: Yes; no
	BOTH COHORTS: Have you ever had both ovaries removed?	BOTH COHORTS: Yes; no
	BOTH COHORTS: In the PAST THREE YEARS, have you had both ovaries removed?	BOTH COHORTS: Yes; no

For peer review only

Description of data item	Question	Response categories
**Healthcare utilisation**		
Concession card holders	1946-51 COHORT: Do you have a Health Care Card? (This is a card that entitles you to discounts and assistance with medical expenses. This is not the same as a Medicare card)	1946-51 COHORT: Yes; no
Mammogram	1946-51 COHORT: When did you last have a mammogram?	1946-51 COHORT: I have never had a mammogram; 2 years ago or less; more than 2 but less than 5 years ago; 5 or more years ago; not sure
	1946-51 COHORT: Have you ever had an abnormal mammogram?	1946-51 COHORT: No; Yes in the last 5 years; Yes more than 5 years ago
	1946-51 COHORT: In the last two years, have you had a mammogram?	1946-51 COHORT: Yes; no
**Diagnosis of cancer**		
A diagnosis/history of breast cancer	BOTH COHORT: Have you ever been told by a doctor that you have breast cancer?	BOTH COHORT: Yes; no
	1946-51 COHORT: In the past 3 years, have you been diagnosed or treated for breast cancer?	1946-51 COHORT: Yes; no
	1921-26 COHORT: In the last 3 years, have you been told by a doctor that you have breast cancer?	1921-26 COHORT: Yes; no
**Medicine use**		
Current use of hormonal contraception	1946-51 COHORT: Are you currently taking the oral contraceptive pill for any reason?	1946-51 COHORT: Yes; no
Current use of menopausal hormone therapy	1946-51 COHORT: Are you currently on hormone replacement therapy (HRT)?	1946-51 COHORT: Yes; no

BP: blood pressure

For peer review only

Table B. The 45 and Up Study

Description of data item	Question	Response categories
**Socio-demographic characteristics**		
Age	What is your date of birth?<br>What is today's date?	Date variable (DD/MM/YYYY)
Marital status	What best describes your current situation?	Single; married; de facto/living with a partner; widowed; divorced; separated
Country of birth	In which country were you born?	Australia; UK; Ireland; Italy; China; Greece; New Zealand; Germany; Lebanon; Philippines; Netherlands; Vietnam; Malta; Poland; other (please specify)
	What year did you first come to live in Australia for one year or more?	Continuous variable (Year)
Work status	What is your current work status? (you can cross more than one box)	In full time paid work; self-employed; in part time paid work; doing unpaid work; completely retired/pensioner; studying; partially retired; looking after home/family; disabled/sick; unemployed; other
	If you are partially or completely retired, how old were you when you retired?	Continuous variable (Age in years)
	Why did you retire?	Reached usual retirement age; lifestyle reasons; to care for family member/friend; ill health; made redundant; could not find a job; other
Education status	What is the highest qualification you have completed?	No school certificate or other qualifications; school or intermediate certificate (or equivalent); higher school or leaving certificate or equivalent); trade/apprenticeship (e.g. hairdresser, chef); certificate/diploma (e.g. child care, technician); university degree or higher
**Lifestyle characteristics**		
Body mass index (height/weight)	How tall are you without shoes?	Continuous variable (Centimetres, Feet, Inches)
	About how much do you weigh?	Continuous variable (Kilograms; Stone; Pounds)
Physical activity	If you add up all the time you spent doing each activity LAST WEEK, how much time	Walking continuously; Vigorous physical activity; Moderate physical activity

For peer review only

Description of data item	Question	Response categories
	did you spend ALTOGETHER doing each type of activity?	
Smoking status	Have you ever been a regular smoker?	Yes; no
	Are you a regular smoker now?	Yes; no
Alcohol drinks per week	About how many alcoholic drinks do you have each week?<br>On how many days each week do you usually drink alcohol?	Continuous variable (Integer)
**Clinical characteristics**		
High BP/ Diabetes/ Heart Disease/ Stroke/ Breast cancer/ Other cancer	Has a doctor EVER told you that you have:	Skin cancer (not melanoma); Melanoma; Breast cancer (F); Prostate cancer (M); Other cancer (free text); Heart disease (free text); High blood pressure - when pregnant (F); High blood pressure - when not pregnant (F); High blood pressure (M); Stroke; Diabetes; Blood clot (thrombosis); Enlarged prostate (M); Asthma; Hay fever; Depression; Anxiety; Parkinson's disease; None of these
Family history of heart disease/ stroke/ breast cancer/ ovarian cancer	Have your mother, father, brother(s) or sister(s) ever had:	Heart disease; High blood pressure; Stroke; Diabetes; Dementia/Alzheimer's; Parkinson's disease; Severe depression; Severe arthritis; Breast cancer; Bowel cancer; Lung; Melanoma; Prostate cancer; Ovarian cancer; Osteoporosis; Hip fracture; Do not know
**Reproductive health**		
Age at menopause	Have you been through menopause?	No; not sure (because hysterectomy, taking HRT, etc.); my periods have become irregular; yes
	If yes, how old were you when you went through menopause?	Continuous variable (Age in years)
Age when had first child	How old were you when you gave birth to your FIRST child?	Continuous variable (Age in years)
Number of children	How many children have you given birth to?	Continuous variable (Age in years)
History of hysterectomy/ both ovaries removed	Have you ever had hysterectomy?	Yes; no
	Have you ever had both ovaries removed?	Yes; no

For peer review only

Description of data item	Question	Response categories
Breastfeeding	For how many months, in total, have you breastfed?	Continuous variable (number of months)
**Healthcare utilisation**		
Concession card holders	Which of the following do you have? (excluding Medicare)	Private health insurance-with extra; private health insurance – without extras; Department of Veterans' Affairs white or gold card; Health care concession card; none of these.
Mammogram	Have you ever been for a breast screening mammogram?	Yes; no
	If Yes, what year did you have your last mammogram?	Continuous variable (Year)
	How many times have you been for breast screening altogether?	Continuous variable (Integer)
**Medicine use**		
Current use of hormonal contraception	Have you ever used the pill or other hormonal contraceptives? (e.g. the combined pill, mini pill, contraceptive implant or injections)	Yes; no
	If Yes, how old were you when you LAST used hormonal contraceptives? (please write your current age if you are still using them)	Continuous variable (Age in years)
Current use of menopausal hormone therapy	Have you ever used hormone replacement therapy (HRT)?	Yes; no
	Are you currently taking HRT?	Yes; no

F: female, M: male, BP: blood pressure, HRT: hormone replacement therapy

For peer review only

Table C. Rotterdam Study

Description of data item	Question	Response categories
**Socio-demographic characteristics**		
Age	Date of birth was provided by the municipality, and age was subsequently computed by data management	Continuous variable (Age in years)
Marital status	*Home interview:* What is your marital status?	Never been married; married (once); widowed, then remarried; divorced and remarried; never been married, lives with unmarried spouse; widowed, lives with unmarried spouse; divorced, lives with unmarried spouse; widowed; divorced
Work status	*Home interview:* What is your current employment status?	Employed; unemployed, out of job; house wife/man; unfit for work; rentier; early retirement; retirement
Education status	*Home interview:* What is your highest attained education?	Primary education; lower vocational education; intermediate secondary education; intermediate vocational education; general secondary education; higher education (Higher professional education); higher education (University)
**Lifestyle characteristics**		
Body mass index (height/weight)	*Clinical examination:* Weight was assessed with the participant wearing light indoor clothes, without shoes. Height was measured.	Weight in kilograms, height in centimetres
Smoking status	*Home interview:* Do you smoke cigarettes?	No; yes; don't know; no answer
	Did you quit smoking ever?	No; yes; don't know; not applicable; no answer
	Did you use to smoke cigarettes earlier?	No; yes; don't know; not applicable; no answer
	Did you smoke cigars? Did you ever smoke cigars or did you smoke cigars but quitted now?	No, never; ever little cigars; ever big cigars; ever both; yes little; yes big; yes both; don't know; no answer
Alcohol drinks per week	How many glasses of beer/wine/spirit do you drink a day?	Continuous variable (Integer)

For peer review only

Description of data item	Question	Response categories
**Clinical characteristics**		
High BP	Clinical exam: Hypertension	No; Yes;
Diabetes	Clinical exam: Diabetes	No; Yes;
Systolic BP	Clinical exam: Systolic BP	Continuous variable
Diastolic BP	Clinical exam: Diastolic BP	Continuous variable
Blood cholesterol level	Clinical exam: Blood drawn, clinical lab.	Continuous variable (Value)
Family history of heart disease	*Home interview:* Did one of your parents or siblings experience a heart attack?	No; yes
**Reproductive health**		
Age at menarche	*Home interview:* At what age did your period start?	Continuous variable (Age in years)
Age at menopause	*Home interview:* At what age had your periods stopped definitively?	Continuous variable (Age in years)
Age when had first child	*Home interview:* In what year was your first child born?	Continuous variable (Age in years, calculated by data management team)
Number of children	*Home interview:* How many pregnancies (of at least 6 months) have you had?	Continuous variable (Integer)
History of hysterectomy	*Home interview:* Tick box, yes no	No; Yes
History of both ovaries removed	*Home interview:* Tick box, yes no	No; Yes

BP: blood pressure. Clinical exams were undertaken via automated coupling of the study database with medical records from general practitioners.

Standard Protocol Items for Observational Studies (SPIROS)

Table 1. SPIROS 2023 Checklist: Recommended Items to address in the observational study Protocol and related documents

Section/ item	Description	Addressed on page number
**Part A: general information**		
Title	Descriptive title Identifying study design in the title	Page 1
Protocol version	Version or amendment number with date and summary of the changes	Document name
Protocol summary	An informative and balanced summary of the study protocol	Page 2
Sponsor and funder details	Name of Sponsor and funder and types of financial, material, and other support	Page 26
Conflict of interest statements	Statement about any financial and other competing interests for principal or co investigators for the overall study.	Page 26
Investigators name	Names of the principal and co-investigators	Page 1
Affiliation of investigators	Affiliated institutions of the investigators	Page 1
Principal researcher/s contact detail	Name, e-mail address, affiliation of principal researcher	Page 1
**Part B: Introduction**		
Background of the study	Description of research question and scientific background of the study	Page 4
Review of prior research	Summary of relevant existing research (published or unpublished)	Page 4-6
Rationale of study	Justification for conducting the study	Page 5-6
Aim	Broader aims and overall objective	Page 6
Objective/s of the study	Primary and secondary objective/s including any prespecified hypothesis (if applicable).	Page 6
	Specify whether the intention is to (a) estimate causal effects, (b) predict outcomes, or (c) simple description.	Page 6
**Part C: Methods**		
Study design	Description of study design (case control, cross-sectional or cohort) and type of study (retrospective cohort study, Prospective cohort study etc)	Page 7
Study setting	Description of the study setting (e.g., community-based, hospital based) and detail of precise locations of the study sites.	NA

For peer review only

Study schedule	Description of the expected schedule of the study including relevant dates, expected periods of recruitment/survey, exposure, follow-up, and data collection.	Page 8-9
	Figure (Study schematic/flow-chart) or table describing expected time frame for each step including trainings, data collection, follow-up, analysis and reporting etc.	Figure 1
Sample size	Estimation of minimum sample size required for the study with justifications including clinical and statistical assumptions supporting any sample size calculations.	Page 16, figure 3
Sampling procedure	Detailed description of the sampling frame and sampling strategy (simple random, stratified random, cluster, systematic etc.)	NA
Participant selection		
Participant selection for cohort study	Description of inclusion and exclusion criteria, and the source and methods of participant selection (exposed and unexposed). For matched cohort studies, give matching criteria and number of exposed and unexposed.	Page 6-9
Participant selection for case-control study	Description of inclusion and exclusion criteria, and the source and methods of case ascertainment and control selection. Give the rationale for the choice of cases and controls. Give diagnostic criteria for identifying cases (if applicable). For matched case-control studies, give matching criteria and the number of controls per case.	NA
Participant selection for cross-sectional study	Description of the inclusion and exclusion criteria, and the source and methods of participant selection.	NA
Variables	Detailed description of all important baseline and outcome variables to be analysed, exposures, predictors, potential confounders, and effect modifiers. Give diagnostic criteria, if applicable.	Figure 2, appendix table 1-2
Data sources/measurement	For each variable of interest, give sources of data and details of assessment /measurement methods. Describe comparability of assessment methods if there is more than one group.	Table 1
Data collection and management	Plans for assessment and collection of outcomes, baseline, follow up and other study related data.	Page 10-13
	Description of data collection methods e.g., online survey, Household survey, paper based or electronic data capture etc.	Page 10-13
	Any related processes to promote data quality during data	NA

For peer review only

	collection (e.g., duplicate measurements, training of assessors, validation method)	
	Description of study instruments (e.g., questionnaires, data collection forms) along with their reliability and validity, if known. Reference to where data collection forms can be found, if not in the protocol.	Appendix table 3
	Plans for data entry, coding, security, and storage, including any related processes to promote data quality (e.g., electronic data capture, double data entry; range checks for data values, random cross-checking of electronic data with the source documents).	NA
	Reference to where details of data management procedures can be found, if not in the protocol.	NA
Blinding procedure (if blinded study)	Description of blinding procedure (if applicable) reporting Who will be blinded (e.g., investigator blinded for disease status when measuring exposure in case-control study) and methods to ensure blinding and unmasking of blinding if required.	NA
Potential bias	Description of any potential biases and plan to minimize those potential sources of biases.	Page 19-20
Statistical analysis plan	Detailed description of methods for analysing and presenting primary/secondary outcomes and any additional analysis (e.g. analyses of subgroups and interactions, and sensitivity analyses). Give reference to the where other details of the statistical analysis plan can be found, if not in the protocol.	Page 14-16
Handling of missing data	Detailed description of methods to handle missing data (e.g. multiple imputation).	NA
Handling of withdrawals and lost to follow up	Detailed description of the procedures to be followed when a participant ceases participation in the study prematurely or is lost to follow up	Page 9
Replacements	Plans and methods of the replacement or substitution of withdrawn participants.	NA
Outcome	Definition and description of all primary, secondary and other outcomes.	Page 12-13
Data confidentiality statement	A detailed description of process to ensure data confidentiality.	NA
Follow up	A detailed plan of follow up including schedule and methods (telephonic, house based, hospital based etc.) of follow up.	NA

For peer review only

Plan of study monitoring	Description of plan for study monitoring and whether the monitoring will be independent from investigators or sponsors.	NA
Training of surveyors/data collectors	Description of how investigators and surveyors will be trained to conduct the research activity.	NA
Quality assurance	Plan of quality assurance. Back-checking data collection.	NA
**Part D: Ethical consideration**		
Ethical approval	Plan for seeking ethics approval from ethics committees/institutional review boards. If known, give name of ethical committees.	Page 16-17
	If ethics approval will not be sought, give justification.	NA
Consent and assent	Description of who will obtain informed consent or assent from potential study participants or authorized surrogates, and how (e.g., written informed consent, verbal consent, video/audio recording of consent procedure etc.)	Page 17
	Give reason if consent or assent not sought.	NA
	Give reference to where informed consent forms and applicable translations plan can be found, if not in the protocol.	NA
Risk/harm to participants	A detailed description of potential risks or harms to study participants.	NA
	Plans for collecting, assessing, reporting, and managing any study procedures related adverse events (e.g. adverse events due to blood collection) and other unintended effects of study conduct (e.g. risk to breach confidential and sensitive information of participants)	NA
	Give a statement about whether data will be anonymous, pseudonymized, or can be directly linked to participants.	NA
	Description of any plan for giving Incentives to the participants	NA
Adverse event and serious adverse event reporting	Outline how adverse events and serious adverse events information will be collected and reported.	NA
Involvement of patient/participant representatives in protocol development	Patient and Public Involvement (PPI) statement including how patients or participants involved in the planning of the study. Give statement, if there is no plan to involve of patient/participants and public in designing or any phase of the study	Page 16
**Part E: Reporting and dissemination**		

For peer review only

Dissemination/ publication plan	Plans for investigators and sponsor to communicate study results to ethical review boards, participants, key stake holders, the public, and other relevant groups.	Page 17
	Methods to communicate findings (e.g., via publication (open access or closed access), reporting in results databases, or other data-sharing arrangements), including any publication restrictions.	Page 17
	Define authorship eligibility guidelines (e.g., ICMJE recommendations)	Page 26
**Part F: Others**		
Whether Artificial Intelligence (AI) assisted technology was used in writing the protocol	Disclose whether authors used artificial intelligence (AI)- assisted technologies in the production of protocol (e.g., chatbots) or there is planning to use artificial intelligence (AI)- assisted technologies in the production of manuscript or study reports.	NA
	Give the name of AI tools (such as ChatGPT). Include a statement if authors did or did not review and edited the content created by AI-assisted technologies	NA
References	A complete list of references cited in protocol	Page 21-25
Funding	Source of any funding for the study and the role of the funders for the study	Page 26
Open science	Registration of observational study: Study identifier and registry name (e.g., open science framework, ClinicalTrials.gov, ICTRP or any other national or international study registry platform). If not yet registered, name of intended registry.	Page 2
	Data sharing: Plans, if any, for granting public access to the (1) full protocol and amendments, (2) participant-level data set, (3) Statistical analysis plan, (4) statistical codes and other study material (e.g., case report forms, study questionnaires and Informed consent forms). Give reference to where these documents can be found, if not included as annex in the protocol.	Page 27

For peer review only

BMJ Open

Association between long-term use of calcium channel blockers (CCB) and the risk of breast cancer: A retrospective longitudinal observational study protocol

Journal:	*BMJ Open*
Manuscript ID	bmjopen-2023-080982.R1
Article Type:	Protocol
Date Submitted by the Author:	24-Jan-2024
Complete List of Authors:	Ho, Chau; Curtin University, School of Population Health, Faculty of Health Sciences<br>Ha, Thi Ninh; Curtin University, School of Population Health, Faculty of Health Sciences<br>Youens, David; Curtin University, School of Population Health, Faculty of Health Sciences; The University of Western Australia, Cardiovascular Epidemiology Research Centre, School of Population and Global Health<br>Abhayaratna, Walter P.; Canberra health services; Australian National University, School of Medicine and Psychology<br>Bulsara, Max; The University of Notre Dame Australia, Institute for Health Research<br>Hughes, Jeffery; Curtin University, Curtin Medical School; PainChek Ltd<br>Mishra, Gita; The University of Queensland, School of Public Health<br>Pearson, Sallie-Anne; University of New South Wales, Medicines Policy Research Unit; The NNHMRC Medicines Intelligence Centre of Research Excellence<br>Preen, David; University of Western Australia, School of Population and Global Health; The NHMRC Medicines Intelligence Centre of Research Excellence<br>Reid, Christopher; Curtin University, School of Population Health, Faculty of Health Sciences<br>Ruiter, Rikje; Erasmus MC – University Medical Center, Department of Epidemiology; Maasstad Hospital, Department of Internal Medicine<br>Saunders, Christobel M; The University of Western Australia, Medical School; The University of Melbourne, Melbourne Medical School<br>Stricker, Bruno; Erasmus MC – University Medical Center, Department of Epidemiology<br>van Rooij, Frank J.A; Erasmus MC – University Medical Center, Department of Epidemiology<br>Wright, Cameron; Curtin University, School of Population Health, Faculty of Health Sciences; The University of Western Australia, Medical School<br>Moorin, Rachael; Curtin University, School of Population Health, Faculty of Health Sciences; University of Western Australia, School of Population and Global Health
**Primary Subject Heading**:	Oncology
Secondary Subject Heading:	Cardiovascular medicine

Keywords:	Breast tumours < ONCOLOGY, Hypertension < CARDIOLOGY, EPIDEMIOLOGY

SCHOLARONE™
Manuscripts

Title: Association between long-term use of calcium channel blockers (CCB) and the risk of breast cancer: A retrospective longitudinal observational study protocol

Authors: Chau LB Ho[1], Ninh Thi Ha[1], David Youens[1,2], Walter P. Abhayaratna[3,4], Max K. Bulsara[5], Jeff Hughes[6,7], Gita D Mishra[8], Sallie-Anne Pearson[9, 10], David B Preen[10,11], Christopher M Reid[1], Rikje Ruiter[12, 13], Christobel M Saunders[14,15,16], Bruno H Stricker[12], Frank J.A van Rooij[12], Cameron Wright[1, 14,17,18,] Rachael Moorin[1,11]

Affiliations:

[1]School of Population Health, Faculty of Health Sciences, Curtin University, WA, Australia, [2] Cardiovascular Epidemiology Research Centre, School of Population and Global Health, The University of Western Australia, WA, Australia, [3] Canberra Health Services, ACT, Australia, [4] School of Medicine and Psychology, Australian National University, ACT, Australia, [5] Institute for Health Research, The University of Notre Dame Australia, WA, Australia, [6]Curtin Medical School, Curtin University, WA, Australia, [7] PainChek Ltd, Sydney, NSW, Australia, [8] School of Public Health, The University of Queensland, Brisbane, QLD, Australia, [9] School of Population Health, University of New South Wales, Sydney, Australia, [10] The NHMRC Medicines Intelligence Centre of Research Excellence, [11] School of Population and Global Health, The University of Western Australia, WA, Australia, [12] Department of Epidemiology, Erasmus MC – University Medical Center, Rotterdam, the Netherlands, [13] Department of Internal Medicine, Maasstad Hospital, Rotterdam, the Netherlands, [14] Medical School, The University of Western Australia, WA, Australia, [15] Melbourne Medical School, The University of Melbourne, Victoria, Australia, [16] The Royal Melbourne Hospital, Victoria, Australia, [17] Fiona Stanley Hospital, WA, Australia, [18] School of Medicine, University of Tasmania, TAS, Australia

Corresponding author: David Youens. School of Population Health, Faculty of Health Sciences, Curtin University, Kent St, Bentley WA 6102, Australia. david.youens@curtin.edu.au.

For peer review only

Abstract

Introduction: Calcium channel blockers (CCB), a commonly prescribed antihypertensive (AHT) medicine, may be associated with increased risk of breast cancer. The proposed study aims to examine whether long-term CCB use is associated with the development of breast cancer and to characterise the dose-response nature of any identified association, to inform future hypertension management.

Methods and analysis: The study will use data from two of Australia's largest cohort studies; the Australian Longitudinal Study on Women's Health, and the 45 and Up Study, combined with the Rotterdam Study. Eligible women will be those with diagnosed hypertension, no history of breast cancer and no prior CCB use at start of follow-up (2004 to 2009). Cumulative dose-duration exposure to CCB and other AHT medicines will be captured at the earliest date of: the outcome (a diagnosis of invasive breast cancer); a competing risk event (e.g., bilateral mastectomy without a diagnosis of breast cancer, death prior to any diagnosis of breast cancer) or end of follow-up (censoring event). Fine and Gray competing risks regression will be used to assess the association between CCB use and development of breast cancer using a generalised propensity score to adjust for baseline covariates. Time-varying covariates related to interaction with health services will also be included in the model. Data will be harmonised across cohorts to achieve identical protocols and a two-step random effects individual patient-level meta-analysis will be used.

Ethics and dissemination: Ethical approval was obtained from the following Human research Ethics Committees: Curtin University (HRECs) (ref No. HRE2022-0335), NSW Population and Health Services Research Ethics Committee (2022/ETH01392 / 2022.31), ACT Research Ethics and Governance Office approval under National Mutual Acceptance for multi-jurisdictional data linkage research (2022.STE.00208). Results of the proposed study will be published in high-impact journals and presented at key scientific meetings.

Study Registration: ClinicalTrials.gov Identifier NCT05972785.

For peer review only

For peer review only - http://bmjopen.bmj.com/site/about/guidelines.xhtml

Strengths and limitations of this study

- This study will characterise any dose-response nature of the association between CCB use and breast cancer.
- To minimise the impact of confounding by indication, the study will be limited to participants with diagnosed/self-reported hypertension and comparisons will include participants exposed to other prescribed (non-CCB) AHT medicines in addition to those who have no pharmacotherapy for hypertension.
- Complete ascertainment of prior exposure will not be possible since we will not have lifetime capture of prescribing data, therefore CCB exposure may be under-reported.
- The diagnostic interval of breast cancer may vary between Australian and Dutch women, particularly since case ascertainment through cancer registries can take months from the initial diagnosis to coding notifications.

For peer review only

For peer review only - http://bmjopen.bmj.com/site/about/guidelines.xhtml

Introduction

Breast cancer has overtaken lung cancer as the most common cancer and the leading cause of death in women globally, accounting for two million new cases and 685,000 deaths worldwide in 2020 [1]. By 2040, breast cancer is expected to increase by 40% to more than three million new cases and one million deaths annually [2]. Breast cancer places a significant burden of disease, excess mortality and reduced quality of life on women and the broader health system and identifying and mitigating potentially modifiable risk factors is a critical strategy in reducing this burden.

Hypertension is a well-established, modifiable risk factor for stroke and other cardiovascular events and a major contributor to chronic diseases such as heart failure, chronic kidney disease, and retinopathy [3]. Worldwide, hypertension causes more deaths and morbidity than any other biomedical risk factor [4]. Cardiovascular disease is responsible for approximately 17 million deaths a year, nearly one-third of total mortality globally [5]. Together with three other AHT medicines (i.e., angiotensin-converting enzyme inhibitors (ACEI), angiotensin receptor blockers (ARB) and thiazide or thiazide-like diuretics), dihydropyridine CCBs have been recommended for initiation as first line therapy in many countries [6, 7].

It has been hypothesised that CCBs may increase the risk of cancer by changing intracellular calcium levels, thereby affecting the process of apoptosis (programmed cell death), disabling the destruction of mutated cells and leading to development of diseases such as cancer in which there is indiscriminate replication of a mutated cell [8]. Calcium is known to play a regulatory role in apoptosis via multiple biochemical / pharmacological pathways [9-11]. It has been postulated that, due to its secretory nature, breast tissue may be more vulnerable to alterations in apoptotic activity than other tissue, leading to complex hormone-related relationships between apoptosis and breast carcinoma [12]. In addition, one particular CCB, nifedipine, has been found to increase proliferation and migration of breast cancer cells in vitro [13] and to reduce resting calcium concentration and apoptotic gene expression in mice [14].

In 2017, we published a systematic review and meta-analysis [15] highlighting the uncertainty regarding the association between long-term use of CCBs and breast cancer, primarily due to the limited availability of long-term follow-up data. This review and meta-analysis found that the literature is equivocal on whether CCBs are a risk factor for breast cancer. Li et al. [16] reported that long-term

For peer review only

(>10 years) use of CCBs was associated with a 2.4-fold (95% confidence intervals [CI], 1.2-4.9) increased risk of invasive ductal breast cancer and 2.6-fold (95% CI, 1.3-5.3) increased risk of invasive lobular breast cancer. In contrast, Grimaldi-Bensouda et al.[17], using data from the United Kingdom, and Wilson et al. [18], using data from the United States (US), from sisters with and without breast cancer failed to find an association between CCB use and breast cancer (odds ratio 0.95 (95% CI: 0.87-1.04) and hazard ratio 0.88 (95% CI 0.58-1.33), respectively). A similar pattern was observed in a recent study conducted by Rotschild et al (2022) in Israel, revealing an OR 0.997 (95% CI: 0.962-1.034) for a long term exposure to CCBs (above 8 years) and breast cancer risk [19]. A non-significant association was found in US women aged 55+ years for CCB use but a protective effect for ACEI use [20]. However, a study by Gómez-Acebo et al. [21] using Spanish data found CCB use for five or more years was associated with a 1.77-times increased risk (95% CI, 0.99-3.17) of breast cancer. A 2021 systematic review of clinical trials showed no strong effect of any type of AHT medicine on breast cancer with a median follow-up of 4.2 years. However, a systematic review of cohort studies with longer follow-up (≥10 years) published in the same year, found the use of beta blockers, CCBs and diuretics were associated with increased breast cancer risk [22]. The authors noted the heterogeneity of study design, study duration and capture of drug exposure status and also that most studies were from Europe or North America, limiting external validity.

Recommendations for CCB use are different across countries. Calcium Channel Blockers are currently the second most commonly prescribed AHT medicine and are considered first-line treatment for hypertension in Australia [23]. In contrast, CCBs are not recommended for first-line therapy in the Netherlands, where thiazide diuretics are more commonly initially prescribed for blood pressure control [24] and this represents an opportunity to investigate the role of prescribing practices in cancer risk. Variations in prescribing patterns may drive the differences in the associations observed in studies of CCB use and risk of breast cancer across populations in addition to differences in underlying cancer risk due to varying environmental or biological/genetic factors (e.g., body mass index [BMI], physical activity, smoking and alcohol history, hormonal medicine use or mammographic density). We expect this to be true for CCB prescribing in Australia compared with The Netherlands. The similarities and differences between the Dutch and Australian cohorts have been leveraged previously in studies of

For peer review only

medicine safety [25]. We therefore propose to use data from these two countries to: (i) explore confounding and (ii) effect modification by these specific factors in the relationship between CCB use and breast cancer risk.

Long-term evaluation of medicine safety is best monitored via longitudinal pharmacovigilance and observational studies, as they have both linked claims data and good confounder adjustment obtained through surveys of participants whereas randomised clinical trials with sufficient follow-up are largely not feasible due to time and cost factors. Thus, we aim to examine whether long-term CCB use is associated with the development of breast cancer amongst women enrolled in three longitudinal cohort studies in Australia and The Netherlands and to characterise the dose-response nature of any such association. A secondary aim is to assess whether any differences in the association between CCB use and the development of breast cancer exist between Australian and Dutch women and, if so, to explore confounding and effect modification for a range of socio-economic and clinical factors.

Methods and analysis

Study design

This will be a retrospective, observational study conducted across three internationally renowned longitudinal cohorts incorporating both self-report and administrative data linked at the person-level. For the primary aim, the study will use a distributed methodological approach, which has been shown to assure methodological quality and transparency and increase the likelihood that differences between results are due to true variation in effect size, rather than design or process-related problems [26]. Our analysis will: (i) first use identical protocols across cohorts (harmonised analyses) and then (ii) use variable/cohort-specific protocols to allow the influence of cohort-specific variables to be investigated (non-harmonised analyses).

Data sources

We will utilise data from the Australian Longitudinal Study on Women's Health (ALSWH) [27], the New South Wales (NSW) Sax Institute's 45 and Up Study (45 and Up Study) [28] and the Rotterdam Study [29].

- The ALSWH [27] is a national study that began in 1996 with a random sample of more than 40,000 women in three birth cohorts. The surveys were conducted every three years since 1996,

For peer review only

however, the 1921–26 cohort was surveyed 6 monthly from Nov 2011 for health and lifestyle changes. In our study, we will use two longitudinal cohorts, the 1946–51 cohort (aged 45–50 years at recruitment) and the 1921–26 cohort (aged 70–75 years at recruitment).

- Administrative data for the ALSWH are included for all Australian states and territories, linked using probabilistic linkage with clerical review by the data linkage units in each state. Linked data used for this study include hospital admissions, emergency department presentations, cancer registry and the national death index data. Hospitalisation and cancer registry data are recorded separately in each Australian state; hence the start and end of these datasets vary according to the availability of the data in each state. Medicare Benefits Schedule (MBS) claims and Pharmaceutical Benefits Scheme (PBS) data were provided by Services Australia.
- The 45 and Up Study [28] is a longitudinal cohort of 267,357 participants aged ≥45 years residing in NSW, Australia at recruitment randomly sampled from the Services Australia Medicare enrolment database. Participants from rural and remote areas and those 80+ years of age were oversampled. Participants were recruited from 2005-2009 (2005 being a pilot study) and followed up every five years for changes in health and lifestyle. The response fraction was approximately 19%. Only female participants (53.6% of the cohort) recruited in the main study between 2006 and 2009 will be included in the proposed study.
- Survey data for the 45 and Up Study were linked to the NSW Admitted Patient Data Collection and the Australian Capital Territory (ACT) Admitted Patient Collection, the NSW and ACT Emergency Department Data Collection, the NSW and ACT Cancer Registry, the NSW and ACT Registry of Births Deaths & Marriages death registrations, the Australian Coordinating Registry Cause of Death Unit Record File, NSW and ACT BreastScreen and the NSW Pap Test Registry by the Centre for Health Record Linkage (CHeReL) using a probabilistic linkage procedure with clerical review. The current estimated rate of false-positive linkages is 0.5% (www.cherel.org.au). Linkage of 45 and Up Study cohort data to Medicare claims and PBS data provided by Services Australia was facilitated by the Sax Institute using a unique identifier and deterministic matching.
- The Rotterdam Study [29] is a prospective cohort study of persons aged ≥45 years living in the

For peer review only

city of Rotterdam, The Netherlands. The study started with a pilot phase in the second half of 1989, called “RS-I”. A second cohort was recruited in 2000 (RS-II) and a third cohort in 2006 (RS-III). By the end of 2008, the Rotterdam Study comprised 14,926 participants aged 45 years or over. At baseline, participants were interviewed and had a clinical examination that was repeated every 3 to 4 years. Follow-up of participants between clinical examinations is undertaken via automated coupling of the study database with medical records from general practitioners (GPs), who receive all relevant medical information from all caregivers of their patients. The data include hospital and specialist discharge summaries providing dates of diagnoses and major surgical interventions. Only female participants of the three longitudinal cohorts will be included in our study.

- The three Rotterdam longitudinal cohorts are linked at the individual level to records for dispensed medicines from all pharmacies in the study area (1991-2018), the Netherlands Comprehensive Cancer Organisation (1989-2016) and municipality (death) records (1989-2023).

A summary of the source of the major categories of data available for the study is provided in Table 1.

Study population

The same study population will be used for the primary and secondary aims. Entry to our study will be on survey completion date for women who participated in the ALSWH survey waves between 2004 and 2008 or the 45 and Up Study baseline survey between 2006 and 2009 or the date of the Rotterdam Study interview and clinical examinations for women who completed these between 2004 and 2008. Inclusion criteria will be women who at entry to our study (i) were alive and consented to linkage of administrative data, (ii) have evidence of hypertension from either self-reported hypertension (Australian longitudinal cohorts) or have a recorded diagnosis of hypertension in clinical examination data (Rotterdam cohorts). Women will be excluded at entry if they had (i) history of primary invasive/in situ breast cancer or cancer that is metastatic to the breast; or (ii) history of bilateral mastectomy; or (iii) evidence of CCB use prior to entry. Further exclusion criteria will be applied in Australian longitudinal cohorts only as per standard practice when using PBS data: (i) women with any evidence of being Department of Veterans’ Affairs (DVA) concession card holders, because DVA-subsidised prescriptions are funded separately and not

For peer review only - http://bmjopen.bmj.com/site/about/guidelines.xhtml

captured in the PBS data held; or (ii) women who did not have evidence of concessional healthcare status for more than one year during period from 2003 to 2012. Those with concessional status, e.g., those receiving the aged care pension or certain other Government benefits, have lower co-payments for PBS-listed medicines in comparison to “general beneficiaries”. This exclusion is necessary because some AHT medicines fall below the PBS co-payment threshold for general beneficiaries, hence attracted no government reimbursement and were not captured in PBS data prior to an administrative change in 2012 [30]. In contrast the lower co-payment threshold for concessional patients means that AHT medicines can be captured throughout the study period.

Participants will be followed until the earliest date of one of the following: the outcome (a diagnosis of invasive breast cancer); a competing risk event (evidence of bilateral mastectomy without a diagnosis of breast cancer, death prior to any diagnosis of breast cancer) or end of the study follow-up (censoring event), whichever came first. Note that since the duration of the available data differs across the longitudinal cohorts, end of study follow-up will be established for each person based on the data available. Figure 1 presents a schematic diagram of timeline for entry, exposure and study exit (i.e., recording of the outcome, censoring, competing event or end of follow-up) of our study population.

As administrative data are available prior to entry, this allows for a “lookback” period to ascertain some aspects of health service use prior to study entry, for comorbidity ascertainment and to remove those with a history of CCB use or breast cancer diagnosis prior to time-zero for follow-up.

Harmonisation of data

Harmonisation of data will be undertaken using the six-step process described by Rolland et al. [31] and Fortier et al. [32] After obtaining approvals from all data access and ethics committees, the Australian data will be housed in cohort-specific folders in the Sax Institute’s Secure Unified Research Environment (SURE) (https://www.saxinstitute.org.au/solutions/sure/), whereas the Rotterdam data will be uploaded and analysed in the Accessible Network Digital Research Environment Alliance (AnDREa) (www.andrea-cloud.eu). Related documents such as data processing scripts, and comments on specific decisions taken throughout the process will be recorded. An associated data dictionary will also be developed. To ensure content equivalence, each variable will be checked on (1) the definition used in the questionnaire, format, categories, unit, and time frame, (2) measurement method (e.g., self-

For peer review only - http://bmjopen.bmj.com/site/about/guidelines.xhtml

reported, clinical examination, etc.), and (3) harmonisation rules. Variables relating to the main exposure, outcomes/competing risk, socio-demographic, lifestyle, clinical, reproductive characteristics and healthcare utilisation that are harmonisable, together with those available for the cohort-specific analyses, are presented in Table 2. Where a variable has different categories across cohorts we will collapse categories to generate the closest possible match.

Exposure ascertainment

The exposure in this study, for both the primary and secondary aims, will be CCB dispensing including both combination and monotherapy products. Exploring the impact of confounding by indication for non-CCB AHT medicines is important because a modest increased risk of cancer in individuals with hypertension has been documented [33]. To avoid this issue, previous studies have used a comparator group of patients exposed to other AHTs and/or with a hypertension diagnosis [34]. Our study population is defined by the presence of hypertension since comparing only to a reference AHT medicine group risks biasing toward the null, if the comparator drug is associated with the outcome. Thus, to reduce bias to the null, women who are both AHT and CCB naïve will be included in the study as long as they meet the entry criteria of hypertension at baseline as described previously. To ensure that exposure to other AHT medicines is accounted for the cumulative dose-duration exposure of other prescribed AHT medicines (combined) will be captured in the same way as that described for CCB exposure. Thus in our study the following four exposure states will be possible during follow-up: (i) women with no exposure to either CCBs or other AHTs; (ii) women exposed to other AHTs but not exposed to CCBs; (iii) women exposed to CCBs but not exposed to other AHTs and (iv) women exposed (at any time) to both CCBs and AHTs. To compensate for immortal-time bias, and to be able to capture the dose-response nature of the association of CCB exposure with risk of breast cancer, a time-varying continuous ascertainment of cumulative exposure (dose-duration) to CCB (and separately other AHTs) will be captured as follows [35].

We will use the Anatomical Therapeutic Chemical (ATC) codes [36] and information on dispensed medicines in the PBS data for Australian longitudinal cohorts and medicine record data for the Rotterdam longitudinal cohorts to identify AHT drugs dispensed for the study population from study entry to exit. We will categorise AHT drugs dispensed into five main AHT drug classes: (i) beta-blocking agents (BB)

For peer review only - http://bmjopen.bmj.com/site/about/guidelines.xhtml

(ATC code C07), (ii) CCB (C08); (iii) diuretics (C03), (iv) agents acting on the renin-angiotensin system (ACEIs and ARBs) (C09), or (v) other AHTs not included in these classes regardless of treatment therapies (i.e., monotherapy or combination therapy). Details of level 5 ATC codes of the dispensing medicines are presented in Appendix Table 1.

For all longitudinal cohorts, the total cumulative duration exposed and the total cumulative dose of each AHT drug class will be calculated from the medicine records available from entry to exit of the study. For all cohorts the data includes information on the date of dispensing, quantity dispensed and the strength of the medicine (derived from the PBS item code for Australian data). In the Rotterdam data, the daily prescribed dose and prescribed duration of use are also available [29]. This information is not present in the Australian data necessitating estimation of the duration of the medicine. For the Australian data, the reverse waiting time distribution method with multiple random index dates will be used to determine the following: i) the median duration of medicine possession, defined as the number of days that 50% of prevalent users of the same medicine refill their scripts and ii) the upper duration limit of medicine possession, defined as the number of days that 80% of prevalent users of the same medicine refill their scripts [37, 38]. The median duration will be used as the usual possession duration of the medicine in calculating the total duration (days) of medicine possession in contiguous periods of exposure. The contiguous period will be defined as a series of dispensings of the same AHT class with no break between dispensing that is greater than the upper duration limit of medicine possession of the last medicine in the series. The total cumulative duration (days) of exposure to each AHT class under investigation for each individual over the study period will be the sum of the number of days accrued over all contiguous periods of exposure. The total cumulative dose will be similarly captured across all contiguous periods of exposure by summing the total milligrams (mg) of medicine dispensed of all dispensations (strength/unit multiplied by total units dispensed).

Contiguous periods of exposure will be accounted for any carry-over of over-supply. The carry-over will only be applied if the next dispensing is the same medicine (same ATC code at the fifth level) as suggested previously [39]. The start of contiguous periods will be deemed the date of first-time dispensing observed within a series. End of a contiguous period will be the last dispensing date in the series plus the usual duration of medicine possession and any eligible carry-over or the date of exit of

For peer review only - http://bmjopen.bmj.com/site/about/guidelines.xhtml

the study where the duration of medicine possession continues past this date. Details of cumulative dose and duration are presented in Figure 2.

In addition, time since last CCB exposure will be calculated in months from exit of the cohort back to the date of the last CCB exposure to differentiate between recent and previous exposure. This value will range from 0 for those with current exposure at the time of exit to the total of the follow-up time for those who are never exposed.

In the statistical model dose-duration will be captured by three elements, total cumulative duration, total cumulative dose and an interaction term to evaluate effect modification between duration and dose.

Outcome ascertainment

During follow-up, incident diagnoses of invasive breast cancer will be identified using the International Classification of Diseases (ICD) version 10 Australian Modification [40] for the Australian cohorts and the ICD-10 [41] for the Rotterdam Study. ICD-10 code C50.x (malignant neoplasm of breast) will be used to identify invasive breast cancer diagnosis across cancer registries and hospital admission data. In situ breast cancer (e.g. D05.x) will not be included in the outcome because hyperplasia and atypical lesions are not precancerous (although some are risk factors), and only some ductal carcinoma in situ are obligate precursors [42, 43].

Cancer registries for all cohorts contain, by statutory requirement, records of all cancers diagnosed or treated and are therefore considered internationally the 'gold standard' for cancer identification for research purposes. Since cancer diagnoses for Dutch women and cancer registry data for the Australian women are only currently available until 2016 and 2018 respectively, breast cancers diagnosed later than these dates will be identified from general practitioner data (Dutch women) and hospital data (Australian women). Hospital-derived diagnoses for breast cancer have a positive predictive value and sensitivity of 86% and specificity of 99.9% compared with the cancer registry in the 45 and Up Study population [44]. Sensitivity analyses showed that 53% of women with a cancer registry diagnosis had an inpatient diagnosis within the same calendar month and 93% within one calendar month, thus estimations of date of diagnosis align well with Cancer Registry dates.

The date of breast cancer diagnosis used in the study will be the date of diagnosis recorded in either the Australian Cancer Registry or the Rotterdam Study, or the date of the first hospital admission with a

For peer review only

For peer review only - http://bmjopen.bmj.com/site/about/guidelines.xhtml

principal diagnosis of breast cancer / date recorded in the GP data where registry/study data are unavailable.

Covariates

A wide range of socio-demographic, lifestyle and clinical potential confounders that may impact prescribing decisions for AHT medicines, a participant's decision to take prescribed AHT medicines or be associated with an increased risk of breast cancer will be included in the analysis [45]. Variables will include the following captured at baseline: age, marital status, highest qualification, socioeconomic disadvantage, remoteness, BMI, diabetes, heart disease, stroke, diagnosis of other cancer, comorbidity risk score, number of children and history of hysterectomy/both ovaries removed. In addition, the following time varying factors (updated annually throughout follow-up and at exit) will be included: statin use, use of combined hormonal contraception, use of combined hormonal replacement therapy. Confounding by comorbidity will be assessed at baseline and also as a time varying covariate as per the Rx-Risk score (with 5 years look back) that includes 46 clinical conditions based on ATC codes from prescribed medicine claims data, excluding AHT drugs as these medicines are captured in the exposure [46]. For the secondary aim (cohort-specific analyses) additional covariates will be available depending upon the cohort; for example for the 45 and Up Study cohort mammographic screening information will be used to differentiate screen-detected cancers, in the ALSWH and Rotterdam cohorts multiple waves of self-reported/clinical examination data will be used to update lifestyle information during follow-up. In addition, where possible we will explore the use of earlier study entry to provide longer follow up. For both Australian cohorts hospital admissions, all specialist services, and GPs visits will be used as time varying covariates to account for changing propensity to interact with health services. Differences between the cohort specific analysis and the harmonised analysis undertaken for each cohort will be evaluated to aid with determination of the influence of cohort specific variables on the association.

Details of the relevant codes (MBS item numbers (Australian women), ATC codes and ICD codes) used to identify participants' medical conditions, prescribed medicines and health service use from MBS, PBS/pharmacy data, hospital admission, GP data, cancer registries and mortality data collections are

For peer review only

provided in Appendix Table 2. A description of data items, questions and response categories from the baseline surveys of the ALSWH and the 45 and Up Study are presented in Appendix Table 3.

Statistical analysis

Primary aim: For the primary aim, analysis of the association between CCB use and the development of breast cancer, will be undertaken using Fine and Gray competing risks regression with incident breast cancer as the principal event, and death (from causes other than breast cancer) or bilateral mastectomy as the competing risk [47]. In this setting, the appropriate estimate of the probability of breast cancer is described by the cumulative incidence function rather than a hazard ratio. The models will account for confounding factors at entry using propensity scores to balance these factors across exposure status captured over the follow-up period. In addition, we will use standard covariate adjustment for time varying covariates and any covariates that cannot be balanced using the propensity score. Any significant shifts in practice will be identified via the literature (and our clinical collaborators) and included as categorical variables in the models where appropriate.

Since the CCB exposure variable will be continuous rather than binary a generalisation of the binary propensity score, the generalised propensity score (GPS) developed by Hirano and Imbens, will be used [48]. Since CCB exposure will be sporadic, we will also evaluate the dose-duration response effect by further extending the multivariable models to incorporate a time-dependent dose-response component [49]. Specifically, the dose-duration response function will be estimated after adjusting for covariate imbalance by using the GPS methods applied in previous publications [49-52]. The non-linear predictive relationships between the dose-duration of CCB exposure and breast cancer will be modelled using nonlinear threshold models developed by Gannon & Harris [53]. Threshold models allow for the differential effect of the dose-duration of CCB exposure on the incidence of breast cancer with respect to an individual's duration and cumulative exposure to CCBs. The model will allow the coefficient (β) to vary according to the days/months/years per unit of the cumulative dose-duration while assuming that other components in the model are constant across all individuals to identify the thresholds for subpopulations [54]. The estimation of the threshold models involves searching all possible values of the dose-duration function with maximum likelihood estimators computed for every tried value. For practical purposes, the number of subpopulations will be limited to three or less [54]. The

For peer review only - http://bmjopen.bmj.com/site/about/guidelines.xhtml

threshold model that minimises the Bayesian information criterion used for selection of the non-linear models will be the final model. The counterfactual, individuals with no CCB exposure, will also be analysed as a baseline to show the impact of no versus any treatment using standard binary propensity score-matching of those exposed versus non-exposed [51].

Secondary aim: For the secondary aim we will explore relationships between dose-duration of CCB use and incident breast cancer and factors that confound and modify these relationships. Using multilevel modelling techniques and time-varying covariates, we will quantify the contributions of person-, temporal- and geo-spatial level-related factors to variation in the dose-duration effect. This will allow the development of both an inter- and intra-country-specific profiles of women who are at higher and lower risk of CCB-attributable breast cancer.

Analyses will be adjusted for multiple comparisons where appropriate. Where the sample size is insufficient to confirm the nature of the association, the results will be regarded as exploratory. Analyses will use Stata SE version 15 [55]. The analysis is scheduled to commence in February 2024 and conclude in July 2025.

Subgroup analysis

In addition to our analysis of overall CCB exposure, the analysis for each aim will be conducted separately according to CCB class (i.e., dihydropyridines and non-dihydropyridines) and duration of action (i.e., short or long action) where sufficient statistical power exists. Moreover, we will conduct a subgroup analysis to assess the influence of a personal history of any cancer at cohort entry on the risk of breast cancer and CCB exposure.

Sensitivity analyses

We will conduct several sensitivity analyses. Since hyperplasia and atypical lesions are not precancerous, we will test the effect of adding ductal carcinoma in situ as a competing risk in sensitivity analyses rather than as an exclusion criterion. We will explore the impact of imposing various time lags, based on clinical consultation, between the start of follow-up on the outcome to account for latency and plausible timing between exposure and outcome development. The potential impact of reduced exposure resulting from imperfect adherence and prescribing at defined daily dose levels (a statistical measure of drug consumption, defined by the World Health Organization) will be investigated in the

For peer review only - http://bmjopen.bmj.com/site/about/guidelines.xhtml

dose-duration models and through sensitivity analyses [36]. We will expand the study population to women with prior CCB use at entry (captured as a starting dose-duration value using available look-back data) to examine the impact of CCB use (overall) on the outcome. Finally, because concessional status for subsidised medicines in Australia can change with age or employment status, we will explore varying the definition of continuous PBS concessional status in Australian women to determine the impact this has on the association of CCB exposure with the outcome. The main analysis for the study will be evidence of 100% concessional status between 2003 and 2012 with a single year of no evidence allowed (i.e., at least one dispensation per year, and patient recorded as being on concessional status on each dispensation, with one year allowed to vary). This will be varied allowing two years of no evidence (the more relaxed scenario) and requiring 100% concessional status across all years (as the most stringent requirement). Sensitivity analyses will be applied to the primary aim in the first instance, and those factors found to be influential will be additionally tested in the secondary aim. Due to the potential duplication of participants who were part of the ALSWH and also enrolled in the 45 and Up Study residing in NSW, we will systematically exclude these ALSWH participants in a sensitivity analysis. This exclusion is essential for evaluating the potential impact of duplication on the study results.

Sample size calculation

Breast cancer is a relatively common event for the studies under investigation, thus our sample size will be based on the women exposed to CCBs (as indicated by the numbers on the right-hand side of Figure 3). Using a conservative ratio of unexposed: exposed of 5:1, we have 95% confidence with 80% power to detect a minimum effect size of +/- 0.2 for breast cancer associated with CCB use in all datasets given the number of exposed individuals.

Individual patient data meta-analysis

For the primary aim a two-step (since the data cannot be brought together) random effects individual patient-level meta-analysis which allows for assessment of effect modification (i.e. interaction terms) by country or personal characteristics will be undertaken [56]. First, each individual study estimate will be calculated, plotted using a forest plot and compared to observe any similarities and differences. Next, individual estimates will be weighted using the Knapp-Hartung method and pooled using random effects methods taking into account both within and between variance as below [57]:

For peer review only

$$\beta'_s = \beta + U_s + \varepsilon_s, \; s = 1 \text{ to s studies}$$

where β = mean true effect estimate, ε_s = within study variance, U_s = between study variance and β'_s = estimated effect estimate in study s.

Patient and public involvement

There was no patient or public involvement in the design of the proposed study.

Ethics and dissemination

Ethical approval was obtained from the following Human research Ethics Committees: Curtin University (HRECs) (ref No. HRE2022-0335), NSW Population and Health Services Research Ethics Committee (2022/ETH01392 / 2022.31), ACT Research Ethics and Governance Office approval under National Mutual Acceptance for multi-jurisdictional data linkage research (2022.STE.00208). For the ALSWH survey collections has ongoing ethical approval from the HREC of the Universities of Newcastle and Queensland (approval numbers H076-0795 and 2004000224, respectively). The ALSWH also maintains institutional HREC approvals for external record linkage (approval numbers H-2011-0371 and 2012000132, respectively). In addition, access to national collections include MBS/PBS, National Death Index, Australian Cancer Database is approved by the Australian Institute of Health and Welfare. Access to state and territory data collections include hospital admissions and cancer registries is approved by an appropriate HREC for each jurisdiction [58]. These data are approved by individual ethics committees and custodians in different jurisdictions, though the researchers make a single application to the ALSWH Data Access Committee, who then contact individual custodians on behalf of researchers. The Rotterdam Study data has been approved by the Medical Ethics Committee of the Erasmus MC (registration number MEC 02.1015) and by the Dutch Ministry of Health, Welfare and Sport (Population Screening Act WBO, license number 1071272-159521-PG). The Rotterdam Study Personal Registration Data collection is filed with the Erasmus MC Data Protection Officer under registration number EMC1712001.

Each of the studies collected informed consent from all participants at recruitment. Women who participated in earlier waves of the ALSWH provided consent for access to administrative health data including the MBS, PBS, national death index, cancer registries, and hospital records for most Australian States and Territories. In later waves, there has been a change to opt-out consent of these

For peer review only - http://bmjopen.bmj.com/site/about/guidelines.xhtml

linkages, with participants provided information on the datasets that may be linked and a freecall phone number to opt out provided to participants at recruitment and in an annual newsletter. All study participants in the 45 and Up study consented to their survey data being linked to routinely collected health administrative data and health registry sources. All participants in the Rotterdam Study provided written informed consent to participate in the study and to have their information obtained from treating physicians.

Results of the proposed study will be published in high-impact journals and presented to key stakeholders via scientific meetings, lay press (print media, television, radio and internet).

Discussion

The equivocal literature regarding the potential association between CCB exposure and breast cancer is likely due to differences in study design (e.g., cohort vs case-control), length of follow-up, exposure measurement (e.g., ever vs never or time duration) or population characteristics (e.g. patients with hypertension and/or cardiovascular disease vs general population cohorts, which have differing risk of confounding by indication). These differences, along with small sample sizes in many studies (often n<1,000), make inference difficult. Randomised, controlled trials are not usually suitable for identifying long-term adverse effects of medicine use and long-term monitoring through observational studies using administrative datasets has become the standard way of overcoming this limitation for post-market surveillance research [59]. However, the sole use of administrative data limits the ability to control for critical lifestyle and other potential confounding factors that are not routinely reported in such datasets, e.g., BMI, physical activity, smoking and alcohol use history. In many studies, longitudinal cohorts have been used to alleviate this issue but often these studies have suffered from reporting and/or recall bias due to the reliance on self-report data, especially regarding the determination of exposure relating to prescription medication use. This has led to the preponderance of crude ever/never exposed to CCBs in the literature rather than more complex dose-duration measures that we propose.

Our study will involve six longitudinal cohorts of women extracted from internationally renowned Australian and Dutch studies linked with administrative health data, with complete coverage, to allow for continuous capture of CCB exposure for an extended period of time (i.e. up to 19 years). Using

For peer review only

state-of-the-art analytical techniques such as dose-duration methods previously unused in this area, this study will evaluate the association and characterise the dose-response nature of any such association between long-term CCB use and the risk of breast cancer. A wide range of person-, temporal- and geo-spatial level-related factors will be measured and adjusted for to separate the effect of these factors from the relationship between CCB use and breast cancer. The analytic methods (non-linear threshold modelling) proposed are novel and will be a significant innovation to pharmaco-epidemiological analyses, which currently lack such sophisticated methods.

Due to the nature of the observational study design, confounding by indication will be unavoidable. To minimise the impact of this issue, the study will be limited to participants with a diagnosis/self-reported hypertension and comparisons will include participants exposed to other prescribed (non-CCB) AHT medicines in addition to those who have no pharmacotherapy for hypertension. As explained in the methods, the Australian cohorts will be restricted by concessional status to capture the AHT exposure over the study duration, and our study is therefore likely prone to selection bias; this will be investigated in the sensitivity analyses. Complete ascertainment of prior exposure will not be possible since we will not have lifetime capture of prescribing data, therefore CCB exposure may be under-reported. Due to the nature of medicine dispensing data, the level of certainty regarding whether the dispensed medicine (e.g. combined hormonal contraceptives, combined menopausal hormone therapy and CCB / AHT medicines) was consumed by the patient is unclear. The diagnostic interval of breast cancer may vary between Australian and Dutch women, particularly since case ascertainment through cancer registries can take months from the initial diagnosis to coding notifications. Previous studies have shown a difference around 30 days in the diagnostic interval days depending on the presentation of symptoms and the temporal differences in diagnostic pathways between the two countries [60]. Another limitation is the absence of genetic data for this analysis. However, recent studies have indicated no association between genetic proxies for CCB or other antihypertensives and breast cancer through Mendelian randomisation study design [61, 62].

While we have used the term "association" here, our use of GPS adjustment provides a mechanism for undertaking analysis under a causal framework. Modelling each woman's propensity to receive the

For peer review only

exposure as a function of covariates means the GPS is independent of the outcomes and the study represents a quasi-experiment. We prefer to use the terminology "association" when reporting our results so as not to overstate our ability to adjust for potential confounding. Interpretation of any association will incorporate the likelihood of causation (using the Bradford-Hill criteria) [63].

With regards to representativeness and generalisability of our results, leading epidemiologists [64] and reputable peer-reviewed publications [65, 66] support the principle that cohort studies rely on the validity of internal comparisons to avoid systematic error and need not be a random sample of a larger population to provide generalisable knowledge. The validity of generalisation (i.e., external not internal validity) depends on considerations about risk stratification and effect modification rather than bias/confounding. Thus, results of well-respected international cohort studies, such as the British Doctors' Study and US Nurses' Health Study, are regarded as providing generalisable knowledge despite their highly selected study samples.

The literature continues to debate whether or not CCBs increase the risk of breast cancer. The outcomes of the proposed study are likely to have considerable implications for both clinical practice and public health. If a positive association is found, this could have a global impact on clinical guidelines for treating hypertension in women. Long-term CCB use has the potential to become the number one major modifiable risk factor for breast cancer. Conversely if no association is found, this would provide reassurance to Australian and Dutch women that the current guidelines promoting CCBs as a first line therapy for hypertension are appropriate and that taking a CCB does not pose additional breast cancer risk.

For peer review only

References

1. Sung H, Ferlay J, Siegel RL, Laversanne M, Soerjomataram I, Jemal A, et al. Global cancer statistics 2020: GLOBOCAN estimates of incidence and mortality worldwide for 36 cancers in 185 countries. CA: a cancer journal for clinicians. 2021;71(3):209-49.

2. Arnold M, Morgan E, Rumgay H, Mafra A, Singh D, Laversanne M, et al. Current and future burden of breast cancer: Global statistics for 2020 and 2040. The Breast. 2022;66:15-23.

3. Fuchs FD, Whelton PK. High blood pressure and cardiovascular disease. Hypertension. 2020;75(2):285-92.

4. Lopez A, Mathers C, Ezzati M, Jamison D, Murray C. Global and regional burden of disease and risk factors, 2001: systematic analysis of population health data. Lancet. 2006;367:1747-57.

5. World Health Organisation. A global brief on hypertension: Silent killer, global public health crisis. Document number : WHO/DCO/WHD/2013.2 Geneva: WHO; 2013.

6. Visseren FL, Mach F, Smulders YM, Carballo D, Koskinas KC, Bäck M, et al. 2021 ESC Guidelines on cardiovascular disease prevention in clinical practice: Developed by the Task Force for cardiovascular disease prevention in clinical practice with representatives of the European Society of Cardiology and 12 medical societies With the special contribution of the European Association of Preventive Cardiology (EAPC). European heart journal. 2021;42(34):3227-337.

7. National Vascular Disease Prevention Alliance. Guidelines for the management of absolute cardiovascular disease risk. 2012.

8. Daling JR. Calcium channel blockers and cancer: is an association biologically plausible? American journal of hypertension. 1996;9(7):713-4.

9. Wyllie AH. Glucocorticoid-induced thymocyte apoptosis is associated with endogenous endonuclease activation. Nature. 1980;284(5756):555-6.

10. Szalai G, Krishnamurthy R, Hajnóczky G. Apoptosis driven by IP3-linked mitochondrial calcium signals. The EMBO journal. 1999;18(22):6349-61.

11. Suzuki J, Umeda M, Sims PJ, Nagata S. Calcium-dependent phospholipid scrambling by TMEM16F. Nature. 2010;468(7325):834-8.

12. Parton M, Dowsett M, Smith I. Studies of apoptosis in breast cancer. Bmj. 2001;322(7301):1528-32.

13. Guo D-Q, Zhang H, Tan S-J, Gu Y-C. Nifedipine promotes the proliferation and migration of breast cancer cells. PloS one. 2014;9(12):e113649.

14. Altamirano F, Valladares D, Henríquez-Olguín C, Casas M, López JR, Allen PD, et al. Nifedipine treatment reduces resting calcium concentration, oxidative and apoptotic gene expression, and improves muscle function in dystrophic mdx mice. PloS one. 2013;8(12):e81222.

For peer review only

For peer review only - http://bmjopen.bmj.com/site/about/guidelines.xhtml

15. Wright CM, Moorin RE, Chowdhury EK, Stricker BH, Reid CM, Saunders CM, et al. Calcium channel blockers and breast cancer incidence: An updated systematic review and meta-analysis of the evidence. Cancer epidemiology. 2017;50:113-24.

16. Li CI, Daling JR, Tang M-TC, Haugen KL, Porter PL, Malone KE. Use of antihypertensive medications and breast cancer risk among women aged 55 to 74 years. JAMA internal medicine. 2013;173(17):1629-37.

17. Grimaldi-Bensouda L, Klungel O, Kurz X, De Groot MC, Afonso ASM, De Bruin ML, et al. Calcium channel blockers and cancer: a risk analysis using the UK Clinical Practice Research Datalink (CPRD). BMJ open. 2016;6(1):e009147.

18. Wilson LE, D'Aloisio AA, Sandler DP, Taylor JA. Long-term use of calcium channel blocking drugs and breast cancer risk in a prospective cohort of US and Puerto Rican women. Breast Cancer Research. 2016;18(1):1-8.

19. Rotshild V, Hirsh Raccah B, Gazawe M, Matok I. Calcium Channel Blocker Use and the Risk for Breast Cancer: A Population-Based Nested Case-Control Study. Cancers. 2022;14(9):2344.

20. Raebel MA, Zeng C, Cheetham TC, Smith DH, Feigelson HS, Carroll NM, et al. Risk of breast cancer with long-term use of calcium channel blockers or angiotensin-converting enzyme inhibitors among older women. American Journal of Epidemiology. 2017;185(4):264-73.

21. Gómez-Acebo I, Dierssen-Sotos T, Palazuelos C, Perez-Gomez B, Lope V, Tusquets I, et al. The use of antihypertensive medication and the risk of breast cancer in a case-control study in a Spanish population: the MCC-Spain study. PLoS One. 2016;11(8):e0159672.

22. Xie Y, Wang M, Xu P, Deng Y, Zheng Y, Yang S, et al. Association between antihypertensive medication use and breast cancer: a systematic review and meta-analysis. Frontiers in pharmacology. 2021;12:609901.

23. National Heart Foundation of Australia. Guideline for the diagnosis and management of hypertension in adults – 2016. Melbourne: National Heart Foundation of Australia. 2016.

24. Smulders Y, Burgers J, Scheltens T, Van Hout B, Wiersma T, Simoons M. Clinical practice guideline for cardiovascular risk management in the Netherlands. 2008.

25. Wagemaakers FN, Hollingworth SA, Kreijkamp-Kaspers S, Tee EH, Leendertse AJ, van Driel ML. Opioid analgesic use in Australia and the Netherlands: a cross-country comparison. International Journal of Clinical Pharmacy. 2017;39:874-80.

26. Platt RW, Platt R, Brown JS, Henry DA, H. KO, Suissa S. How pharmacoepidemiology networks can manage distributed analyses to improve replicability and transparency and minimize bias. Pharmacoepidemiol Drug Safety. 2018;29(S1):3-7.

27. Dobson AJ, Hockey R, Brown WJ, Byles JE, Loxton DJ, McLaughlin D, et al. Cohort profile update: Australian longitudinal study on women's health. Int J Epidemiol. 2015;44(5):1547-f.

28. Bleicher K, Summerhayes R, Baynes S, Swarbrick M, Navin Cristina T, Luc H, et al. Cohort Profile Update: The 45 and Up Study. Int J Epidemiol. 2022.

For peer review only

For peer review only - http://bmjopen.bmj.com/site/about/guidelines.xhtml

29. Ikram MA, Brusselle G, Ghanbari M, Goedegebure A, Ikram MK, Kavousi M, et al. Objectives, design and main findings until 2020 from the Rotterdam Study. European journal of epidemiology. 2020;35:483-517.
30. Paige E, Kemp-Casey A, Korda R, Banks E. Using Australian Pharmaceutical Benefits Scheme data for pharmacoepidemiological research: challenges and approaches. 2015.
31. Rolland B, Reid S, Stelling D, Warnik G, Thornquist M, Feng Z, et al. Toward Rigorous Data Harmonization in Cancer Epidemiology Research: One Approach. American Journal of Epidemiology. 2015;182(12):1033-8.
32. Fortier I, Raina P, Van den Heuvel ER, Griffith LE, Craig C, Saliba M, et al. Maelstrom Research guidelines for rigorous retrospective data harmonization. Int J Epidemiol. 2017;46(1):103-5.
33. Assimes TL, Elstein E, Langleben A, Suissa S. Long-term use of antihypertensive drugs and risk of cancer. Pharmacoepidemiology and drug safety. 2008;17(11):1039-49.
34. Sendor R, Stürmer T. Core concepts in pharmacoepidemiology: confounding by indication and the role of active comparators. Pharmacoepidemiology and drug safety. 2022;31(3):261-9.
35. Zhou Z, Rahme E, Abrahamowicz M, Pilote L. Survival bias associated with time-to-treatment initiation in drug effectiveness evaluation: a comparison of methods. American journal of epidemiology. 2005;162(10):1016-23.
36. WHO Collaborating Centre for Drug Statistics Methodology. ATC classification index with DDDs Oslo, Norway 2023 [Available from: https://www.whocc.no/atc_ddd_index_and_guidelines/atc_ddd_index/.
37. Pottegård A, Hallas J. Assigning exposure duration to single prescriptions by use of the waiting time distribution. Pharmacoepidemiology and drug safety. 2013;22(8):803-9.
38. Bødkergaard K, Selmer RM, Hallas J, Kjerpeseth LJ, Skovlund E, Støvring H. Using multiple random index dates with the reverse waiting time distribution improves precision of estimated prescription durations. Pharmacoepidemiology and Drug Safety. 2021;30(12):1727-34.
39. Arnet I, Greenland M, Knuiman MW, Rankin JM, Hung J, Nedkoff L, et al. Operationalization and validation of a novel method to calculate adherence to polypharmacy with refill data from the Australian pharmaceutical benefits scheme (PBS) database. Clinical epidemiology. 2018:1181-94.
40. Chronicle of The International Statistical Classification of Diseases and Related Health Problems, Tenth Revision, Australian Modification (ICD-10-AM) First Edition to Eleventh Edition [Internet]. 2019 [cited 22/08/2023]. Available from: https://www.ihacpa.gov.au/sites/default/files/2022-08/icd-10-am_chronicle_-_eleventh_edition.pdf.
41. Organization WH. International Statistical Classification of Diseases and related health problems: Alphabetical index: World Health Organization; 2004.
42. To T, Wall C, Baines CJ, Miller AB. Is carcinoma in situ a precursor lesion of invasive breast cancer? International journal of cancer. 2014;135(7):1646-52.

For peer review only

For peer review only - http://bmjopen.bmj.com/site/about/guidelines.xhtml

43. Dettogni RS, Stur E, Laus AC, da Costa Vieira RA, Marques MMC, Santana IVV, et al. Potential biomarkers of ductal carcinoma in situ progression. BMC cancer. 2020;20(1):1-9.

44. Kemp A, Preen DB, Saunders C, Holman CDAJ, Bulsara M, Rogers K, et al. Ascertaining invasive breast cancer cases; the validity of administrative and self-reported data sources in Australia. BMC medical research methodology. 2013;13(1):1-8.

45. Cancer Australia. Risk factors for breast cancer: A review of the evidence, Cancer Australia, Surry Hills, NSW. 2018.

46. Pratt NL, Kerr M, Barratt JD, Kemp-Casey A, Ellett LMK, Ramsay E, et al. The validity of the Rx-Risk comorbidity index using medicines mapped to the anatomical therapeutic chemical (ATC) classification system. BMJ open. 2018;8(4):e021122.

47. Fine J, Gray R. A proportional hazards model for the subdistribution of a competing risk. Journal of the American Statistical Association. 1999;94:496-509.

48. Hirano K, Imbens GW. The propensity score with continuous treatments. Applied Bayesian modeling and causal inference from incomplete-data perspectives. 2004;226164:73-84.

49. Hirano K, Imbens G. The propensity score with continuous treatments. In: Gelman A, Meng X, editors. Applied Bayesian Modeling and Causal Inference from Incomplete-Data Perspectives. Sussex: Wiley Interscience 2004. p. 73-84.

50. Guardabascio B, Ventura M. Estimating the dose-response function through a generalized linear model approach. STATA Journal. 2014;14(1):141-58.

51. Kluve J, Schneider H, Uhlendorff A, Zhao ZW. Evaluating continuous training programmes by using the generalized propensity score. J Royal Stat Soc. 2007;172(2):587-617.

52. Flores C, Flores-Lagunes A, Gonzalez A, Neumann T. Estimating the Effects of Length of Exposure to Instruction in a Training Program: The Case of Job Corps. Review of Economics and Statistics. 2011;94(1):153-71.

53. Gannon B, Harris D, Harris M. Threshold effects in nonlinear models with an application to the social capital-retirement-health relationship. Health Economics. 2014;23(9):1072-83.

54. Bessaoud F, Daures J, Molinari N. Free knot splines for logistic models and threshold selection. Computer Methods and Programs in Biomedicine. 2005;77(1):1-9.

55. StataCorp. Stata Statistical Software: Release 15. College Station, TX: StataCorp LLC; 2017.

56. Thomas D, Radji S, Benedetti A. Systematic review of methods for individual patient data meta- analysis with binary outcomes. BMC Medical Research methodology. 2014;14(79):1471-2288.

57. Bender R, Friede T, Koch A, Kuss O, Schlattmann P, Schwarzer G, et al. Methods for evidence synthesis in the case of very few studies. Research Synthesis Methods. 2018;9(3):382-92.

58. Australian Longitudinal Study on Women's Health. Current HREC approvals for ALSWH data linkage [4/07/2023]. Available from: https://alswh.org.au/alswh-hrec-approvals/.

59. Avorn J. In defense of pharmacoepidemiology—embracing the yin and yang of drug research. New England Journal of Medicine. 2007;357(22):2219-21.

For peer review only - http://bmjopen.bmj.com/site/about/guidelines.xhtml

60. Vedsted P, Weller D, Falborg AZ, Jensen H, Kalsi J, Brewster D, et al. Diagnostic pathways for breast cancer in 10 International Cancer Benchmarking Partnership (ICBP) jurisdictions: an international comparative cohort study based on questionnaire and registry data. BMJ open. 2022;12(12):e059669.

61. Fan B, Schooling CM, Zhao JV. Genetic proxies for calcium channel blockers and cancer: a Mendelian randomization study. Journal of Human Hypertension. 2023:1-5.

62. Yarmolinsky J, Díez-Obrero V, Richardson TG, Pigeyre M, Sjaarda J, Paré G, et al. Genetically proxied therapeutic inhibition of antihypertensive drug targets and risk of common cancers: A mendelian randomization analysis. PLOS Medicine. 2022;19(2):e1003897.

63. Hill AB. The environment and disease: association or causation? : Sage Publications; 1965.

64. Rothman KJ, Greenland S, Lash TL. Modern epidemiology: Wolters Kluwer Health/Lippincott Williams & Wilkins Philadelphia; 2008.

65. Willett WC, Blot WJ, Colditz GA, Folsom AR, Henderson BE, Stampfer MJ. Merging and emerging cohorts: not worth the wait. Nature. 2007;445(7125):257-8.

66. Mealing NM, Banks E, Jorm LR, Steel DG, Clements MS, Rogers KD. Investigation of relative risk estimates from studies of the same population with contrasting response rates and designs. BMC medical research methodology. 2010;10:1-12.

For peer review only

For peer review only - http://bmjopen.bmj.com/site/about/guidelines.xhtml

699 **Authors' contributions:** CLBH assisted in the study design and drafted the manuscript. NTH and DY
700 contributed to drafting sections relating to study design and methodology. RM, SAP, DBP, BHS, CMS,
701 WPA, CMR, JH, MKB, DY conceptualised, designed the study, obtained the funding, reviewed, and
702 revised the manuscript. CLBH, NTH, DY, WPA, MKB, JH, GDM, SAP, DBP, CMR, RR, CMS, BHS,
703 FJAvR, CW and RM revised the manuscript for important intellectual content. The author(s) read and
704 approved the final manuscript.

705 **Funding statement:** This work was supported by The National Health and Medical Research Council,
706 project grant APP2014896. The study funders had no role in the study design, conduct, manuscript
707 writing or decision to submit for publication.

708 **Competing interests:** No conflict of interest

709 **Acknowledgements:**

710 The research on which this manuscript is based was conducted as part of the Australian Longitudinal
711 Study on Women's Health by the University of Queensland and the University of Newcastle. We are
712 grateful to the Australian Government Department of Health and Aged Care for funding and to the
713 women who provided the survey data.

714 This research was also completed using data collected through the 45 and Up Study
715 (www.saxinstitute.org.au). The 45 and Up Study is managed by the Sax Institute in collaboration with
716 major partner Cancer Council NSW, and partners the Heart Foundation and the NSW Ministry of
717 Health. We thank the many thousands of people participating in the 45 and Up Study. The 45 and Up
718 Study baseline data were linked by the Centre for Health Record Linkage (CHeReL)
719 www.cherel.org.au.

720 The authors acknowledge the Rotterdam Study. The Rotterdam Study is funded by Erasmus Medical
721 Center and Erasmus University, Rotterdam, Netherlands Organization for the Health Research and
722 Development (ZonMw), the Research Institute for Diseases in the Elderly (RIDE), the Ministry of
723 Education, Culture and Science, the Ministry for Health, Welfare and Sports, the European Commission
724 (DG XII), and the Municipality of Rotterdam. The authors are grateful to the study participants, the
725 staff from the Rotterdam Study and the participating general practitioners and pharmacists.

For peer review only

726 The authors are grateful Maz Osborne and John Stubbs for their consumer opinions on developing the manuscript.

728 **Availability of data and materials:** This research will be completed using data collected through the Australian Longitudinal study on Women's Health (https://alswh.org.au/), the 45 and Up Study (www.saxinstitute.org.au), and the Rotterdam Study (https://www.erasmusmc.nl/en/research/core-facilities/ergo-the-rotterdam-study). The data that support the analyses of this study depend on the availability of these data, which will be used under license for the current study, and so are not publicly available. Information on the process for accessing these data can be found in the websites mentioned above.

For peer review only

For peer review only - http://bmjopen.bmj.com/site/about/guidelines.xhtml

Table 1. Sources of data for the study

Data source	Socio-demographic characteristics	Lifestyle characteristics	Clinical characteristics	Reproductive Health	Family history of cancer and genetic predisposition	Health care utilisation	Diagnosis of Breast cancer	Medication use	Death
Self-report survey / clinical examinations	x	x	x	x	x		x	x	
PBS / Pharmacy data			x					x	
Cancer Registry data			x				x		
MBS data			x			x			
GP Records			x			x	x		
Hospital admission data			x			x	x		
Mortality register data									x

PBS: Pharmaceutical Benefits Scheme, MBS: Medicare Benefits Schedule, GP: general practitioner.

For peer review only

738 **Table 2. Variables for harmonised and cohort-specific analyses**

	ALSWH		45 and Up Study	Rotterdam Study			Harmonisable status
	1946-51 Cohort	1921-26 Cohort		RS-I-4	RS-II-2	RS-III-1	
**Socio-demographic characteristics**							
Age	Y	Y	Y	Y	Y	Y	Y*
Marital status	Y	Y	Y	Y	Y	Y	Y*
Country of birth	Y	Y	Y	N	N	N	N
Socioeconomic disadvantage	Y	Y	Y	Y	Y	Y	Y*
Remoteness	Y	Y	Y	NA	NA	NA	Y*
Work status	N	N	Y	Y	Y	Y	N
Education status	Y	Y	Y	Y	Y	Y	Y*
**Lifestyle characteristics**							
Body mass index (height/weight)	Y	Y	Y	Y	Y	Y	Y*
Physical activity	Y	Y	Y	N	N	Y	N
Smoking status	Y	N	Y	Y	Y	Y	N
Alcohol drinks per week	Y	N	Y	Y	Y	Y	N
Shift work at night	Y	N	N	N	N	N	N
**Clinical characteristics**							
High BP	Y	Y	Y	Y	Y	Y	Y*
Systolic BP/Diastolic BP	N	N	N	Y	Y	Y	N
Blood cholesterol level	N	N	N	Y	Y	Y	N
Diabetes	Y	Y	Y	Y	Y	Y	Y*
Heart disease	Y	Y	Y	Y	Y	Y	Y*
Stroke	Y	Y	Y	Y	Y	Y	Y*
Family history of heart disease	N	N	Y	Y	Y	Y	N
Family history of stroke	N	N	Y	Y	Y	Y	N
Mastectomy	Y	Y	Y	Y	Y	Y	Y*
**Reproductive health**							
Age at menarche	Y	N	N	Y	Y	Y	N
Age at menopause	Y	N	Y	Y	Y	Y	N
Age when had first child	Y	N	Y	Y	Y	Y	N
Number of children	Y	Y	Y	Y	Y	Y	Y*
History of hysterectomy/ both ovaries removed	Y	Y	Y	Y	Y	Y	Y*
Breastfeeding	N	N	Y	Y	Y	N	N
**Healthcare utilisation**							
Concession status	Y	Y	Y	NA	NA	NA	Y*
Mammogram	Y	N	Y	N	N	N	N
Hospital admissions	Y	Y	Y	N	N	N	N
Specialist/consulting physician attendances	Y	Y	Y	N	N	N	N
GPs attendances	Y	Y	Y	N	N	N	N
**Diagnosis of cancer**							

For peer review only - http://bmjopen.bmj.com/site/about/guidelines.xhtml

	ALSWH		45 and Up	Rotterdam Study			
A diagnosis/history of invasive breast cancer	Y	Y	Y	Y	Y	Y	Y*
A diagnosis/history of non-invasive breast cancer (LCIS, DCIS)	Y	Y	Y	Y	Y	Y	Y*
Other cancer	Y	Y	Y	Y	Y	Y	Y*
**Medicine use**							
CCB	Y	Y	Y	Y	Y	Y	Y*
Non-CCB (ACEI, ARB, BB, diuretics, others)	Y	Y	Y	Y	Y	Y	Y*
Statins	Y	Y	Y	Y	Y	Y	Y*
Current use of combined hormonal contraception	Y	Y	Y	Y	Y	Y	Y*
Current use of combined menopausal hormone therapy	Y	Y	Y	Y	Y	Y	Y*
Comorbidity (Rx-Risk) score	Y	Y	Y	Y	Y	Y	Y*
**Mortality**							
Death	Y	Y	Y	Y	Y	Y	Y*

ALSWH: Australian Longitudinal Study on Women's Health, Y: Yes, data available, N: No data available, NA: not applicable, BP: blood pressure, CVD: Cardiovascular disease, GPs: General practitioners, FNA: Fine-needle aspiration, LCIS: Lobular carcinoma in situ, DCIS: Ductal carcinoma in situ, CCB: Calcium channel blockers, ACEI: Angiotensin-converting-enzyme inhibitors, ARB: Angiotensin receptor blockers, BB: Beta blockers.

*: These variables will be used in the harmonised analysis. ** Comorbidity (Rx-Risk) score includes 46 clinical conditions based on prescribed medicine claims data.

Note: This table includes all potential confounders available from ALSWH (https://alswh.org.au/for-data-users/data-documentation/data-dictionary/), 45 and Up Study (https://www.saxinstitute.org.au/solutions/45-and-up-study/use-the-45-and-up-study/data-and-technical-information/), and Rotterdam Study (https://www.erasmusmc.nl/en/research/core-facilities/ergo-the-rotterdam-study).

For peer review only

List of figures

Figure 1. Schematic diagram showing entry, exposure, and exit of the study populations across longitudinal cohorts

Figure 2. Capture cumulative dose and duration exposed for each antihypertensive medicine

Figure 3. Sample size calculation

For peer review only

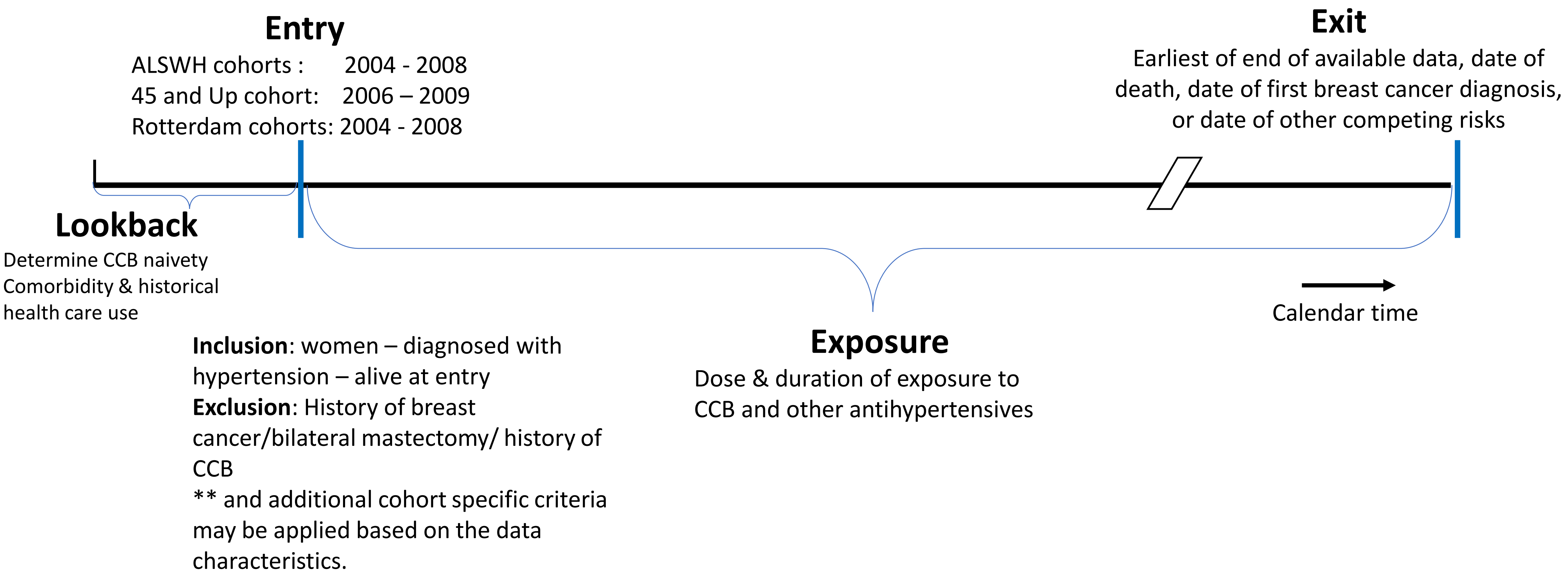

Figure 1. Schematic diagram showing entry, exposure, and exit of the study populations across longitudinal cohorts

ALSWH: Australian Longitudinal Study on Women's Health, CCB: Calcium channel blockers

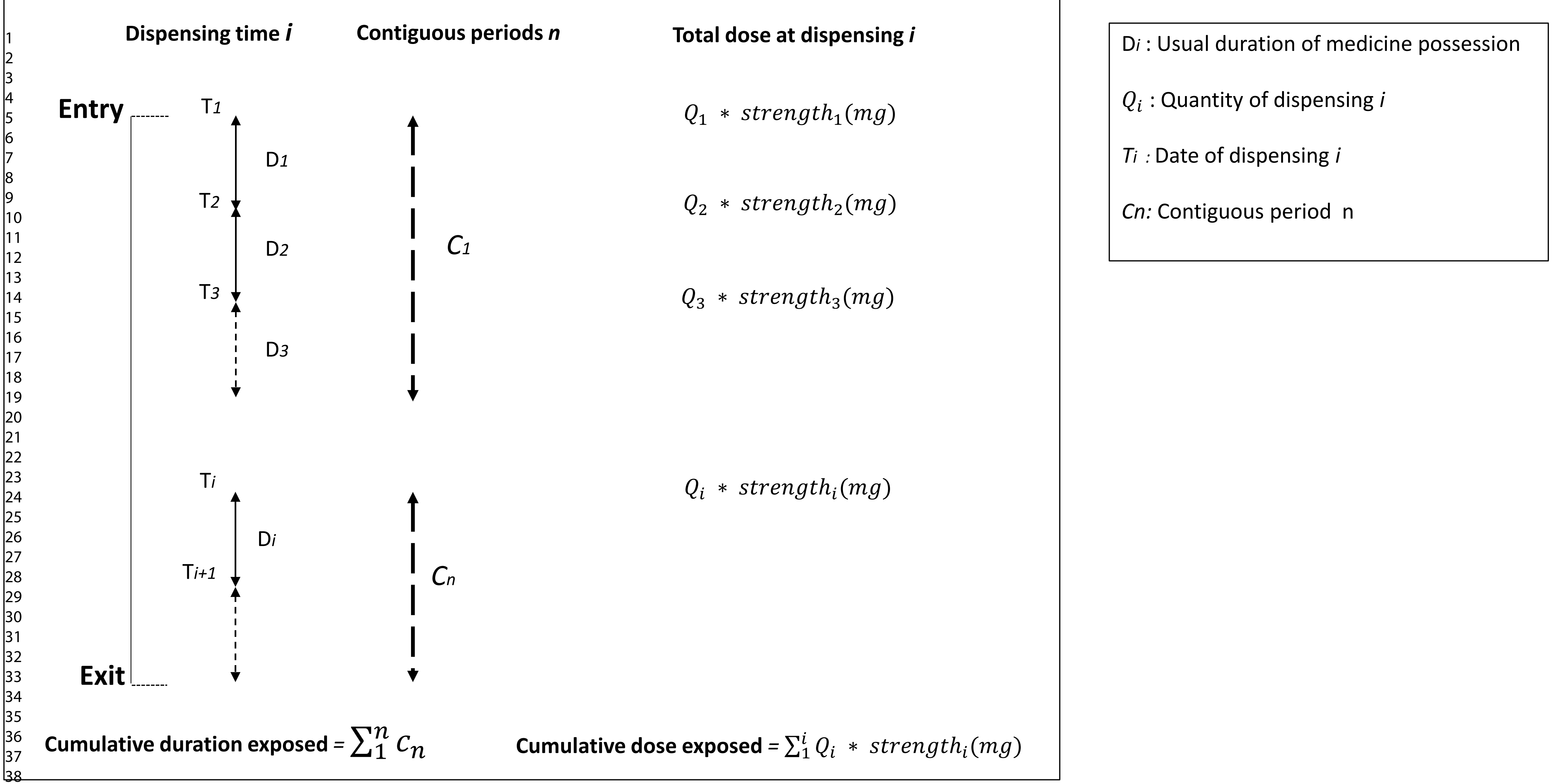

Figure 2. Capture cumulative dose and duration exposed for each antihypertensive medicine

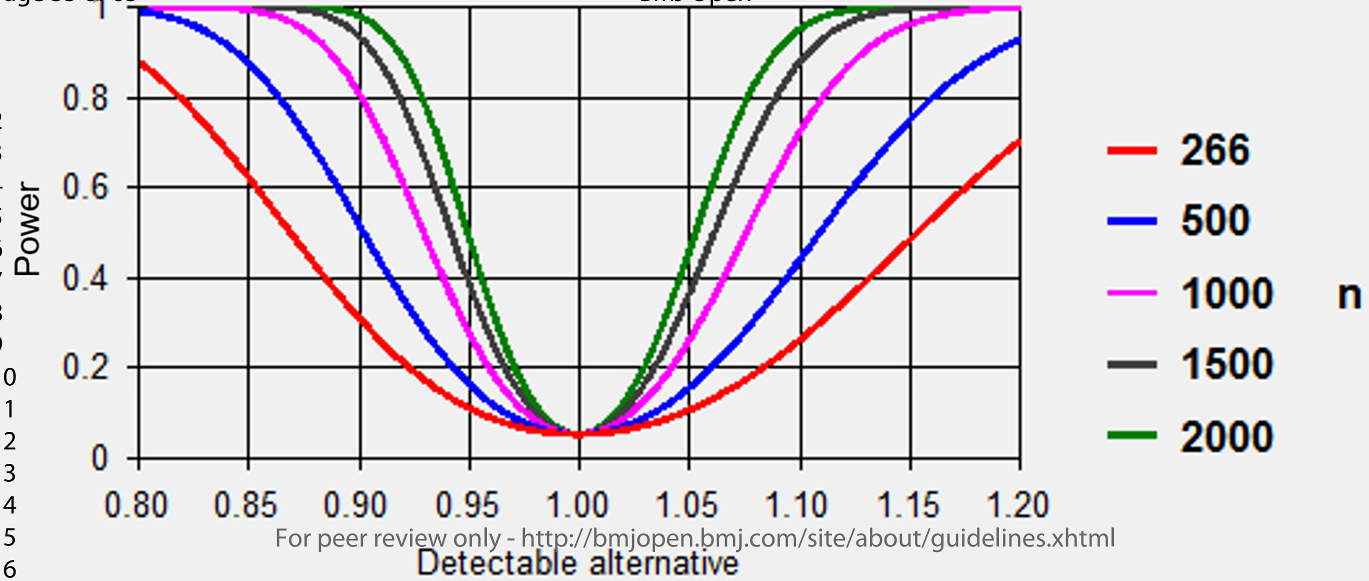

For peer review only - http://bmjopen.bmj.com/site/about/guidelines.xhtml

Title: Association between long-term use of calcium channel blockers (CCB) and the risk of breast cancer: A retrospective longitudinal observational study protocol

Appendix Table 1. Classification of prescribed oral antihypertensive medicines used based on the Anatomical Therapeutic Chemical codes

Class	ATC code	Drug name	Usual dose range mg/day	DDD (mg)	Usual daily frequency*
ACEIs	C09AA01	Captopril	25-100	50	2
	C09AA02	Enalapril	5-40	10	1-2
	C09AA09	Fosinopril	10-40	15	1
	C09AA03	Lisinopril	10-40	10	1
	C09AA04	Perindopril	4-8	4	1
	C09AA06	Quinapril	10-80	15	1-2
	C09AA05	Ramipril	2.5-20	2.5	1-2
	C09AA10	Trandolapril	1-4	2	1
	C09AA07	Benazepril	10-40	7.5	1
	C09AA13	Moexipril	7.5-30	15	1
ARBs	C09CA06	Candesartan	8-32	8	1
	C09CA02	Eprosartan	400-800	600	1-2
	C09CA04	Irbesartan	150-300	150	1
	C09CA01	Losartan	25-100	50	1-2
	C09CA08	Olmesartan Medoxomil	20-40	20	1
	C09CA07	Telmisartan	20-80	40	1
	C09CA03	Valsartan	80-320	80	1-2
BBs	C07AB03	Atenolol	25-100	75	1-2
	C07AB05	Betaxolol	5-20	20	1
	C07AB07	Bisoprolol	2.5-10	10	1
	C07AG02	Carvedilol	12.5-50	37.5	2
	C07AG01	Labetalol	200-800	600	2
	C07AB02	Metoprolol<br>Metoprolol ER	50-100<br>50-100	150<br>150	1-2<br>1
	C07AA12	Nadolol	40-120	160	1
	C07AB12	Nebivolol	5	5	1
	C07AA02	Oxprenolol	40-160	160	2
	C07AA03	Pindolol	10-40	15	2-3
	C07AA05	Propranolol<br>Propranolol LA	40-160<br>160-180	160<br>160	2<br>1
	C07AA06	Timolol	20-40	20	2
	C07AB04	Acebutolol	200-800	400	2
	C07AA23	Penbutolol	10-40	40	1
CCBs-DHP	C08CA01	Amlodipine	2.5-10	5	1
	C08CA02	Felodipine	2.5-20	5	1
	C08CA13	Lercanidipine	10-20	10	1
	C08CA05	Nifedipine<br>Nifedipine LA	10-40<br>20-120	30	2<br>1
	C08CA03	Isradipine	2.5-10	5	2
	C08CA04	Nicardipine ER	60-120	90	2

Class	ATC code	Drug name	Usual dose range mg/day	DDD (mg)	Usual daily frequency*
	C08CA07	Nisoldipine	10-40	20	1
	C10BX03	Atorvastatin And Amlodipine *	-	-	1
CCB-nonDHP	C08DA01	Verapamil<br>Verapamil LA<br>Verapamil IR	120-360<br>120-480<br>80-320	240<br>240<br>240	1<br>1-2<br>2
	C08DB01	Diltiazem ER<br>Diltiazem LA<br>Diltiazem	180-420<br>120-540<br>180-240	240	1<br>1<br>3-4
Diuretics	C03DB01	Amiloride	5-10	10	1-2
	C03EA01	Amiloride + HCTZ<br>Triamterene + HCTZ	-	-	1-2
	C03DB02	Triamterene	50-100	100	1-2
	C03AA04	Chlorothiazide	125-500	500	1-2
	C03AA05	Polythiazide	2-4	1	1
	C03AA01	Bendroflumethiazide	2.5-10	2.5	1
	C03AA03	HCTZ	12.5-50	25	1
	C03BA04	Chlortalidone	12.5-25	25	1
	C03BA11	Indapamide	1.25-2.5	2.5	1
	C03BA08	Metolazone**	0.5-1.0<br>2.5-5	5	1
	C03CA01	Furosemide **	20-80	40	2
	C03CA02	Bumetanide **	0.5-2	1	2
	C03CA04	Torsemide **	2.5-10	15	1
	C03DA01	Spironolactone **	25-50	75	1
	C03DA04	Eplerenone **	50-100	50	1-2
	C03CC01	Etacrynic acid	50-200	50	1-2
	C03XA01	Tolvaptan	15-60	30	1
Other ATH	C02AC01	Clonidine	0.1-0.8	0.5	2
	C02AC02	Guanfacine	0.5-2	3	1
	C02DB02	Hydralazine	25-100	100	2
	C02AB01	Methyldopa	250-1000	1000	2-4
	C02AA02	Reserpine	0.1-0.25	0.5	1
	C02DC01	Minoxidil	2.5-80	20	1-2
	C02AC05	Moxonidine	0.2-0.6	0.3	1-2
	C02CA01	Prazosin	2-20	5	2-3
	C02CA04	Doxazosin	1-16	4	1
	G04CA03	Terazosin	1-20	5	1-2
Combination	C09BB02	Lercanidipine + Enalapril	-	-	1
	C09BB04	Perindopril + Amlodipine	-	-	1
	C09BB05	Ramipril + Felodipine	-	-	1
	C09BB10	Trandolapril + Verapamil	-	-	1
	C09BA09	Fosinopril + HCTZ	-	-	1

For peer review only

Class	ATC code	Drug name	Usual dose range mg/day	DDD (mg)	Usual daily frequency*
	C09BA04	Perindopril + Indapamide	-	-	1
	C09BA02	Enalapril + HCTZ	-	-	1
	C09BA06	Quinapril + HCTZ	-	-	1
	C09DX04	Valsartan + Sacubitril	-	-	2
	C09DB01	Amlodipine + Valsartan	-	-	1
	C09DB02	Olmesartan + Amlodipine	-	-	1
	C09DB04	Telmisartan + Amlodipine	-	-	1
	C09DX01	Amlodipine + Valsartan + HCTZ	-	-	1
	C09DX03	Olmesartan + Amlodipine + HCTZ	-	-	1
	C09DA06	Candesartan + HCTZ	-	-	1
	C09DA02	Eprosartan + HCTZ	-	-	1
	C09DA04	Irbesartan + HCTZ	-	-	1
	C09DA08	Olmesartan + HCTZ	-	-	1
	C09DA07	Telmisartan + HCTZ	-	-	1
	C09DA03	Valsartan +HCTZ	-	-	1

HCTZ: hydrochlorothiazide. ATC: Anatomical Therapeutic Chemical, RAS: Renin Angiotensin System, CCB-DHP: calcium channel blockers-dihydropyridines, CCB-nonDHP: calcium channel blockers-nondihydropyridines, BB: beta blockers, ER: extended release, LA: long acting, IR: immediate release, DDD: Define Daily Dose, *: usual daily frequency is based on recommendations by the seventh report of the joint national committee on prevention, detection, evaluation, and treatment of high blood pressure (2003), **: These drugs are used for other indications (but have an antihypertensive effect).

For peer review only

Title: Association between long-term use of calcium channel blockers (CCB) and the risk of breast cancer: A retrospective longitudinal observational study protocol

Appendix Table 2. Codes used to identify participants' diagnosis and procedures in linked data sources

Conditions	MBS item number (for diagnoses or procedures recorded in Australian MBS data)	Procedure codes - ACHI 10 (for procedures recorded in Australian hospital admission data)	ATC codes (for medicines recorded in pharmacy data and study examination - Rotterdam)	ICD code (for diagnoses recorded in study examination-Rotterdam, hospital, cancer, and mortality data)	
				ICD-9-CM/ ICD-9	ICD-10-AM disease code/ ICD-10
**Diabetes**	MBS claims for:<br>- Diabetes annual cycle of care: 2517, 2518, 2521, 2522, 2525, 2526, 2620, 2622, 2624, 2631, 2633, 2635, 259 – 264.<br>- HbA1C test: 66551, 73840<br>- Comprehensive reassessment of eyes in a patient with diabetes mellitus: 10915, 12325, 12326.<br>- Diabetes education: 81100, 81105<br>- Exercise physiology health service for the management of type 2 diabetes: 81110, 81115<br>- Dietetics health service for the management of type 2 diabetes: 81120, 81125	-	+ Anti-diabetic Therapies: A10	+ Secondary diabetes melllitus:249<br>+ Diabetes mellitus: 250	+ Type 1 diabetes mellitus: E10<br>+ Type 2 diabetes mellitus: E11<br>+ Other specified diabetes Mellitus: E13<br>+Unspecified diabetes mellitus: E14
**Heart disease**	MBS claims for:<br>-Percutaneous transluminal coronary angioplasty, item 35304, 35305, 35310, 35335, 35338, 35341, 35344, 38300, 38303, 38306 38309, 38312, 38315, 38318.<br>- Coronary artery bypass graft, item 38497 – 38504.<br>- Selective coronary angiography, item 38215 – 38246	-	+ Digoxin: C01AA05<br>+ Isosorbide mononitrate: C01DA14<br>+ Glyceryl trinitrate: C01DA02	+ Ischemic heart disease: 410-414<br>+ Heart failure: 428	+ Ischemic heart disease: I20-I25<br>+ Heart failure: I50

peer review only

**Stroke**	-	-	-	+ Cerebrovascular diseases: 430-438	+ Cerebrovascular diseases: I60-I69
**Hysterectomy***	MBS claims for hysterectomy:<br>Laparoscopic abdominal hysterectomy: 35751, 35753, 35754, 35756<br>Abdominal hysterectomy: 35653, 35661, 35668, 35669, 35671, 35729<br>Vaginal hysterectomy replay: 35657, 35673, 35750	Laparoscopic abdominal hysterectomy: 90448-00, 90448–01, 90448–02<br>Abdominal hysterectomy: 35653-00, 35653–01, 35653–04, 35661–00, 35670–00, 35667–00, 35664–00.<br>Vaginal hysterectomy: 35750-00, 35753-02, 35657–00, 35673–02, 35667–01, 35664–01.<br>Pelvic exenteration: 90450–00, 90450–01, 90450–02	-	Laparoscopic abdominal hysterectomy: Abdominal hysterectomy: 68.3, 68.4, 68.6, Vaginal hysterectomy: 68.5, 68.7<br>Pelvic exenteration: 68.8<br>Other and unspecified hysterectomy: 68.9	-
**Oophorectomy***	-	Laparoscopic partial oophorectomy: 35638-01	-	Unilateral: 65.3, 65.4<br>Bilateral: 65.5, 65.6	-

For peer review only

		Unilateral: 35638-02, 35713-07<br>Bilateral: 35638-03, 35717-01			
**Mastectomy***	MBS claims for<br>+ Total mastectomy: 31519 (unilateral), 31520 (bilateral).<br>+ Subcutaneous Mastectomy: 31524, 31522 (skin-sparing, unilateral), 31523 (skin-sparing, bilateral), 31528 (nipple-sparing, unilateral), 31529 (nipple-sparing, bilateral)	+ Simple mastectomy: 31518-00 (unilateral), 31518-01 (bilateral).<br>+ Subcutaneous mastectomy: 31524-00 (unilateral), 31524-01 (bilateral)	-	+ Unilateral: 85.33, 85.34, 85.41, 85.43, 85.45, 85.47<br>+ Bilateral: 85.35, 85.36, 85.42, 85.44, 85.46, 85.48 or any combination of 2 mastectomy codes<br>+ Prophylactic mastectomy - mostly bilateral mastectomy: V50.41	+ Unilateral: Z90.12, Z90.11<br>+ Bilateral: Z90.13
**Specialist/consulting physician attendances**	MBS claims for:<br>Other non-referred attendances (not GP): 52, 53, 54, 57, 58, 59, 60, 65, 92, 93, 95, 96<br>Specialist attendances: 99, 104, 105, 106, 107, 108, 109, 113<br>Consultant physician attendances: 110, 112, 114, 116, 119, 122, 128, 131, 132, 133<br>Prolonged attendances: 160, 161, 162, 163, 164<br>Urgent attendances after hours: 598, 600<br>Emergency physician attendance: 501, 503, 507, 511, 515, 519, 520, 530, 532, 534, 536<br>Consultant psychiatrist attendances: 288, 289, 291, 293, 296, 297, 299, 300, 302,	-	-	-	-

	304, 306, 308, 310, 312, 314, 316, 318, 319, 320, 322, 324, 326, 328, 330, 332, 334, 336, 338, 342, 344, 346, 348, 350, 352, 353, 355, 356, 357, 358, 359, 361, 364, 366, 367, 369, 370<br>Pain and palliative medicine: 2799, 2801, 2806, 2814, 2820, 2824, 2832, 2840, 2946, 2949, 2954, 2958, 2972, 2974, 2978, 2984, 2988, 2992, 2996, 3000, 3003, 3005, 3010, 3014, 3015, 3018, 3023, 3028, 3032, 3040, 3044, 3051, 3055, 3062, 3069, 3074, 3078, 3083, 3088, 3093<br>Geriatric medicine: 141, 143, 145, 147, 149				
**GP Attendances**	MBS claims for:<br>GP Health Assessment: 93470, 93479<br>GP Chronic Disease Management Plan: 229, 230, 231, 232, 233, 93469, 93475<br>GP Multidisciplinary Case Conference: 235, 236, 237, 238, 239, 240, 243, 244, 735, 739, 743, 747, 750, 758<br>Medication Management Review (domiciliary): 245, 900<br>Medication Management Review (residential): 249, 903.<br>GP Mental Health: 272, 276, 277, 279, 281, 282, 894, 896, 898, 941, 942, 2121, 2150, 2196, 90264, 90265, 92112, 92113, 92114, 92115, 92116, 92117, 92118, 92119, 92120, 92121, 92122, 92123, 92124, 92125, 92126, 92127, 92128, 92129, 92130, 92131, 92132, 92133, 92134, 92135, 92146, 92147, 92148, 92149, 92150, 92151, 92152, 92153, 92154, 92155, 92156, 92157, 92158, 92159, 92160, 92161, 92170, 92171, 92176, 92177, 92182, 92184, 92186, 92188, 92194, 92196, 92198, 92200	-	-	-	-

For peer review only

	After-hours GP: 597-600 (urgent); 5000, 5020, 5040, 5060, 5200, 5203, 5207, 5208, 5003, 5010, 5023, 5028, 5043, 5049, 5063, 5067, 5220, 5223, 5227, 5228, 5260, 5263, 5265, 5267, 733, 737, 741, 745, 761, 763, 766, 769, 772, 776, 788, 789 (non-urgent)<br>GP – Practice Incentive Program services: 251, 252, 253, 254, 255, 256, 257 (cervical smear); 259, 260, 261, 262, 263, 264 (diabetes mellitus annual cycle of care); 265, 266, 268, 269, 270, 271 (asthma cycle of care)<br>GP short (level A): 3, 4, 2095, 2461, 90020, 91790, 91795, 91890<br>GP Standard (Level B): 23, 24, 2144, 2463, 90035, 91800, 91809, 91891<br>GP Long (Level C): 36, 37, 2180, 2464, 90043, 91801, 91810, 91894<br>GP Prolonged (Level D): 44, 47, 2193, 2465, 90051, 91802, 91811<br>Other Non-referred Medical Practitioner attendances: 899, 901, 905, 906, 90002, 91792, 91794, 91797, 91799, 91803, 91804, 91805, 91806, 91807, 91808, 91812, 91813, 91814, 91815, 91816, 91817, 91892, 91895, 92716, 92717, 92719, 92720, 92722, 92723, 92725, 92726, 92732, 92733, 92735, 92736, 92738, 92739, 92741, 92742, 92747, 93660, 93661, 93681, 93682, 93684, 93685, 93691, 93692, 93694, 93695, 93701, 93702, 93704, 93705<br>GP Focussed Psychological Strategies and Family Group Therapy: 283, 285, 286, 287, 371, 372<br>GP Acupuncture: 173, 193, 195, 197, 199<br>GP Telehealth (patient-end support): 812, 827, 829, 867, 868, 869, 873, 876, 881, 885, 891, 892				

For peer review only

	GP attendances relating to residential aged care facilities: 232, 249, 731, 772, 776, 788, 789, 829, 869, 881, 892, 903, 2125, 2138, 2179, 2220, 5010, 5028, 5049, 5067, 5260, 5263, 5265, 5267, 92102, 92071, 92058, 92027				
**Invasive breast cancer**	-	-	-	+ Invasive breast cancer: 174.x	+ Invasive breast cancer: C50.xxx
**Non-invasive breast cancer**	-	-	-	+ Non-invasive breast cancer: 233.0	+ Non-invasive breast cancer: D05.0, D05.01, D05.02 (lobular carcinoma in situ), D05.10, D05.11, D05.12 (Intraductal carcinoma in situ), D05.80, D05.81, D05.82 (other specified type of carcinoma in situ, breast), D05.90, D05.91, D05.92 (unspecified type of carcinoma in situ of breast)
**Unspecified breast cancer**	-	-	-	+ Personal history of malignant neoplasm of breast: V10.3<br>+ Secondary malignant neoplasm of the breast (cancer metastatic to the breast): 198.81	+ Personal history of malignant neoplasm of breast: Z85.3<br>+ Secondary malignant neoplasm of the breast (cancer metastatic to the breast): C79.81

For peer review only

**Other cancers (excluding breast cancer and non-melanoma skin cancers)**	-	-	-	140 - 172, 175 - 208	C00–C49, C51-C97, D45, D46, D47.1, D47.3–D47.5
**Statins use**	-	-	+ HMG CoA reductase inhibitors: C10AA<br>+ Combinations of various lipid modifying agents: C10BA<br>+ Lipid modifying agents in combination with other drugs: C10BX	-	-
**Combined hormonal contraceptives**	-	-	+ Progestogens and estrogens, fixed combinations: G03AA<br>+ Progestogens and estrogens, sequential preparations: G03AB<br>+ Vaginal ring with progestogen and estrogen: G02BB01<br>+ Cyproterone and estrogen: G03HB01	-	-
**Combined menopausal hormone therapy**	-	-	+ Progestogens and estrogens in combinations: G03F<br>+ Vaginal ring with progestogen and estrogen: G02BB01<br>+ Cyproterone and estrogen: G03HB01<br>+ Combinations of ATC codes for estrogens only and progestogens only:	-	-

For peer review only

			Estrogens: G03C Progestogens: G03D, Progestogen intrauterine device: G02BA03		
**Comorbidity (Rx- Risk score)**					
Alcohol dependency	-	-	Disulfiram, Naltrexone: N07BB01 - N07BB99	-	-
Allergies	-	-	Antihistamines (except hydroxyzine and diphenhydramine), nasal anti-inflammatories: R01AC01 -R01AD60, R06AD02 - R06AX27, R06AB04	-	-
Anticoagulants	-	-	Anticoagulants: B01AA03 - B01AB06, B01AE07, B01AF01, B01AF02, B01AX05	-	-
Antiplatelets	-	-	Antiplatelets: B01AC04 - B01AC30	-	-
Anxiety	-	-	Anxiolytics (benzodiazepines): N05BA01-N05BA12, N05BE01	-	-
Arrhythmias	-	-	Antiarrhythmics, Digoxin: C01AA05, C01BA01 - C01BD01, C07AA07	-	-
Benign prostatic hypertrophy	-	-	Alpha blockers: G04CA01-G04CA99, G04CB01, G04CB02 for men only		

For peer review only

Bipolar disorder	-	-	Lithium: N05AN01	-	-
Chronic airways disease	-	-	Inhaled bronchodilators: R03AC02-R03DC03, R03DX05	-	-
Congestive heart failure	-	-	Loop diuretics AND, ACE or ARB; Betablockers selective for heart failure. Aldosterone antagonists specific for heart failure: C03DA02–C03DA99, C07AB02—if PBS item code is 8732N, 8733P, 8734Q, 8735R. C07AB07, C07AG02, C07AB12, C03DA04 (C03CA01–C03CC01 and C09AA01–C09AX99, C09CA01–C09CX99 †		
Dementia	-	-	Anticholinesterases or other dementia medicines: N06DA02-N06DA04, N06DX01	-	-
Depression	-	-	Antidepressants: N06AA01-N06AG02, N06AX03- N06AX11, N06AX13-N06AX18, N06AX21-N06AX26	-	-
Diabetes			Insulins, oral Hypoglycaemics: A10AA01–A10BX99		
Epilepsy	-	-	Anticonvulsants: N03AA01-N03AX99	-	-

For peer review only

Glaucoma	-	-	Topical antiglaucoma Agents: S01EA01-S01EB03, S01EC03-S01EX99	-	-
Gastric acid disorder	-	-	Histamine H2-receptor antagonists, Proton-pump inhibitor: A02BA01-A02BX05	-	-
Gout	-	-	Antigout agents: M04AA01-M04AC01	-	-
Hepatitis B	-	-	Anti Hep B antivirals: J05AF08, J05AF10, J05AF11	-	-
Hepatitis C	-	-	Interferon/ribavirin combinations, Protease inhibitors for hepatitis: J05AB54, J05AE11-J05AE12, J05AE14, J05AX14, J05AX15, J05AX65, J05AB04 L03AB10, L03AB11, L03AB60, L03AB61	-	-
HIV	-	-	Anti-HIV antivirals: J05AE01-J05AE10, J05AF01-J05AF07, J05AF09, J05AF12-J05AG05, J05AR01-J05AR99, J05AX07-J05AX09 J05AX12	-	-
Hyperkalaemia	-	-	Sodium polystyrene Sulfonate: V03AE01	-	-
Hyperlipidaemia	-	-	Antilipaemic agents: C10AA01-C10BX09, A10BH03‡		
Hypertension	-	-	Thiazides, potassium sparing agents,		

For peer review only

			combination antihypertensives, other antihypertensives: C03AA01–C03BA11, C03DB01- C03DB99, C03EA01, C09BA02–C09BA09, C09DA02–C09DA08, C02AB01–C02AC05, C02DB02–C02DB99 (C03CA01–C03CCO1 or C09CA01–C09CX99)§		
Hyperthyroidism	-	-	Antithyroid Preparations: H03BA02, H03BB01	-	-
Hypothyroidism	-	-	Thyroid replacements: H03AA01-H03AA02	-	-
Irritable bowel syndrome	-	-	IBS specific drugs, rectal anti-inflammatories: A07EC01–A07EC04, A07EA01–A07EA02, A07EA06, L04AA33	-	-
Ischemic heart disease: Angina	-	-	Nitrates: C01DA02-C01DA14, C01DX16, C08EX02		
Ischemic heart disease: Hypertension			C07AA01–C07AA06, C07AA08–C07AB01, C07AB02—if PBS item code is not 8732N, 8733P, 8734Q, 8735R, C07AG01, C08CA01–C08DB01, C09DB01–C09DB04, C09DX01, C09BB02–C09BB10,		

For peer review only

			C07AB03, C09DX03, C10BX03¶		
Incontinence	-	-	G04BD01–G04BD99		
Inflammation/pain	-	-	NSAIDS: M01AB01-M01AH06	-	-
Liver failure	-	-	Rifaximin: A06AD11, A07AA11	-	-
Malignancies	-	-	Antineoplastics agents (excluding topical): L01AA01-L01XX41	-	-
Malnutrition	-	-	Enteral nutritional Supplements: B05BA01-B05BA10	-	-
Migraine	-	-	Antimigraine Medications: N02CA01-N02CX01	-	-
Osteoporosis/Paget's	-	-	Alendronate, etidronate: M05BA01-M05BB05, M05BX03, M05BX04, G03XC01, H05AA02	-	-
Pain	-	-	Opiate containing Medications: N02AA01–N02AX02, N02AX06, N02AX52, N02BE51		
Pancreatic insufficiency	-	-	Pancreatic exocrine enzyme replacement: A09AA02	-	-
Parkinsons disease	-	-	Antiparkinson agents: N04AA01-N04BX02	-	-
Psoriasis	-	-	Systemic and topical antipsoriatics: D05AA01-D05AA99, D05BB01-D05BB02, D05AX02, D05AC01-D05AC51, D05AX52	-	-

For peer review only

Psychotic illness	-	-	Antipsychotics: N05AA01-N05AB02, N05AB06-N05AL07, N05AX07-N05AX13	-	-
Pulmonary hypertension	-	-	Antihypertensives for pulmonary arterial hypertension: C02KX01–C02KX05, PBS item code 9547L, 9605M	-	-
Renal disease	-	-	Alpha erythropoietin, calciferol, calcitriol, sevelamer: B03XA01-B03XA03, A11CC01-A11CC04, V03AE02, V03AE03, V03AE05	-	-
Smoking cessation	-	-	Nicotine, Bupropion: N07BA01-N07BA03, N06AX12	-	-
Steroid-responsive conditions	-	-	Glucocorticoids: H02AB01-H02AB10	-	-
Transplant	-	-	Immune suppressants: L04AA06, L04AA10, L04AA18, L04AD01, L04AD02	-	-
Tuberculosis	-	-	Anti-tubercular agents: J04AC01 - J04AC51, J04AM01 - J04AM99	-	-

MBS: Medicare Benefits Schedule, PBS: Pharmaceutical Benefits Scheme, AM: Australia Modification, ICD: International Classification of Diseases, FNA: Fine-needle aspiration, ACHI: The Australian Classification of Health Interventions. Notes: MBS codes and procedure codes are exclusively used for Australian data. *: Procedures conducted out of hospital or in private hospitals are recorded in MBS data by MBS item number whereas those conducted in public hospitals are captured in the hospital admission data by procedure codes. †: Must have at least two medicines prescribed with one of those medicines having an ATC code from C03CA01–C03CC01 and the other having an ATC code from either C09AA01–C09AX99 or C09CA01–C09CX99. ‡: Combination product for hyperlipidaemia and diabetes. §: Can have medicine dispensed with an ATC code C03CA01–C03CC01 or C09AA01–C09AX99, but not both, as this would indicate chronic heart failure. ¶: Combination product for hyperlipidaemia and ischaemic heart disease: hypertension

For peer review only

Title: Association between long-term use of calcium channel blockers (CCB) and the risk of breast cancer: A retrospective longitudinal observational study protocol

Appendix Table 3. Description and response categories of data items from baseline surveys of the ALSWH Study, the 45 and Up Study and Rotterdam Study

Table A. The Australian Longitudinal Study on Women's Health Study (ALSWH)

Description of data item	Question	Response categories
**Socio-demographic characteristics**		
Age	BOTH COHORTS: What is your date of birth?	Date variable (DD/MM/YYY)
Marital status	BOTH COHORTS: What is your present marital status?	+ 1946-51 COHORT: Married; de facto (in a relationship); widowed; separated; divorced; never married<br>+ 1921-26 COHORT: Married (registered); de facto relationship (opposite sex); de facto relationship (same sex); separated; divorced; widowed; never married
Country of birth	BOTH COHORTS: In which country were you born?	BOTH COHORTS: Australia; UK; Ireland; Italy; Greece; New Zealand; Vietnam; other (please specify)
	BOTH COHORTS: If you were not born here, when did you first arrive in Australia with the intention of living here for one year or more?	+ 1946-51 COHORT: 1955 or earlier; 1956-1965; 1966-1985; 1986-1990; 1991 or later<br>+ 1921-26 COHORT: 1935 or earlier; 1936-1945; 1946-1955; 1956-1965; 1966-1985; 1986-1990; 1991 or later
Education status	BOTH COHORTS: What is the highest qualification you have completed?	BOTH COHORTS: No formal qualifications; school or intermediate certificate (or equivalent); higher school or leaving certificate (or equivalent); trade/apprenticeship (e.g. hairdresser, chef); certificate/diploma (e.g. child care, technician); university degree; higher university degree (e.g. Grad Dip, Masters, PhD)
**Lifestyle characteristics**		
Body mass index (height/weight)	BOTH COHORTS: How tall are you without shoes?	Continuous variable (height in cm)
	BOTH COHORTS: How much do you weigh without clothes or shoes?	Continuous variable (weight in kg)

For peer review only

Description of data item	Question	Response categories
Physical activity	BOTH COHORTS:<br>If you add up all the time you spent doing each activity LAST WEEK, how much time did you spend ALTOGETHER doing each type of activity?<br>1. Walking briskly (for recreation or exercise or to get to or from places)<br>2. Moderate leisure activity (like social tennis, moderate exercise classes, recreational swimming, dancing)<br>3. Vigorous leisure activity (that make you breathe harder or puff and pant like aerobics, competitive sport, vigorous cycling, running, swimming)<br>3. Vigorous household or garden chores (that make you breathe harder or puff and pant)	Continuous variable (time in hours/minutes)
Smoking status	1946-51 COHORT: How often do you currently smoke cigarettes or any tobacco products?	1946-51 COHORT: Daily; at least weekly (but not daily); less often than weekly; not at all
Alcohol drinks per week	1946-51 COHORT: How often do you usually drink alcohol?	1946-51 COHORT: I never drink alcohol; I drink rarely; less than once a week; on 1 or 2 days a week, on 3 or 4 days a week; on 5 or 6 days a week; every day
	1946-51 COHORT: On a day, when you drink alcohol, how many drinks do you usually have	1946-51 COHORT: 1 or 2 drinks per day; 3 or 4 drinks per day; 5 to 8 drinks per day; 9 or more drinks per day
Shift work at night	1946-51 COHORT: Do you normally do paid work at night?	1946-51 COHORT: Yes; no
**Clinical characteristics**		

For peer review only

Description of data item	Question	Response categories
High BP	BOTH COHORTS: Have you ever been told by a doctor that you have hypertension (high blood pressure)?	BOTH COHORTS: Yes; no
	BOTH COHORTS: In the past 3 years, have you been diagnosed or treated for high blood pressure?	BOTH COHORTS: Yes; no
Diabetes	BOTH COHORTS: Have you ever been told by a doctor that you have diabetes?	BOTH COHORTS: Yes; no
	BOTH COHORTS: In the past 3 years, have you been diagnosed or treated for diabetes (high blood sugar)?	BOTH COHORTS: Yes; no
	1946-51 COHORT: Have you ever been told by a doctor that you have non-insulin dependent (type 2) diabetes?	1946-51 COHORT: Yes; no
	1946-51 COHORT: In the past 3 years, have you been diagnosed or treated for non-insulin dependent (type 2) diabetes?	1946-51 COHORT: Yes; no
	1921-26 COHORT: In the last 3 years, have you been told by a doctor that you have diabetes?	1921-26 COHORT: Yes; no
Heart disease	BOTH COHORTs: Have you ever been told by a doctor that you have heart disease?	BOTH COHORTs: Yes; no
	1946-51 COHORT: In the past 3 years, have you been diagnosed or treated for heart disease?	1946-51 COHORT: Yes; no
	1921-26 COHORT: In the past 3 years, have you been diagnosed or treated for angina/heart attack/other heart problems?	1921-26 COHORT: Yes; no
Stroke	BOTH COHORTS: Have you ever been told by a doctor that you have stroke?	BOTH COHORTS: Yes; no

For peer review only

Description of data item	Question	Response categories
	BOTH COHORTS: In the past 3 years, have you been diagnosed or treated for stroke?	BOTH COHORTS: Yes; no
	1921-26 COHORT: In the last 3 years, have you been told by a doctor that you have stroke	1921-26 COHORT: Yes; no
**Reproductive health**		
Age at menarche	1946-51 COHORT: At what age did your period start?	Continuous variable (Age in years)
Age at menopause	1946-51 COHORT: Have you had a period or menstrual bleeding in the last 12 months?	1946-51 COHORT: Yes; no
	1946-51 COHORT: If you have reached menopause, at what age did your periods completely stop?	Continuous variable (Age in years)
Age when had first child	1946-51 COHORT: If you have given birth, please write the year of the birth in the birth (if you had twins, please write the date twice)	Continuous variable (Year)
Number of children	1946-51 COHORT: If you have given birth, please write the year of the birth in the birth (if you had twins, please write the date twice)	Continuous variable (Year)
History of hysterectomy/ both ovaries removed	BOTH COHORTS: Have you ever had hysterectomy?	BOTH COHORTS: Yes; no
	BOTH COHORTS: In the PAST THREE YEARS, have you had hysterectomy?	BOTH COHORTS: Yes; no
	BOTH COHORTS: Have you ever had both ovaries removed?	BOTH COHORTS: Yes; no
	BOTH COHORTS: In the PAST THREE YEARS, have you had both ovaries removed?	BOTH COHORTS: Yes; no

For peer review only

Description of data item	Question	Response categories
**Healthcare utilisation**		
Concession card holders	1946-51 COHORT: Do you have a Health Care Card? (This is a card that entitles you to discounts and assistance with medical expenses. This is not the same as a Medicare card)	1946-51 COHORT: Yes; no
Mammogram	1946-51 COHORT: When did you last have a mammogram?	1946-51 COHORT: I have never had a mammogram; 2 years ago or less; more than 2 but less than 5 years ago; 5 or more years ago; not sure
	1946-51 COHORT: Have you ever had an abnormal mammogram?	1946-51 COHORT: No; Yes in the last 5 years; Yes more than 5 years ago
	1946-51 COHORT: In the last two years, have you had a mammogram?	1946-51 COHORT: Yes; no
**Diagnosis of cancer**		
A diagnosis/history of breast cancer	BOTH COHORT: Have you ever been told by a doctor that you have breast cancer?	BOTH COHORT: Yes; no
	1946-51 COHORT: In the past 3 years, have you been diagnosed or treated for breast cancer?	1946-51 COHORT: Yes; no
	1921-26 COHORT: In the last 3 years, have you been told by a doctor that you have breast cancer?	1921-26 COHORT: Yes; no
**Medicine use**		
Current use of hormonal contraception	1946-51 COHORT: Are you currently taking the oral contraceptive pill for any reason?	1946-51 COHORT: Yes; no
Current use of menopausal hormone therapy	1946-51 COHORT: Are you currently on hormone replacement therapy (HRT)?	1946-51 COHORT: Yes; no

BP: blood pressure

For peer review only

Table B. The 45 and Up Study

Description of data item	Question	Response categories
**Socio-demographic characteristics**		
Age	What is your date of birth?<br>What is today's date?	Date variable (DD/MM/YYYY)
Marital status	What best describes your current situation?	Single; married; de facto/living with a partner; widowed; divorced; separated
Country of birth	In which country were you born?	Australia; UK; Ireland; Italy; China; Greece; New Zealand; Germany; Lebanon; Philippines; Netherlands; Vietnam; Malta; Poland; other (please specify)
	What year did you first come to live in Australia for one year or more?	Continuous variable (Year)
Work status	What is your current work status? (you can cross more than one box)	In full time paid work; self-employed; in part time paid work; doing unpaid work; completely retired/pensioner; studying; partially retired; looking after home/family; disabled/sick; unemployed; other
	If you are partially or completely retired, how old were you when you retired?	Continuous variable (Age in years)
	Why did you retire?	Reached usual retirement age; lifestyle reasons; to care for family member/friend; ill health; made redundant; could not find a job; other
Education status	What is the highest qualification you have completed?	No school certificate or other qualifications; school or intermediate certificate (or equivalent); higher school or leaving certificate or equivalent); trade/apprenticeship (e.g. hairdresser, chef); certificate/diploma (e.g. child care, technician); university degree or higher
**Lifestyle characteristics**		
Body mass index (height/weight)	How tall are you without shoes?	Continuous variable (Centimetres, Feet, Inches)
	About how much do you weigh?	Continuous variable (Kilograms; Stone; Pounds)
Physical activity	If you add up all the time you spent doing each activity LAST WEEK, how much time	Walking continuously; Vigorous physical activity; Moderate physical activity

For peer review only

Description of data item	Question	Response categories
	did you spend ALTOGETHER doing each type of activity?	
Smoking status	Have you ever been a regular smoker?	Yes; no
	Are you a regular smoker now?	Yes; no
Alcohol drinks per week	About how many alcoholic drinks do you have each week?<br>On how many days each week do you usually drink alcohol?	Continuous variable (Integer)
**Clinical characteristics**		
High BP/ Diabetes/ Heart Disease/ Stroke/ Breast cancer/ Other cancer	Has a doctor EVER told you that you have:	Skin cancer (not melanoma); Melanoma; Breast cancer (F); Prostate cancer (M); Other cancer (free text); Heart disease (free text); High blood pressure - when pregnant (F); High blood pressure - when not pregnant (F); High blood pressure (M); Stroke; Diabetes; Blood clot (thrombosis); Enlarged prostate (M); Asthma; Hay fever; Depression; Anxiety; Parkinson's disease; None of these
Family history of heart disease/ stroke/ breast cancer/ ovarian cancer	Have your mother, father, brother(s) or sister(s) ever had:	Heart disease; High blood pressure; Stroke; Diabetes; Dementia/Alzheimer's; Parkinson's disease; Severe depression; Severe arthritis; Breast cancer; Bowel cancer; Lung; Melanoma; Prostate cancer; Ovarian cancer; Osteoporosis; Hip fracture; Do not know
**Reproductive health**		
Age at menopause	Have you been through menopause?	No; not sure (because hysterectomy, taking HRT, etc.); my periods have become irregular; yes
	If yes, how old were you when you went through menopause?	Continuous variable (Age in years)
Age when had first child	How old were you when you gave birth to your FIRST child?	Continuous variable (Age in years)
Number of children	How many children have you given birth to?	Continuous variable (Age in years)
History of hysterectomy/ both ovaries removed	Have you ever had hysterectomy?	Yes; no
	Have you ever had both ovaries removed?	Yes; no

For peer review only

Description of data item	Question	Response categories
Breastfeeding	For how many months, in total, have you breastfed?	Continuous variable (number of months)
**Healthcare utilisation**		
Concession card holders	Which of the following do you have? (excluding Medicare)	Private health insurance-with extra; private health insurance – without extras; Department of Veterans' Affairs white or gold card; Health care concession card; none of these.
Mammogram	Have you ever been for a breast screening mammogram?	Yes; no
	If Yes, what year did you have your last mammogram?	Continuous variable (Year)
	How many times have you been for breast screening altogether?	Continuous variable (Integer)
**Medicine use**		
Current use of hormonal contraception	Have you ever used the pill or other hormonal contraceptives? (e.g. the combined pill, mini pill, contraceptive implant or injections)	Yes; no
	If Yes, how old were you when you LAST used hormonal contraceptives? (please write your current age if you are still using them)	Continuous variable (Age in years)
Current use of menopausal hormone therapy	Have you ever used hormone replacement therapy (HRT)?	Yes; no
	Are you currently taking HRT?	Yes; no

F: female, M: male, BP: blood pressure, HRT: hormone replacement therapy

For peer review only

Table C. Rotterdam Study

Description of data item	Question	Response categories
**Socio-demographic characteristics**		
Age	Date of birth was provided by the municipality, and age was subsequently computed by data management	Continuous variable (Age in years)
Marital status	*Home interview:* What is your marital status?	Never been married; married (once); widowed, then remarried; divorced and remarried; never been married, lives with unmarried spouse; widowed, lives with unmarried spouse; divorced, lives with unmarried spouse; widowed; divorced
Work status	*Home interview:* What is your current employment status?	Employed; unemployed, out of job; house wife/man; unfit for work; rentier; early retirement; retirement
Education status	*Home interview:* What is your highest attained education?	Primary education; lower vocational education; intermediate secondary education; intermediate vocational education; general secondary education; higher education (Higher professional education); higher education (University)
**Lifestyle characteristics**		
Body mass index (height/weight)	*Clinical examination:* Weight was assessed with the participant wearing light indoor clothes, without shoes. Height was measured.	Weight in kilograms, height in centimetres
Smoking status	*Home interview:* Do you smoke cigarettes?	No; yes; don't know; no answer
	Did you quit smoking ever?	No; yes; don't know; not applicable; no answer
	Did you use to smoke cigarettes earlier?	No; yes; don't know; not applicable; no answer
	Did you smoke cigars? Did you ever smoke cigars or did you smoke cigars but quitted now?	No, never; ever little cigars; ever big cigars; ever both; yes little; yes big; yes both; don't know; no answer
Alcohol drinks per week	How many glasses of beer/wine/spirit do you drink a day?	Continuous variable (Integer)

Description of data item	Question	Response categories
**Clinical characteristics**		
High BP	Clinical exam: Hypertension	No; Yes;
Diabetes	Clinical exam: Diabetes	No; Yes;
Systolic BP	Clinical exam: Systolic BP	Continuous variable
Diastolic BP	Clinical exam: Diastolic BP	Continuous variable
Blood cholesterol level	Clinical exam: Blood drawn, clinical lab.	Continuous variable (Value)
Family history of heart disease	*Home interview:*<br>Did one of your parents or siblings experience a heart attack?	No; yes
**Reproductive health**		
Age at menarche	*Home interview:*<br>At what age did your period start?	Continuous variable (Age in years)
Age at menopause	*Home interview:*<br>At what age had your periods stopped definitively?	Continuous variable (Age in years)
Age when had first child	*Home interview:*<br>In what year was your first child born?	Continuous variable (Age in years, calculated by data management team)
Number of children	*Home interview:*<br>How many pregnancies (of at least 6 months) have you had?	Continuous variable (Integer)
History of hysterectomy	*Home interview:* Tick box, yes no	No; Yes
History of both ovaries removed	*Home interview:* Tick box, yes no	No; Yes

BP: blood pressure. Clinical exams were undertaken via automated coupling of the study database with medical records from general practitioners.

For peer review only

Standard Protocol Items for Observational Studies (SPIROS)

Table 1. SPIROS 2023 Checklist: Recommended Items to address in the observational study Protocol and related documents

Section/ item	Description	Addressed on page number
**Part A: general information**		
Title	Descriptive title Identifying study design in the title	Page 1
Protocol version	Version or amendment number with date and summary of the changes	Document name
Protocol summary	An informative and balanced summary of the study protocol	Page 2
Sponsor and funder details	Name of Sponsor and funder and types of financial, material, and other support	Page 26
Conflict of interest statements	Statement about any financial and other competing interests for principal or co investigators for the overall study.	Page 26
Investigators name	Names of the principal and co-investigators	Page 1
Affiliation of investigators	Affiliated institutions of the investigators	Page 1
Principal researcher/s contact detail	Name, e-mail address, affiliation of principal researcher	Page 1
**Part B: Introduction**		
Background of the study	Description of research question and scientific background of the study	Page 4
Review of prior research	Summary of relevant existing research (published or unpublished)	Page 4-6
Rationale of study	Justification for conducting the study	Page 5-6
Aim	Broader aims and overall objective	Page 6
Objective/s of the study	Primary and secondary objective/s including any prespecified hypothesis (if applicable).	Page 6
	Specify whether the intention is to (a) estimate causal effects, (b) predict outcomes, or (c) simple description.	Page 6
**Part C: Methods**		
Study design	Description of study design (case control, cross-sectional or cohort) and type of study (retrospective cohort study, Prospective cohort study etc)	Page 7
Study setting	Description of the study setting (e.g., community-based, hospital based) and detail of precise locations of the study sites.	NA

For peer review only

Study schedule	Description of the expected schedule of the study including relevant dates, expected periods of recruitment/survey, exposure, follow-up, and data collection.	Page 8-9
	Figure (Study schematic/flow-chart) or table describing expected time frame for each step including trainings, data collection, follow-up, analysis and reporting etc.	Figure 1
Sample size	Estimation of minimum sample size required for the study with justifications including clinical and statistical assumptions supporting any sample size calculations.	Page 16, figure 3
Sampling procedure	Detailed description of the sampling frame and sampling strategy (simple random, stratified random, cluster, systematic etc.)	NA
Participant selection		
Participant selection for cohort study	Description of inclusion and exclusion criteria, and the source and methods of participant selection (exposed and unexposed). For matched cohort studies, give matching criteria and number of exposed and unexposed.	Page 6-9
Participant selection for case-control study	Description of inclusion and exclusion criteria, and the source and methods of case ascertainment and control selection. Give the rationale for the choice of cases and controls. Give diagnostic criteria for identifying cases (if applicable). For matched case-control studies, give matching criteria and the number of controls per case.	NA
Participant selection for cross-sectional study	Description of the inclusion and exclusion criteria, and the source and methods of participant selection.	NA
Variables	Detailed description of all important baseline and outcome variables to be analysed, exposures, predictors, potential confounders, and effect modifiers. Give diagnostic criteria, if applicable.	Figure 2, appendix table 1-2
Data sources/measurement	For each variable of interest, give sources of data and details of assessment /measurement methods. Describe comparability of assessment methods if there is more than one group.	Table 1
Data collection and management	Plans for assessment and collection of outcomes, baseline, follow up and other study related data.	Page 10-13
	Description of data collection methods e.g., online survey, Household survey, paper based or electronic data capture etc.	Page 10-13
	Any related processes to promote data quality during data	NA

For peer review only

	collection (e.g., duplicate measurements, training of assessors, validation method)	
	Description of study instruments (e.g., questionnaires, data collection forms) along with their reliability and validity, if known. Reference to where data collection forms can be found, if not in the protocol.	Appendix table 3
	Plans for data entry, coding, security, and storage, including any related processes to promote data quality (e.g., electronic data capture, double data entry; range checks for data values, random cross-checking of electronic data with the source documents).	NA
	Reference to where details of data management procedures can be found, if not in the protocol.	NA
Blinding procedure (if blinded study)	Description of blinding procedure (if applicable) reporting Who will be blinded (e.g., investigator blinded for disease status when measuring exposure in case-control study) and methods to ensure blinding and unmasking of blinding if required.	NA
Potential bias	Description of any potential biases and plan to minimize those potential sources of biases.	Page 19-20
Statistical analysis plan	Detailed description of methods for analysing and presenting primary/secondary outcomes and any additional analysis (e.g. analyses of subgroups and interactions, and sensitivity analyses). Give reference to the where other details of the statistical analysis plan can be found, if not in the protocol.	Page 14-16
Handling of missing data	Detailed description of methods to handle missing data (e.g. multiple imputation).	NA
Handling of withdrawals and lost to follow up	Detailed description of the procedures to be followed when a participant ceases participation in the study prematurely or is lost to follow up	Page 9
Replacements	Plans and methods of the replacement or substitution of withdrawn participants.	NA
Outcome	Definition and description of all primary, secondary and other outcomes.	Page 12-13
Data confidentiality statement	A detailed description of process to ensure data confidentiality.	NA
Follow up	A detailed plan of follow up including schedule and methods (telephonic, house based, hospital based etc.) of follow up.	NA

For peer review only

Plan of study monitoring	Description of plan for study monitoring and whether the monitoring will be independent from investigators or sponsors.	NA
Training of surveyors/data collectors	Description of how investigators and surveyors will be trained to conduct the research activity.	NA
Quality assurance	Plan of quality assurance. Back-checking data collection.	NA
**Part D: Ethical consideration**		
Ethical approval	Plan for seeking ethics approval from ethics committees/institutional review boards. If known, give name of ethical committees.	Page 16-17
	If ethics approval will not be sought, give justification.	NA
Consent and assent	Description of who will obtain informed consent or assent from potential study participants or authorized surrogates, and how (e.g., written informed consent, verbal consent, video/audio recording of consent procedure etc.)	Page 17
	Give reason if consent or assent not sought.	NA
	Give reference to where informed consent forms and applicable translations plan can be found, if not in the protocol.	NA
Risk/harm to participants	A detailed description of potential risks or harms to study participants.	NA
	Plans for collecting, assessing, reporting, and managing any study procedures related adverse events (e.g. adverse events due to blood collection) and other unintended effects of study conduct (e.g. risk to breach confidential and sensitive information of participants)	NA
	Give a statement about whether data will be anonymous, pseudonymized, or can be directly linked to participants.	NA
	Description of any plan for giving Incentives to the participants	NA
Adverse event and serious adverse event reporting	Outline how adverse events and serious adverse events information will be collected and reported.	NA
Involvement of patient/participant representatives in protocol development	Patient and Public Involvement (PPI) statement including how patients or participants involved in the planning of the study. Give statement, if there is no plan to involve of patient/participants and public in designing or any phase of the study	Page 16
**Part E: Reporting and dissemination**		

For peer review only

Dissemination/ publication plan	Plans for investigators and sponsor to communicate study results to ethical review boards, participants, key stake holders, the public, and other relevant groups.	Page 17
	Methods to communicate findings (e.g., via publication (open access or closed access), reporting in results databases, or other data-sharing arrangements), including any publication restrictions.	Page 17
	Define authorship eligibility guidelines (e.g., ICMJE recommendations)	Page 26
**Part F: Others**		
Whether Artificial Intelligence (AI) assisted technology was used in writing the protocol	Disclose whether authors used artificial intelligence (AI)- assisted technologies in the production of protocol (e.g., chatbots) or there is planning to use artificial intelligence (AI)- assisted technologies in the production of manuscript or study reports.	NA
	Give the name of AI tools (such as ChatGPT). Include a statement if authors did or did not review and edited the content created by AI-assisted technologies	NA
References	A complete list of references cited in protocol	Page 21-25
Funding	Source of any funding for the study and the role of the funders for the study	Page 26
Open science	Registration of observational study: Study identifier and registry name (e.g., open science framework, ClinicalTrials.gov, ICTRP or any other national or international study registry platform). If not yet registered, name of intended registry.	Page 2
	Data sharing: Plans, if any, for granting public access to the (1) full protocol and amendments, (2) participant-level data set, (3) Statistical analysis plan, (4) statistical codes and other study material (e.g., case report forms, study questionnaires and Informed consent forms). Give reference to where these documents can be found, if not included as annex in the protocol.	Page 27

For peer review only
